# Single-Cell Sequencing Analysis and Multiple Machine Learning Methods Identified G0S2 and HPSE as Novel Biomarkers for Abdominal Aortic Aneurysm

**DOI:** 10.3389/fimmu.2022.907309

**Published:** 2022-06-13

**Authors:** Tao Xiong, Xiao-Shuo Lv, Gu-Jie Wu, Yao-Xing Guo, Chang Liu, Fang-Xia Hou, Jun-Kui Wang, Yi-Fan Fu, Fu-Qiang Liu

**Affiliations:** ^1^ Department of Cardiovascular, Shaanxi Provincial People’s Hospital, Xi’an, China; ^2^ Department of Cardiovascular Surgery, Yan'an Affiliated Hospital of Kunming Medical University, Kunming, China; ^3^ Department of Cardiovascular Surgery, China-Japan Friendship Hospital, Beijing, China; ^4^ Department of Cardiothoracic Surgery, Affiliated Hospital of Nantong University, Nantong, China; ^5^ Department of Pathology, College of Basic Medical Sciences China Medical University, Shenyang, China; ^6^ School of Biological Science and Medical Engineering, Beihang University, Beijing, China

**Keywords:** abdominal aortic aneurysm, single-cell sequencing, weighted co-expression network analysis, differentially expressed genes, multiple machine learning methods

## Abstract

Identifying biomarkers for abdominal aortic aneurysms (AAA) is key to understanding their pathogenesis, developing novel targeted therapeutics, and possibly improving patients outcomes and risk of rupture. Here, we identified AAA biomarkers from public databases using single-cell RNA-sequencing, weighted co-expression network (WGCNA), and differential expression analyses. Additionally, we used the multiple machine learning methods to identify biomarkers that differentiated large AAA from small AAA. Biomarkers were validated using GEO datasets. CIBERSORT was used to assess immune cell infiltration into AAA tissues and investigate the relationship between biomarkers and infiltrating immune cells. Therefore, 288 differentially expressed genes (DEGs) were screened for AAA and normal samples. The identified DEGs were mostly related to inflammatory responses, lipids, and atherosclerosis. For the large and small AAA samples, 17 DEGs, mostly related to necroptosis, were screened. As biomarkers for AAA, G0/G1 switch 2 (*G0S2*) (Area under the curve [AUC] = 0.861, 0.875, and 0.911, in GSE57691, GSE47472, and GSE7284, respectively) and for large AAA, heparinase (HPSE) (AUC = 0.669 and 0.754, in GSE57691 and GSE98278, respectively) were identified and further verified by qRT-PCR. Immune cell infiltration analysis revealed that the AAA process may be mediated by T follicular helper (Tfh) cells and the large AAA process may also be mediated by Tfh cells, M1, and M2 macrophages. Additionally, *G0S2* expression was associated with neutrophils, activated and resting mast cells, M0 and M1 macrophages, regulatory T cells (Tregs), resting dendritic cells, and resting CD4 memory T cells. Moreover, *HPSE* expression was associated with M0 and M1 macrophages, activated and resting mast cells, Tregs, and resting CD4 memory T cells. Additional, *G0S2* may be an effective diagnostic biomarker for AAA, whereas *HPSE* may be used to confer risk of rupture in large AAAs. Immune cells play a role in the onset and progression of AAA, which may improve its diagnosis and treatment.

## Introduction

Abdominal aortic aneurysm (AAA) is a cardiovascular disease defined by aortic dilation exceeding 50% of the normal aortic diameter, which can lead to aortic rupture and bleeding. Until a rupture event occurs, the patient is usually asymptomatic (1). The incidence of AAA has risen dramatically as the age of the world population increases. Sudden mortality from aneurysm rupture occurs in 60–85% cases, posing a major threat to the health of middle-aged and elderly people (2). Aneurysms with a diameter > 5.5 cm, that rapidly dilate over a short period of time and compromise perfusion to distant organs, are candidates for open surgery or endovascular aortic repair. However, only using these measurements as biomarkers is not high accuracy (3). Continuous monitoring of AAA biological activity is important for reducing mortality and morbidity associated with rupture; however, there is no effective predictive biomarker of AAA diameter (4) or treatment that prevents AAA from growing or rupturing (5, 6). Therefore, it is critical that we elucidate the underlying mechanisms of AAA progression to accurately identify appropriate therapeutic targets.

Single-cell sequencing is a ground-breaking approach that permits the clustering of cells to investigate gene expression variances across groups and differences in cell progression (7, 8). AAA permits the accurate investigation of genetic characteristics at the single-cell level, since it contains a wide variety of cells, including monocytes, T cells, mast cells, and B cells (9). Weighted co-expression network analysis (WGCNA) is a method used in systems biology to explore gene interaction patterns across multiple samples (10). It may be used to find highly co-variant gene sets as well as potential biomarker genes or therapeutic targets based on the gene set interconnectivity and association with clinical characteristics (11).

We utilized public databases to conduct AAA single-cell sequencing, WGCNA analysis, and differential expression analysis to investigate gene variants in AAA and discover possible therapeutic and diagnostic targets. We also performed an additional GEO dataset to confirm our findings. With the fast development of gene microarray technology, along with bioinformatics analysis, gene chip technology can provide a novel and effective technique to investigate the molecular mechanisms of many diseases (12). However, few studies have utilized machine learning approaches, such as the least absolute shrinkage and selection operator (LASSO), support vector machine-recursive feature elimination (SVM-RFE), and Random Forest (RF) to uncover large AAA biomarkers. To screen for important variables and establish the optimal classification model, LASSO analysis uses an L1-penalty (lambda) to set the coefficients of less significant variables to zero. It uses supervised machine learning to categorize data points by maximizing the distance between classes in a high-dimensional space (13). RF is a non-parametric classification method (14), which includes decision trees based on divided data sets. It is, therefore, widely used to discover biomarkers and predict models with excellent accuracy and interpretability. In this context, we used a RF classification model to discover characteristics that may distinguish AAA from a normal sample.

Moreover, CIBERSORT was used to compare the tissue immune infiltrates of 22 immune cell subsets between AAA and normal samples, as well as between small and large AAAs. Additionally, the relationships between diagnostic markers and infiltrating immune cells were investigated to acquire a better understanding of the molecular immunological processes underlying AAA development. The findings of this study will enable the identification of novel diagnostic biomarkers and therapeutic targets for AAA and improve our understanding of its pathogenesis.

## Materials and Methods

### Data Processing

We obtained four AAA RNA chip datasets [GSE7084 (15), GSE47472 (16), GSE57691 (17), and GSE98278 (18)] and one AAA single-cell RNA-sequencing dataset [GSE166676 (8)] from GEO (http://www.ncbi.nlm.nih.gov/geo). Following standardization, samples without clinical information were excluded. Hereby, 15 samples were obtained in GSE7084 (8 normal and 7 AAA samples), 22 samples in GSE47472 (8 normal and 14 AAA samples), 59 samples in GSE57691 [10 normal, 20 small AAA (mean maximum aortic diameter = 54.3 ± 2.3 mm) and 29 large AAA samples (mean maximum aortic diameter = 68.4 ± 14.3 mm)], 31 samples in GSE98278 [15 small AAA (mean maximum aortic diameter ≤ 55 mm)], and 16 large AAA samples (mean maximum aortic diameter > 70 mm). Individual genes in the GSE7084, GSE47472, GSE57691, and GSE98278 datasets were further annotated by respective planforms. GSE7084 was used for further validation and was assessed in comparison with normal and AAA samples. The GSE98278 sample was also used for validation and was evaluated in comparison with small AAA and large AAA samples. The characteristics of the five datasets are shown in **Table 1**.

**Table 1:** Characteristics of the five datasets.

Characteristics of the datasets			
Datasets	AAA	Normal	Platform
GSE57691	49 (29 large, 20 small)	10	GPL10558
GSE47472	14	8	GPL10558
GSE7084	7	8	GPL2507
GSE166676	4	2	GPL24676
GSE98278	31 (16 large, 15 small)	0	GPL10558

AAA, abdominal aortic aneurysm. large, mean maximum aortic diameter > 55 mm; small, mean maximum aortic diameter ≤55 mm.

### Single-Cell Quality Control and Dimensions Reduction

We identified cells expressing more than 200 genes but no more than 2,500 genes. Meanwhile 10% of mitochondrial genes and 3% of red blood cell genes were set as cut off value to further filtrated. After identifying 3,000 hypervariable genes for analysis, the number of principle components (PCs) was adjusted to 13 to generate cell clusters that were then exhibited and annotated using the “tSNE” diagram. We next selected the top ten different expression genes in each cluster using the “FindAllmarkers” function from Seurat R Package. Then 20 clusters in total were discovered (**Supplementary Table 1**).

### Differential Gene Analysis and Cell Type Annotation

We utilized the R package “SingleR” to annotate our single-cell RNA-seq data automatically. Between the expression profiles of each cell and those of the reference sample, Spearman’s correlation was calculated. We then defined the score for each label as the set quantile of the correlation distribution (0.8 by default). We repeated this approach for all labels, using the label with the highest score as the cell’s annotation.

We utilized the “FindMarkers” method to identify genes that differed significantly between AAA and normal cells. **Supplementary Table 2** contains a list of all significant indicators that distinguish AAA from normal cells.

### Pseudotime Analysis

After annotating all cells, we extracted all monocyte objects and randomly selected mean expressions > 0.1 & dispersion empirical > 1 * dispersion fit cells for subsequent pseudotime analysis. Subsequently, using the “DDRTree” approach, we reduced the dimension of cells and then determined the kind of cell differentiation state using the “reduceDimension” function. Finally, we employed the “plot cell trajectory” function to visualize the differentiation trajectory of cells.

### Weighted Co-Expression Network Analysis in GSE47472

We performed a WGCNA analysis on GSE47472 and then utilized selected genes with a standard deviation of expression > 0 for further analysis, excluding outlier data. The data were divided into distinct modules by setting an optimal soft threshold (**Supplementary Table 3**) and simultaneously identifying the modules that were most positively associated with AAA.

### DEGs Identification in GSE57691

GSE7691 was used for a differential analysis. The “limma” R Package was employed to investigate the differences between the AAA and normal sample groups post normalization. The results were presented as a heat map. After applying a filter (| logFC | > 0.5 and adjusted p < 0.05), we obtained DEGs (**Supplementary Table 4**) and displayed by volcano graph.

Additionally, we then applied a filter (p < 0.05) to obtain DEGs (**Supplementary Table 5**) and present the differences between large and small AAA samples as a volcano plot.

### Functional and Pathway Enrichment Analysis of DEGs

We conducted functional enrichment analysis of the DEGs from GSE57691. All key terms were clustered according to membership similarity, and the term with the highest degree of enrichment was chosen as the representative. To investigate the functions and pathways of DEGs, we utilized the “clusterProfiler” R Package (v4.0) to perform gene ontology (GO) and Kyoto Encyclopedia of Genes and Genomes (KEGG) pathway enrichment. Statistical significance was set at p < 0.05.

### Screening and Verification of Diagnostic Markers Between Normal and AAA

Three methods were applied to identify novel and key biomarkers for AAA: single-cell differential gene analysis, identification between AAA and normal cells, and WGCNA (19). We then utilized “FindMarkers” to perform single-cell differential gene analysis, “limma” for DEG identification, and “WGCNA” for WGCNA analysis (10). For additional investigation, we chose overlapping genes from three previously described models. The GSE7084 dataset was utilized as a validation set for in-depth assessing the efficacy of important biomarkers. This was performed using a receiver operating characteristic (ROC), and the area under the curve (AUC) was calculated to establish the biomarkers’ predictive ability. Statistical significance was determined using a two-sided P < 0.05.

### Screening and Verification of Diagnostic Markers Between Small and Large AAA

Random Forest (RF) (14), LASSO (20), and SVM-RFE (21) were performed to screen for novel large AAA biomarkers. We used the “randomForest” R package for RF and “glmnet” R Package to perform LASSO logistic regression with low lambda. This research used the RF function in the “caret” package to pick prominent genes using ten-fold cross validation. The SVM classifier was created using the R package “e1071”. The three classifiable models’ overlapping genes were then figured out. GSE98278 was used as the validation set for conducting an in-depth evaluation of the effectiveness of significant biomarkers. The validation was performed using ROC analyses, and the algorithm’s prediction ability was measured using AUC. A two-sided P < 0.05 was used to determine statistical significance.

### Immune Infiltration Analysis

We analysed the level of immune cell infiltration between AAA and normal samples of GSE57691 (**Supplementary Table 6**) and small and large AAA samples of GSE57691 (**Supplementary Table 7**) using the CIBERSORT analysis technique with the parameter “PERM” set to 1000 and a cutoff of p < 0.05. Additionally, the proportion of each type of immune cell in the samples was computed and was shown using a bar plot. The “pheatmap” package was used to create a heat map of the 22 immune cells, and abundance was shown using the “vioplot” package. Using the “corrplot” package, we created a correlation heatmap to visualize the correlation between 22 different infiltrating immune cells.

### Infiltrating Immune Cells Interact with Diagnostic Markers

A Spearman’s rank correlation test in R was used to examine the relationship between infiltrating immune cells and finally gained gene biomarkers. Correlations were illustrated using ”ggplot2” package.

### Quantitative PCR Analysis

A total of 9 tissue samples (including 3 normal samples and 3 small AAA samples and 3 large AAA samples) were collected from Shanxi Provincial People’s Hospital. The Ethical Committee of Shanxi Provincial People’s Hospital approved this study and the respective patient provided informed consent in a written form. Total RNA was isolated using TRIzol reagent (Invitrogen, USA) following the manufacturer’s protocol, and RNA purity was detected using a NanoDrop 2000 spectrometer (Thermo Fisher Scientific, MA, USA). The quantitative real-time polymerase chain reaction (PCR) was performed Based on SuperReal PreMix Plus (Invitrogen) in a StepOnePlus Real-time PCR Detection System (Applied Biosystems, CA, USA), with the following primers: GAPDH (forward: 5’-GGACCTGACCTGCCGTCTAG-3’, reverse: 5’-GTAGCCCAGGATGCCCTTGA-3), HPSE(forward: 5’-TGCTATCCGACACCTTTGC-3’, reverse: 5’-TTGCCTCATCACCACTTCTAT-3’), G0S2 (forward: 5’-CCTCTTCGGCGTGGTGCTC-3’, reverse: 5’-CTGCTGCTTGCCTTTCTCC–3’) synthesized by shanghai GENE ray Biotech. GAPDH was then handled as an internalreference. The relative expression was calculated using the2^(-△Ct) method. P values <0.05 showed statistical significance.

### Statistical Analysis

All statistical analyses were conducted using R. A student’s t-test was used to compare AAA and normal samples, as well as small and large AAA samples. ROC analysis was performed to estimate the discriminatory value of marker genes. Statistical significance was set at p < 0.05, unless otherwise specified.

## Results

**Figure 1:**
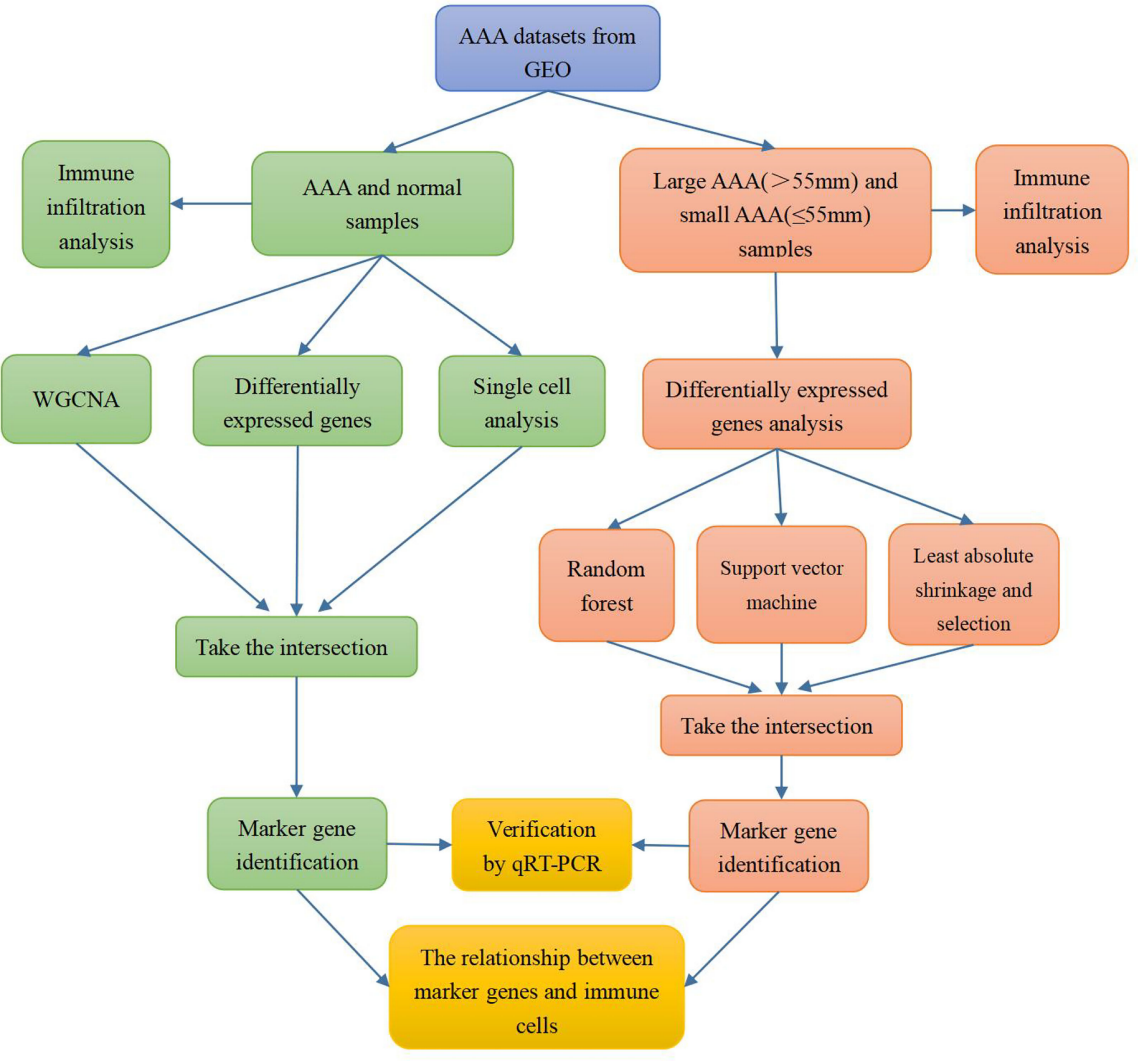
Flow chart of the research process.

The flow chart of our study is shown in **Figure 1**.

### Single-Cell Quality Control and Dimension Reduction Clustering

**Figure 2:**
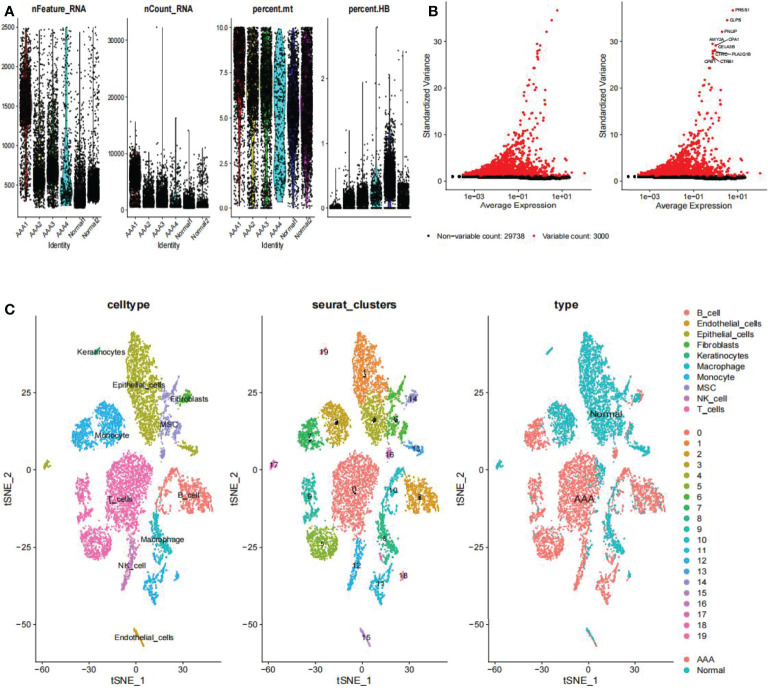
Clustering of GSE166676 single cells for quality control and dimension reduction. **(A)** The proportion of mitochondrial and erythrocyte genes is adjusted to ensure the quality of cell samples. **(B)** 3,000 highly variable genes are indicated in red, with the 10 most important emphasized. **(C)** Reduced dimensionality and cluster analysis. The AAA dataset cells may be classified into 20 clusters, which include B cells, endothelial cells, fibroblasts, keratinocytes, macrophages, monocytes, mesenchymal stem (MSC), natural killer (NK) cells, and T cells. AAA, abdominal aortic aneurysm.

On the single-cell dataset, a quality control procedure was performed. As shown in **Figure 2A**, we removed certain cells and controlled the fraction of mitochondrial and red blood cell genes to assure the quality of the cell samples used in the research. Thereafter, we identified 3,000 genes with high variability and labeled the 10 most important ones. All hypervariable genes are highlighted in red in **Figure 2B**. As shown in **Figure 2C**, the cells were classified into 20 clusters, with the different clusters being roughly classified as B and endothelial cells, fibroblasts, keratinocytes, macrophages, monocytes, mesenchymal stem (MSC), natural killer (NK) cells, and T cells. Clusters 2 and 10 were identified as B cells; clusters 1, 4, 13, 15, and 17 as endothelial cells; cluster 14 as fibroblasts; cluster 8 as macrophages; clusters 3, 7, 11, and 18 as monocytes; and clusters 6 and 16 as mesenchymal stem cells (MSCs). Cluster 12 was identified as a NK cells; T cells were identified in clusters 0, 5, and 9; cluster 19 was identified as keratinocytes. In epidermal keratinocytes, we observed an increased expression of genes involved in HA synthesis, including *PTPRC* and *HAS1*. According to the original text of this dataset, low-dose UVB irradiation stimulated hyaluronan synthesis in epidermal keratinocytes by sequential stimulation of hyaluronan synthases Has1-3, which was mediated by p38 and Ca2+/calmodulin-dependent protein kinase II (CaMKII) signaling (22). Finally, we assigned red and blue colors to the AAA and normal samples, respectively.

### Differential Gene Analysis and Pseudotime Analysis

**Figure 3:**
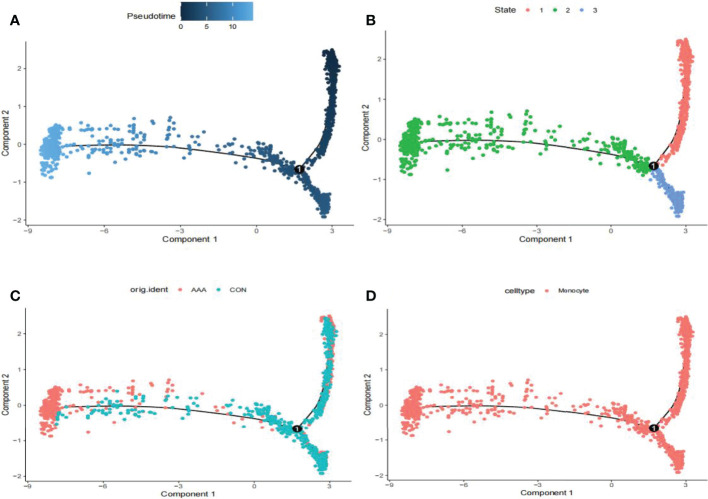
Analyses of pseudotime in GSE166676. **(A)** Timing differences in cell differentiation. Darker blue represents an earlier stage of differentiation, while a lighter blue indicates a later stage of differentiation. This serves as a starting point for subsequent analysis. **(B)** Three stages of monocyte differentiation. State 1 is the earliest stage of differentiation. **(C)** Differentiation of AAA monocytes from normal monocytes. **(D)** All cells analyzed were monocytes. AAA, abdominal aortic aneurysm.

We utilized the “FindMarkers” method to identify genes that differed significantly between AAA and normal cells. We performed a simulation analysis on the cell trajectory differentiation of all monocytes and discovered that the darker the blue, the earlier the cell differentiation, indicating that monocytes differentiate from right to left over time, with the lightest blue representing the most recently differentiated cells (**Figure 3A**). As shown in **Figure 3B**, there were three distinct differentiated states of monocytes, each labeled with a different color, with the red one (on the right) being the earliest differentiated type. We subsequently investigated the differentiation process of AAA and normal cells and discovered that AAA monocytes differentiated earlier than normal monocytes (**Figure 3C**). All cells analyzed were monocytes (**Figure 3D**).

### Weighted Co-Expression Network Analysis

**Figure 4:**
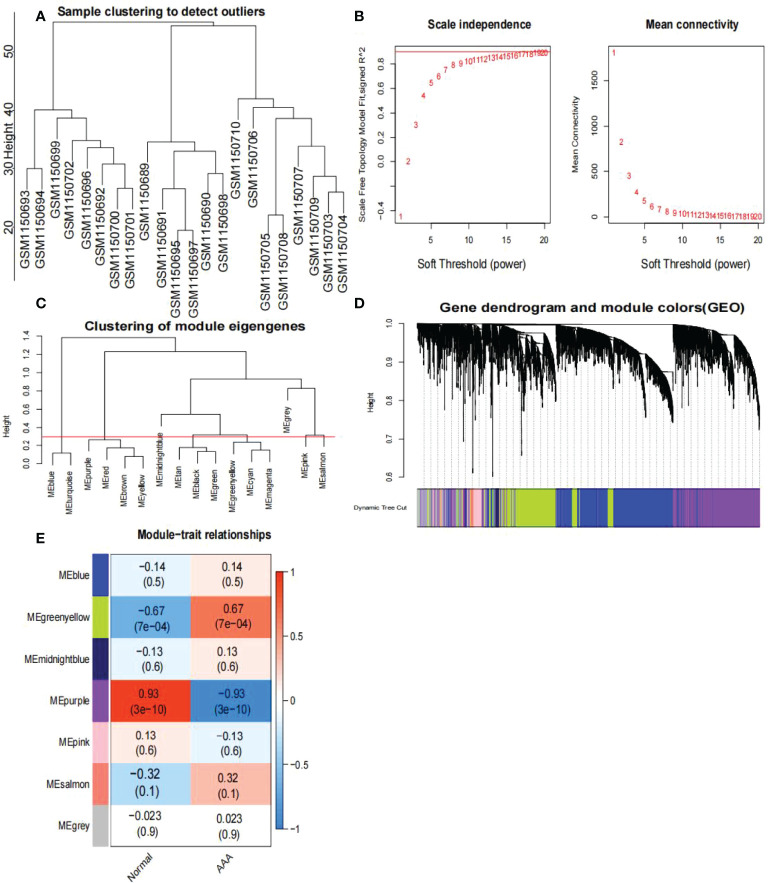
Analysis of the weighted co-expression network in GSE47472. **(A)** Sample clustering of dataset GSE47472. The samples were classified into three clusters that were significantly distinct. All clusters were chosen for further analysis. **(B)** Selection of optimal thresholds. The threshold is 19. **(C)** Set the threshold to 0.3 to merge modules that are comparable in the cluster tree. **(D)** Different modules are produced and shown in different colors by aggregating genes with strong correlations into a same module. Blue modules make up a greater proportion. **(E)** Analysis of correlations between modules and AAA. The green-yellow module was significantly correlated with AAA (COR = 0.67, P < 0.001) and with normal samples (COR = -0.67, P < 0.001). The WGCNA-hub genes were provided to the genes in the green-yellow module. AAA, abdominal aortic aneurysm.

First, we identified 15,471 genes with a standard deviation of expression greater than zero. Second, the “flashClust” tool package was used to perform the cluster analysis with a threshold of 70; Cluster 1 had 26 samples, which we retained (**Figure 4A**). Third, the power parameter range of 1–20 was filtered using the “pickSoftThreshold” function of the “WGCNA” package, and we used a power of β = 19 (scale-free R2 = 9) as the soft threshold to establish a scale-free network (**Figures 4B, C**). To merge similar modules in cluster 3, we set the threshold to 0.3 (**Figure 4D**); the minimum number of modules was set to 50. Seven modules were produced, each containing genes with similar co-expression characteristics (**Figure 4E**). As revealed by module-trait association analyses, multiple modules were associated with AAA (**Figure 4E**), with the green-yellow module being the most significant, including 1,286 genes.

### Differential Gene Analysis in GSE57691

**Figure 5:**
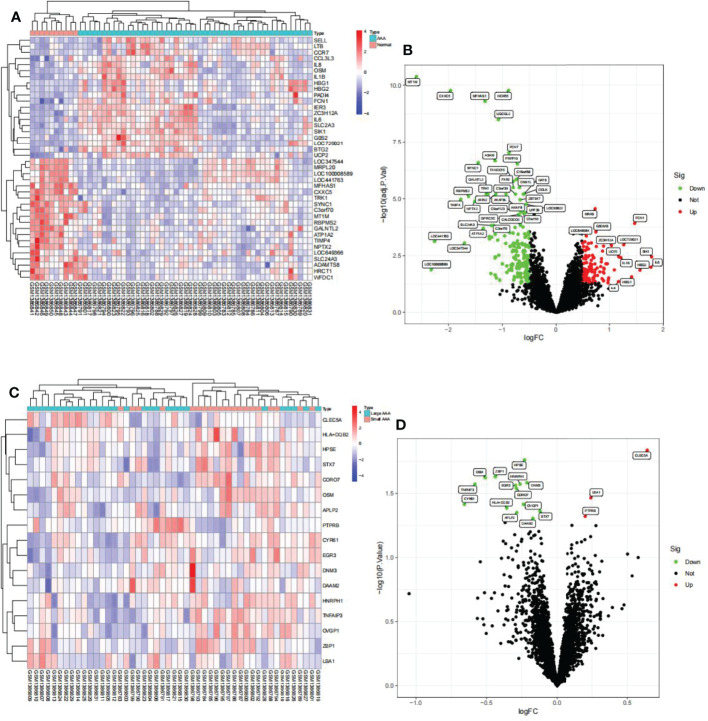
Visualization of the DEG findings in GSE57691. **(A)** Heatmap clustering of genes with markedly different expression in AAA compared normal samples. |log2Foldchange|>0.5 and adjusted P-value < 0.05 were used to define statistically significant DEGs. AAA: abdominal aortic aneurysm; DEGs, differentially expressed genes. Cyan denotes AAA samples, whereas red-orange denotes normal samples. **(B)** DEGs volcano map; red denotes upregulated genes, black represents genes with no significant difference, and green represents downregulated genes. **(C)** Heatmap clustering of genes with substantially different expression levels in large AAA compared small AAA samples. P-value < 0.05 was used to determine statistically significant DEGs. AAA: abdominal aortic aneurysm; DEGs, differentially expressed genes. Cyan indicates large AAA samples, whereas red-orange indicates small samples. **(D)** DEG volcano map; red denotes upregulated genes, black represents genes with no significant difference, and green represents downregulated genes.

We identified 288 differentially expressed genes (DEGs) between AAA and normal samples in GSE57691, including 111 upregulated and 177 downregulated genes (**Figures 5A, B**). Additionally, we identified 17 DEGs between the large and small AAA samples in GSE57691, three of which were upregulated and 14 downregulated, (**Figures 5C, D**). We discovered that the expression of these genes varied significantly between samples, with the deep red hue indicating a greater expression level (**Figures 5A, C**). In **Figures 5B, D**, DEGs are shown as a volcano map, with blue and red representing genes that were expressed at low and high levels, respectively, in AAA samples. Additionally, blue and red represent genes with low and high abundance, respectively, in large AAA samples.

### Functional Enrichment Analysis of DEGs

**Figure 6:**
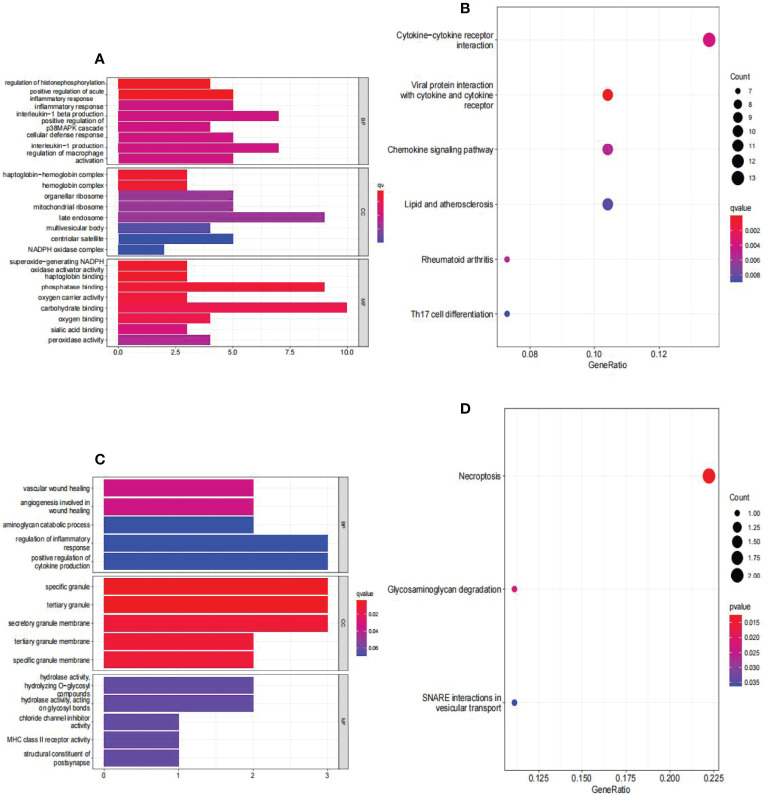
Analyses of functional enrichment of DEGs in GSE57691. **(A)** Analysis of DEGs between AAA and normal samples using Gene Ontology (GO) enrichment analysis. The x-axis indicates the number of genes associated with the terms, while the y-axis indicates the pathway terms. Each term’s q-value is colored according to the legend. BP, biological process; CC, cellular component; MF, molecular function; **(B)** enrichment analysis of DEGs between AAA and normal samples using the Kyoto Encyclopedia of Genes and Genomes (KEGG). Each term’s q-value is colored according to the legend. Different colored bubbles reflect different pathway terms. **(C)** Enrichment analysis of DEGs between large AAA and small AAA samples using Gene Ontology (GO). The x-axis indicates the number of genes associated with the terms, while the y-axis indicates the pathway terms. Each term’s q-value is colored according to the legend. BP, biological process; CC, cellular component; MF, molecular function; **(D)** Enrichment analysis of DEGs comparing large AAA and small AAA samples using the Kyoto Encyclopedia of Genes and Genomes (KEGG). Each term’s q-value is colored according to the legend. Different colored bubbles reflect different pathway terms. AAA, abdominal aortic aneurysm.

The findings of the GO analysis were classified into three categories: biological processes, cell components, and molecular functions. For AAA and normal samples (**Figure 6A**), the DEGs were enriched in biological processes, such as regulation of histone phosphorylation and positive regulation of acute inflammatory response; cell components, such as the haptoglobin–hemoglobin and hemoglobin complexes; and molecular functions, such as superoxide-generating NADPH oxidase activator activity, haptoglobin binding, phosphatase binding, and oxygen carrier activity. The KEGG pathway was enriched for viral protein interaction with cytokine and cytokine receptor, cytokine-cytokine receptor interaction, chemokine signaling pathway, rheumatoid arthritis, lipid and atherosclerosis, and Th17 cell differentiation (**Figure 6B**).

For large and small AAA samples (**Figure 6C**), DEGs were enriched in biological processes, such as vascular wound healing and angiogenesis involved in wound healing; cell components, such as specific granules, tertiary granules, and secretory granule membranes; and molecular functions, such as hydrolase activity and hydrolyzing O-glycosyl. The KEGG pathway was enriched for necroptosis, glycosaminoglycan degradation, and SNARE interactions in vesicular transport (**Figure 6D**).

### Identification of *G0S2* in AAA

**Figure 7:**
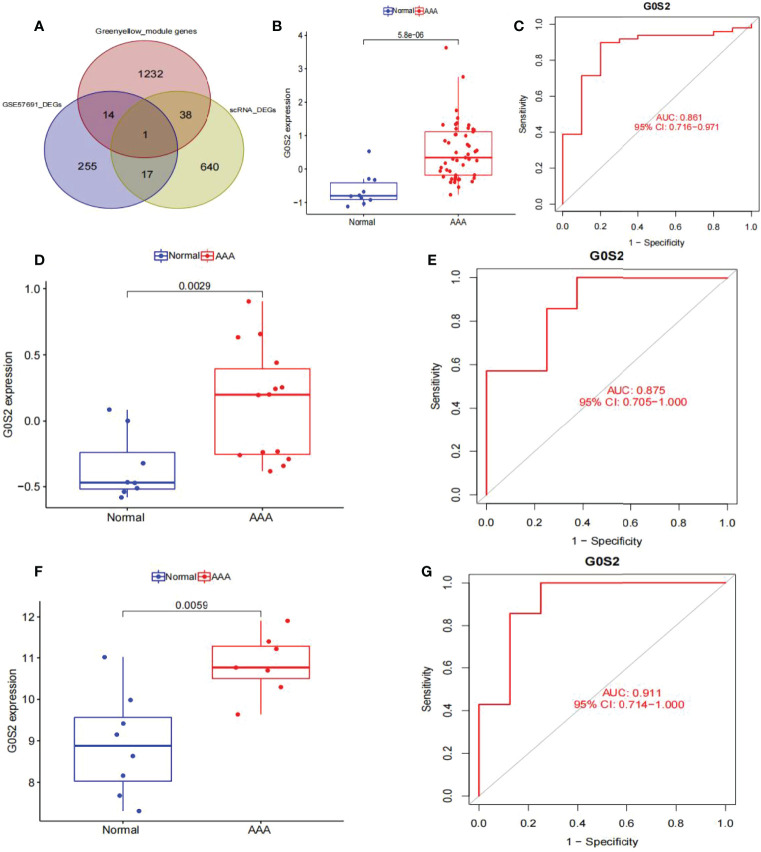
Obtaining the key gene of AAA: *G0S2*, by single-cell RNA-sequencing analysis, weighted co-expression network analysis, and differential expression analysis. **(A)** Intersection of single-cell analysis, WGCNA and differential expression analysis was displayed in a Venn diagram. The most significant gene, *G0S2*, was obtained. **(B)** *G0S2* mRNA expression in AAA compared to normal samples in the GSE57691. **(C)** ROC curve construction in public data sets to evaluate the diagnostic accuracy of *G0S2* of AAA. The AUC of GSE57691 was 0.861. **(D)** *G0S2* mRNA expression in AAA compared to normal samples in the GSE47472. **(E)** ROC curve construction in public data sets to evaluate the diagnostic accuracy of *G0S2* of AAA. The AUC of GSE47472 was 0.875. **(F)** *G0S2* mRNA expression in AAA compared to normal samples in the GSE7284. **(G)** ROC curve construction in public data sets to evaluate the diagnostic accuracy of *G0S2* of AAA. The AUC of GSE7284 was 0.911. The distinction was considered good when the AUC value was between 0.8 and 0.9, and exceptional when the AUC value was > 0.9. ROC, receiver operating characteristic; AUC, area under the ROC curve; AAA, abdominal aortic aneurysm.

Venn graphs were used to aggregate the DEGs identified through single-cell and WGCNA analyses throughout the dataset (**Figure 7A**). A key gene, *G0S2*, was identified at this intersection, suggesting that this gene may be involved in AAA development. Using box plots, we found that *G0S2* was highly upregulated in AAA samples from GSE57691 and GSE47472 (**Figures 7B, D**). Subsequently, we constructed ROC curves of the two chip datasets and discovered that the AUC of datasets GSE57691 (**Figure 7C**) and GSE47472 (**Figure 7E**) were 0.861 and 0.875, respectively; thereby suggesting that *G0S2* may be an effective diagnostic biomarker of AAA. To confirm previous findings, we created a box plot of GSE7284 and observed that *G0S2* was considerably upregulated in AAA samples (**Figure 7F**). Additionally, ROC analyses were conducted for the GSE7284 dataset and produced an AUC of 0.911 (**Figure 7G**). Although the small sample size may have influenced the ROC value, the results indicate that this gene has a positive effect on the diagnosis of AAA and normal samples.

### Identification of Biomarkers in Dilated-AAA

**Figure 8:**
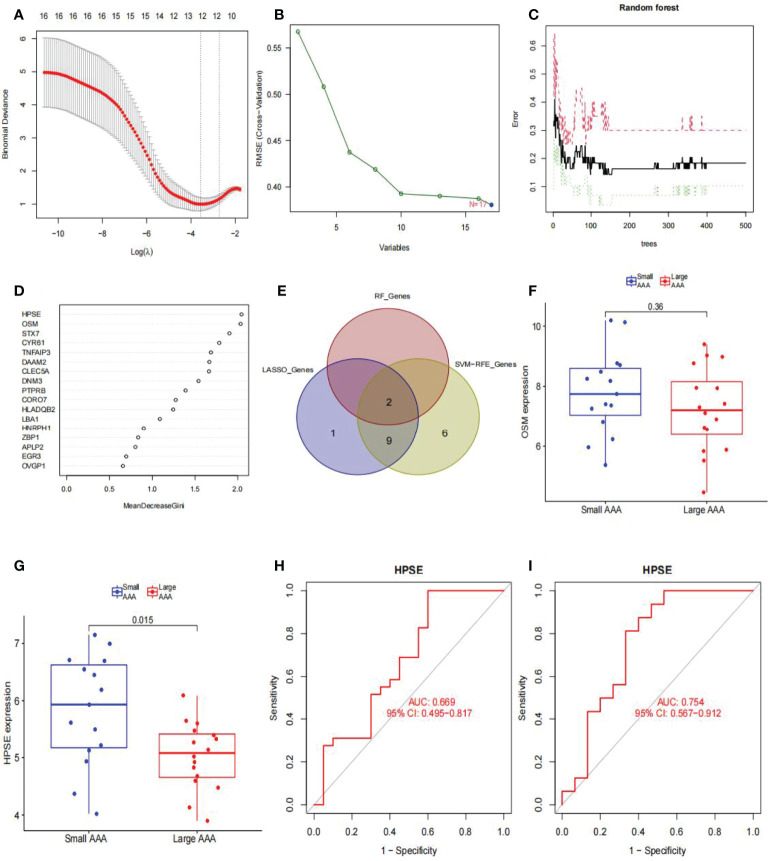
Diagnostic indicators for large AAA screening and validation. **(A)** Fine-tuning the least absolute shrinkage and selection operator (LASSO) model’s feature selection. LASSO regression was used to narrow down the DEGs, resulting in the discovery of 12 variables as potential markers for AAA. The ordinate represents the value of the coefficient, the lower abscissa represents log (λ), and the upper abscissa represents the current number of non-zero coefficients in the model. **(B)** A plot illustrating the process of selecting biomarkers using the support vector machine-recursive feature elimination (SVM-RFE) technique. The SVM-RFE technique was used to identify a subset of 17 characteristics from the DEGs. **(C)** The effect of the decision tree number on the error rate. The x-axis denotes the number of decision trees, while the y-axis shows the error rate. When approximately 200 decision trees are used, the error rate is generally steady. **(D)** The Gini coefficient method’s results in a random forest classifier. The x-axis displays the genetic variable, and the y-axis the significance index. **(E)** Venn diagram showing overlapping markers. **(F)** *OSM* mRNA expression in large AAA samples is not statistically significant (P > 0.05) when compared to small AAA samples in the GSE98278. **(G)** *HPSE* mRNA expression is significantly higher in large AAA samples than in small AAA samples in the GSE98278 (P < 0.05). ROC curves were constructed using publicly available data to assess the diagnostic accuracy of *HPSE* for large AAA. **(H)** GSE57691 has an AUC of 0.669. **(I)** GSE98278 had an AUC of 0.754. ROC, receiver operating characteristic; AUC, area under the ROC curve; AAA, abdominal aortic aneurysm.

We used LASSO logistic regression to identify 12 critical biomarkers from the DEGs (**Figures 8A, B**). The SVM-RFE method identified 17 genes as important biomarkers for DEGs (**Figure 8B**). In addition, the RF algorithm identified two genes as key indicators (**Figures 8C, D**). The three methods identified overlapping genes, one upregulated (*OSM*), one downregulated (*HPSE*) (**Figure 8E**). Box plots were used to visualize the data from one chip, and we observed that *HPSE* was highly upregulated in small AAA samples in GSE98278 (**Figures 8F, G**) with an AUC of 0.669 in the GSE57691 (**Figure 8H**) and 0.754 in the GSE98278 dataset (**Figure 8I**). While the small sample size may have an influence on the AUC values calculated here, the data indicate that this gene is also effective in the differentia diagnosis of small and large AAA samples.

### Infiltration of Immune Cells Results

**Figure 9:**
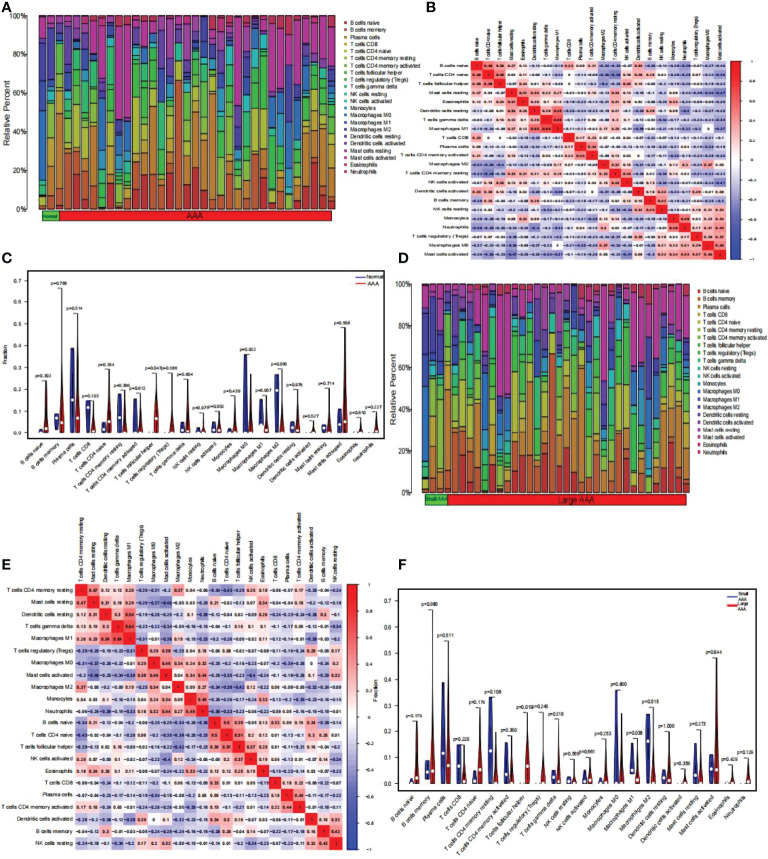
The composition of immune cells was analyzed and displayed. **(A)** Heat map of the 22 immune cell subpopulations comparing AAA and normal samples. **(B)** Heat map showing the correlation between 22 different kinds of immune cells in AAA and normal samples. The size of the colored squares indicates the connection’s strength; red indicates a positive correlation, while blue indicates a negative correlation. The stronger the connection, the redder the hue. **(C)** Violin diagram illustrating the proportion of 22 different kinds of immune cells in AAA versus normal samples. (Normal samples were denoted by blue color, whereas AAA samples were denoted by red color. AAA, abdominal aortic aneurysm. P-values < 0.05 were considered as statistically significant). **(D)** Heat map of the 22 immune cell subpopulations comparing large AAA and small AAA samples. **(E)** Correlation heat map between large AAA and small AAA samples of 22 kinds of immune cells. The size of the colored squares indicates the connection’s strength; red indicates a positive correlation, while blue indicates a negative correlation. The stronger the connection, the redder the hue. **(F)** Violin diagram illustrating the proportion of 22 different kinds of immune cells in large and small AAA samples. (The small AAA samples were marked with blue color and large AAA samples were marked with red color. AAA, abdominal aortic aneurysm. P-values < 0.05 were considered as statistically significant).

Using the CIBERSORT algorithm, we first summarized the results obtained from 10 normal and 49 AAA samples (**Figure 9A**). As indicated by the correlation heatmap of the 22 immune cells (**Figure 9B**) T follicular helper (Tfh) cells and eosinophils (r = 0.24), resting dendritic cells (r = 0.01), gamma delta T cells (r = 0.15), activated NK cells (r = 0.35), activated dendritic cells (r = 0.15), memory B cells (r = 0.25), naïve CD4 T cells (r = 0.49), and naïve B cells (r = 0.38) displayed significant positive correlations. Conversely, significant negative correlations was verified with resting mast cells (r = -0.07), M1 macrophages (r = -0.08), plasma cells (r = -0.12), activated memory CD4 T cells (r = -0.2), M2 macrophages (r = -0.4), resting memory CD4 T cells (r = -0.25), memory B cells (r = -0.05), resting NK cells (r = -0.2), monocytes (r = -0.19), neutrophils (r = -0.26), regulatory T cells (Tregs) (r = -0.03), M0 macrophages (r = -0.18), activated mast cells (r = -0.23). AAA samples generally contained a higher proportion of Tfh cells than normal samples (p < 0.05) (**Figure 9C**). Additionally, we summarized the results obtained from 20 small and 29 large AAA samples (**Figure 9D**). As indicated by the correlation heatmap of the 22 immune cells (**Figure 9E**), M1 macrophages and resting CD4 memory T cells (r = 0.26), resting mast cells (r = 0.29), resting dendritic cells (r = 0.54), gamma delta T cells (r = 0.64), M2 macrophages (r = 0.19), and eosinophils (r = 0.01) displayed significant positive correlations, while significant negative correlations were verified with Tregs (r = -0.31), M0 macrophages (r = -0.01), activated mast cells (r = -0.38), monocytes (r = -0.15), neutrophils (r=-0.33), naïve B cells (r = -0.2), naïve CD4 T cells (r = -0.26), Tfh cells (r = -0.09), activated NK cells (r = -0.02), CD8 T cells (r = -0.12), plasma cells (r = -0.14), activated CD4 memory T cells (r = -0.01), activated dendritic cells (r = -0.38), memory B cells (r = -0.03), and resting NK cells (r = -0.2). M2 macrophages and resting CD4 memory T cells (r = 0.37), M0 macrophages (r = 0.34), activated mast cells (r = 0.04), monocytes (r = 0.09), neutrophils (r = 0.27), NK cells (r = 0.12), and activated CD8 T cells (r = 0.06) displayed significant positive correlations, while significant negative correlations with resting mast cells (r = -0.05), resting dendritic cells (r = -0.2), gamma delta T cells (r = -0.09), Tregs (r=-0.25), naïve B cells (r = -0.34), naïve CD4 T cells (r = -0.38), Tfh cells (r = -0.42), eosinophils (r = -0.22), plasma cells (r = -0.08), activated CD4 memory T cells (r = -0.05), activated dendritic cells (r = -0.32), memory B cells (r = -0.24), resting NK cells (r = -0.09); Tfh cells and resting dendritic cells (r = 0.02), gamma delta T cells (r = 0.16), naïve B cells (r = 0.39), naïve CD4 T cells (r = 0.51), activated NK cells (r = 0.37), eosinophils (r = 0.25), CD8 T cells (r = 0.01), and activated dendritic cells (r = 0.16) displayed significant positive correlations, while significant negative correlations was verified with resting CD4 memory T cells (r = -0.29), resting mast cells (r = -0.13), Tregs (r=-0.02), M0 macrophages (r = -0.16), activated mast cells (r = -0.22), monocytes (r = -0.17), neutrophils (r = -0.23), plasma cells (r = -0.11), activated CD4 memory T cells (r = -0.21), memory B cells (r = -0.04), resting NK cells (r = -0.2). Generally, AAA samples had a higher proportion of Tfh cells (P < 0.05), but relatively lower proportions of M1 and M2 macrophages (P < 0.05) than normal samples did (**Figure 9F**).

### Biomarkers and Immune Cells

**Figure 10:**
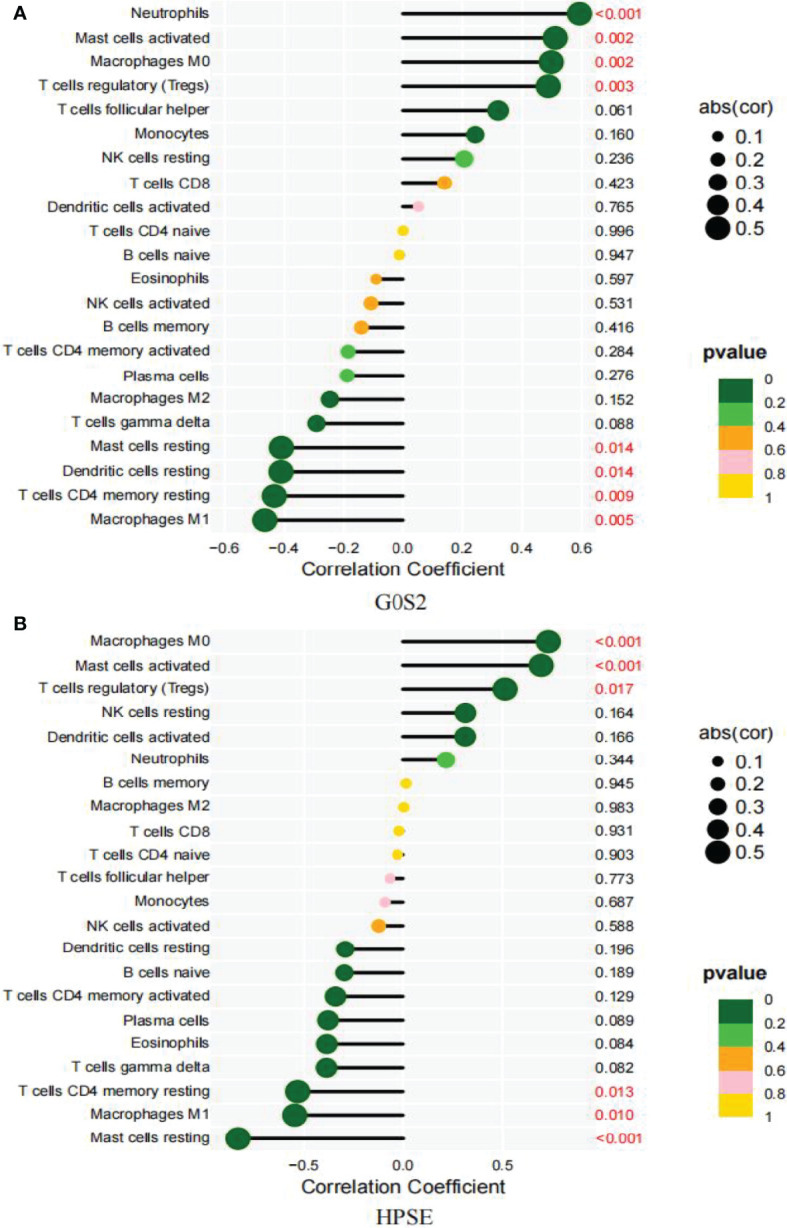
Correlation between diagnostic markers and infiltrating immune cells. **(A)** Correlation between G0S2 and infiltrating immune cells. **(B)** Correlation between HPSE and infiltrating immune cells. The size of the dots indicates the degree to which genes and immune cells are correlated. Correlation strength is proportional to the size of the dots. The color of the dots indicates the P-value; a yellower hue indicates a lower P-value, while a greener color indicates a higher P-value. P-value < 0.05 was considered statistically significant.

Based on the results of the correlation analysis between AAA and normal samples, G0S2 displayed a positive correlation with neutrophils (r = 0.59, P = 0.00018; **Supplementary Figure 1A**), activated mast cells (r = 0.51, P = 0.0017; **Supplementary Figure 1B**), M0 macrophages (r = 0.5, P = 0.0023; **Supplementary Figure 1C**), and Tregs (r = 0.49, P = 0.003; **Supplementary Figure 1D**), but it showed a negative correlation with resting mast (r = -0.41, P = 0.014; **Supplementary Figure 1E**) and dendritic cells (-0.41, P = 0.014; **Supplementary Figure 1F**), resting CD4 memory T cells (r = -0.43, P = 0.0093; **Supplementary Figure 1G**) and M1 macrophages (r = -0.47, p = 0.0048; **Supplementary Figure 1H**) (**Figure 10A**).

Between the small and large AAA samples, HPSE showed a positive correlation with M0 macrophages (r = 0.73, P = 0.00016; **Supplementary Figure 2A**), activated mast cells (r = 0.7, P = 0.00046; **Supplementary Figure 2B**), and Tregs (r = 0.51, P = 0.017; **Supplementary Figure 2C**), but it showed a negative correlation with M1 macrophages (r = -0.55, P = 0.01; **Supplementary Figure 2D**), resting mast cells (r = -0.83, P = 2.9e-06; **Supplementary Figure 2E**), and resting dendritic cells (r =-0.53, p = 0.013; **Supplementary Figure 2F**) (**Figure 10B**).

### Verifification of Diagnostic Markers

**Figure 11:**
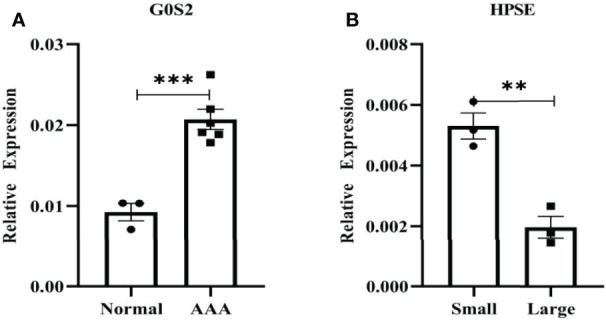
Verifification of G0S2 and HPSE by qRT-PCR. **(A)** G0S2 showed the signifificant upregulation in AAA tissues. **(B)** HPSE showed a signifificant upregulation in small AAA samples. **P < 0.01, ***P < 0.001.

**Figure 11** shows the expression levels of two biomarkers detected by qRT-PCR in 9 tissue samples (3 normal samples, 3 small AAA samples and 3 large AAA samples). G0S2 showed the signifificant upregulation in AAA tissues (P < 0.001) (**Figures 11A**), and HPSE showed a signifificant upregulation in small AAA samples (P < 0.01) (**Figures 11B**), indicating that the results were reproducible and reliable.

## Discussion

AAAs have a significant impact on quality of life and create a high financial burden on families. Despite advancements in surgical procedures for AAA therapy, postoperative morbidity and mortality remain prevalent. Therefore, it is critical to better understand the pathophysiology and progression of AAAs, and thereby identify novel diagnostic biomarkers and therapeutic targets.

In this study, we identified a gene, *G0S2*, that was strongly related to AAA. This was performed using single-cell, weighted co-expression network, and differential expression analyses of an existing AAA dataset. A ROC curve analysis then confirmed that *G0S2* was capable of accurately diagnosing AAA and our findings suggest that its increased expression may be associated with Tfh cells. Additionally, the results from the follow-up of patients with AAA in the UK Multicenter Aneurysm Screening Study (MASS) demonstrated that AAA with a diameter > 5.5 cm was dangerous and was linked with a very high incidence of rupture (23). To uncover additional molecular pathways and risk genes associated with AAA progression, we analyzed GSE57691, which included critical clinical data, such as the size of the AAA, and GSE98278, which served as a validation data set. We then used three machine learning algorithms, each possessing unique attributes, to screen for risk genes. Finally, *HPSE* and *OSM* were selected, and *HPSE* was found to be effective for in-depth verification, indicating the feasibility of the integration strategy. Additionally, its upregulation was believed to be associated with Tfh cells and M1 macrophages.

Cell proliferation, apoptosis, inflammation, metabolism, and carcinogenesis are all regulated by the G0/G1 switch gene 2 (*G0S2*) (24). Initially, Russell et al. found that *G0S2* was differentially expressed in lymphocytes during the lectin-induced transition from the G0 to G1 phase of the cell cycle (25). G0S2 encodes a 103-amino acid protein that shares 78% of its sequence with humans; its mRNA is strongly expressed in brown and white adipose tissues and is related to growth arrest in 3T3-L1 fibroblasts (26). Yang et al. (27) described the localization of *G0S2* in lipid droplets. It inhibits adipocyte triglyceride lipase (ATGL) and triglyceride hydrolase activity and plays a critical role in controlling lipolysis in adipocytes. Other studies show that it also leads to liver steatosis (28, 29). According to a previous study (30), *G0S2* interacts with Bcl-2 and promotes apoptosis in tumor cells. Additionally, Kioka et al. (31) demonstrated that *G0S2* prevents ATP depletion in cells and causes hypoxia tolerance. However, its roles and functions in AAA remain unknown. Previously, only one study has reported the role of *G0S2* in coronary artery atherosclerosis. Knapp et al. (32) found that coronary atherosclerosis raises only *G0S2* and *FABP4* transcript levels in the myocardium. Most significantly, adipose triacylglyceride lipase (ATGL), β-HAD, and COX4/1 protein expressions were decreased in the CAD group, which was associated with more than a doubling of the triglycerides content. The production and uptake of fatty acids are stable in the myocardium of patients with coronary artery disease. Additionally, patients with coronary stenosis have a high expression of pro-inflammatory proteins in their myocardium. Here, we found that *G0S2* was increased in the perivascular adipose tissue, though FABP-4 protein levels were increased and COX4/1 protein content was decreased. These findings imply that a reduction in ATGL protein expression results in myocardial steatosis in patients with coronary artery disease. As a result, *GOS2* may play a role in the development of AAA similar to that in coronary atherosclerotic disease, and future experiments are required to investigate this possibility.Kram et al. (33) demonstrated that transgenic mice overexpressing human *HPSE* (34) had enhanced trabecular bone mass and bone formation rates. Exogenous *HPSE* was shown to have a pro-osteogenic effect *in vitro*, stimulating the osteogenic differentiation of cultivated MC3T3 E1 osteoblastic cells. Manton et al. (35) found that long-term treatment of human mesenchymal stem cell (*MSCs*) with heparan sulfate (HS)- and chondroitin sulfate (CS)-degrading enzymes increased osteogenic differentiation, which they attributed to altered bone morphogenetic protein (*BMP*) and *Wnt* activity resulting from disrupted cell surface proteoglycan expression. Additionally, *HPSE* has been shown to inhibit osteoblastogenesis and bone formation, implying that this protein may play a significant role in pathological bone remodeling (36). Aldi and colleagues (37) suggested that *HSPE* may have a dual function in vascular calcification, depending on the stage of the illness and the presence of inflammatory cells. The mineralization and osteogenic differentiation of vascular smooth muscle cells have been shown to be improved by *HPSE*; however, it has been associated to inflammation-induced osteoclast growth and activity in advanced atherosclerotic plaques. According to the AAA formation theory, alterations in the aortic wall caused by atherosclerosis underlie AAA pathogenesis; therefore, inflammatory pathways that promote atherosclerosis also contribute to AAA (38). Therefore, the mechanism underlying the higher risk of rupture in AAA > 5.5 cm may also be related to previous findings, but additional validation is required. These two genes are involved in the pathophysiology of atherosclerotic disease, confirming the inseparable link between AAA, atherosclerosis, and inflammation. We therefore identified a feasible intervention target for improving the prognosis of AAA.

We used CIBERSORT to compare AAA and normal samples in GSE57691 and discovered that Tfh cells were considerably overexpressed in AAA samples, implying that they play a key role in AAA. Tfh cells, which express the defining transcription factor B cell lymphoma 6 (BCL6), are found in B cell follicles, in which they coexist and maintain and form germinal centers. They are required for antibody isotype switching in germinal center B cells (39) and have been identified as a CD4+ subset that specializes in assisting B cells in secondary lymphoid organ (*SLOs*) germinal centers. They play a vital role in the pathophysiology of various diseases, including autoimmune disorders, allergies, infectious diseases, and cancers (40–43). Recent studies have shown that Tfh cells play a critical role in arteriosclerosis and are possibly pro-atherogenic. In atherosclerosis-prone mice, the atherogenic environment enhanced the autoimmune responses of CXCR3+ Tfh cells (44). Additionally, inhibiting inducible T-cell co-stimulator and its ligand signaling in Apoe–/–mice reduced the burden of atherosclerosis, as seen by decreased Tfh cell numbers in secondary lymphoid organs. Aging increased the proportion of Tfh cells in Apoe–/– mice, but not in wild-type mice, although aging had no effect on the overall percentage of CD4+ T cells in Apoe–/– animals (45). Interestingly, Tfh cells can be derived from Tregs and their reduction in Apoe–/– mice reduces atherosclerosis (45). Additionally, Wang et al. (46) demonstrated that homocysteine is involved in the imbalance of Tfh and Th17 cells by upregulating AIM2 and NLRP1 inflammasomes, which are related to AAA. Based on these findings, we hypothesized that Tfh cells performed a similar role in atherosclerotic diseases, and thus indirectly contributed to AAA development.

Additionally, Tfh cells may contribute directly to AAA *via* inflammation-related mechanisms. However, the inflammation-mediated theory of AAA has been demonstrated to encompass a diverse variety of innate and adaptive immune cells and their products within the aortic wall and intraluminal thrombi in human AAA samples (45, 47–49). Quantification of hematopoietic cells from human AAA wall biopsies revealed that approximately 50% were T cells, 40% B cells, 7% NK cells, and 2% monocytes (50), which was similar to the findings of our single-cell analysis, indicating that these large members comprised the AAA immunomodulatory network and contributed to AAA development (50). Thus, our single-cell analysis results more accurately reflected the substantial variations in cellular composition between normal and AAA samples. Recent studies have established the mechanisms of action of T cells (51–54), mast cells (55), B cells (56), monocytes (57), NK cells (58), and macrophages (59).

We used CIBERSORT to examine the differences in immune cell infiltration between small and large AAAs, and we observed that Tfh cells were strongly expressed in large AAAs, whereas M1 and M2 macrophages were strongly expressed in small AAAs. M1 macrophages have been demonstrated to aggravate local inflammation as well as enhance aortic dilatation and vascular remodeling, while M2 macrophages are often produced by Th2 cytokines, such as IL-4 and IL-13 (60, 61). M2 macrophages have the ability to regulate angiogenesis, cell recruitment, and collagen deposition *via* the mobilization of mast and NK cells (62). Aorta walls become more dominant in M2 macrophages as the disease progresses, suggesting a compensatory mechanism to compensate for the anti-inflammatory and tissue repair actions of M2 macrophages (57). In a study conducted by Cheng et al. (63), Apoe -/- mice with AAA were given notch receptor inhibitors that raised M2 macrophages and decreased M1 macrophages. They discovered that this intervention improved the development of AAA (63). As a result, the counteracting effects of M1 and M2 macrophages in small AAAs make them eligible as therapeutic targets to control inflammation and the destruction of aortic walls and decrease rupture due to AAA expansion.

Thus, we used a combination of single-cell, WGCNA, and differential expression analyses to reveal the genetic variations between AAAs. We identified *G0S2* as a highly accurate biomarker for the effective diagnosis of AAA enabling early treatment. Additionally, we identified a link between the risk gene *HPSE* and large AAAs, which may serve as a therapeutic target for delaying AAA dilatation. We employed additional datasets to confirm the expression of *G0S2* and *HPSE* as well as the ROC curve, but functional studies will be conducted in future works. However, our research has some limitations due to small sample sizes and a lack of informative sample data: (1) disease assessment and prediction accuracy can be improved by increasing the sample size; (2) the potential marker genes and pathways identified in this study need to be further validated to provide actual evidence for clinically targeted therapies; (3) analyses of the marker genes’ protein expression level could provide substantial evidence. However, due to a shortage of appropriate normal abdominal aorta samples in our department, executing the verification experiment is problematic. We intend to collect abdominal aorta tissue in the future in order to further understanding of how *G0S2* and *HPSE* influence AAA.

## Conclusion

Through single-cell, WGCNA, differential expression analyses and combining multiple machine learning methods, we identified *G0S2* as a novel AAA biomarker and *HPSE* as a protective biomarker for large AAA. The two biomarkers were verified using additional GEO data. Moreover, immune infiltration analysis revealed that Tfh cells play an important role in AAA progression. Hence, our findings may represent a new reference point for diagnosing and treating AAA and delaying AAA dilatation in patients in the future.

## Data Availability Statement

The datasets presented in this study can be found in online repositories. The names of the repository/repositories and accession number(s) can be found in the article/**Supplementary Material**.

## Ethics Statement

The Shanxi Provincial People's Hospital Ethical Committee reviewed and approve the research involving human participants. The patients/participants provided their written informed consent to participate in this study.

## Author Contributions

TX was responsible for study conception; XSL, GJW and YXG contributed to the methodology; CL, XFH and JKW provided software; TX wrote the original draft; YFF contributed to the data analysis and supervised the execution of codes; FQL contributed to reviewing. All authors reviewed and approved the final version of the work.

## Funding

This study was supported bythe Key Basic Natural Science Foundation of Shaanxi Province (No. 2022JZ-47), Key Industrial Innovation Chain Project in Shaanxi Province of China (grant no. 2021ZDLSF02-03), Science and Technology Program of Xi’an (grant no. 21YXYJ0095), Natural Science Foundation of Shaanxi Province (No. 2022SF-476 and No. 2021SF-329), and the Open Program of Shaanxi Key Laboratory of Integrative Traditional and Western Medicine for Prevention and Treatment of Cardiovascular Diseases, Shaanxi University of Chinese Medicine (grant no. 2022XXG-ZD-001).

## Conflict of Interest

The authors declare that the research was conducted in the absence of any commercial or financial relationships that could be construed as a potential conflict of interest.

## Publisher’s Note

All claims expressed in this article are solely those of the authors and do not necessarily represent those of their affiliated organizations, or those of the publisher, the editors and the reviewers. Any product that may be evaluated in this article, or claim that may be made by its manufacturer, is not guaranteed or endorsed by the publisher.

## Supplementary Material

Supplementary Figure 1 Visualization of immune cell infiltration and *G0S2* correlation analysis results based on GSE57691. **(A)** Correlation analysis between the expression of *G0S2* and neutrophils. **(B)** Correlation between *G0S2* and activated mast cells. **(C)** Correlation analysis of *G0S2* and M0 macrophage expression. **(D)** Correlation analysis between the expression of *G0S2* and regulatory T cells (Tregs). **(E)** Correlation analysis between the expression of *G0S2* and resting mast cells. **(F)** Correlation analysis in the expression of *G0S2* and resting dendritic cells. **(G)** Correlation analysis of the expression of *G0S2* and resting CD4 memory T cells. **(H)** Correlation between *G0S2* and M1 macrophage expression.

Supplementary Figure 2 Visualization of the results of immune cell infiltration and HSPE correlation analysis based on GSE57691. **(A)** Correlation analysis of *G0S2* and M0 macrophage expression. **(B)** Correlation analysis in the expression of *G0S2* and activated mast cells. **(C)** Correlation analysis between the expression of *G0S2* and regulatory T cells (Tregs). **(D)** Correlation analysis of the expression of *G0S2* and resting memory CD4 T cells. **(E)** Correlation analysis of *G0S2* and M1 macrophage expression. **(F)** Correlation between *G0S2* and resting mast cells.

Supplementary Table 1 “SingleR” package was used to annotate the cells and produce 20 clusters.

Supplementary Table 2 The “FindMarkers” method was used to identify genes that differed significantly between AAA and normal cells.

Supplementary Table 3 All modular genes obtained from WGCNA.

Supplementary Table 4 DEGs between AAA and normal samples.

Supplementary Table 5 DEGs between large AAA and small AAA samples.

Supplementary Table 6 Results of immune cell infiltration between AAA and normal samples.

Supplementary Table 7 Results of immune cell infiltration between large and small AAA samples.

## Abbreviations

AAA, abdominal aortic aneurysm; WGCNA, weighted co-expression network analysis; DEGs, differentially expressed genes; G0S2, G0/G1 switch 2; HPSE, heparinase; Tregs, regulatory T cells; PCs, principle components; scRNA-seq, single-cell RNA-seq; GO, Gene Ontology; KEGG, Kyoto Encyclopedia of Genes and Genomes; ROC, receiver operating characteristic; AUC, area under the curve; RF, random forests; LASSO, least absolute shrinkage and selection operator; SVM-RFE, support vector machine-recursive feature elimination; MSC, Mesenchymal Stem; CaMKII, Ca2+/calmodulin-dependent protein kinase II; OSM, oncostatin M; Tfh, T follicular helper; MASS, Multicenter Aneurysm Screening Study; ATGL, adipocyte triglyceride lipase; AIM2, absent in melanoma 2; NLRP1, NLR family pyrin domain containing 1; SLOs, secondary lymphoid organ germinal centers; MSCs, human mesenchymal stem cell; BMP, bone morphogenetic protein; ATGL, adipose triacylglyceride lipase.
