# Supplementary figures and images for: Single-Cell Sequencing Analysis and Multiple Machine Learning Methods Identified G0S2 and HPSE as Novel Biomarkers for Abdominal Aortic Aneurysm
Source: Front Immunol. 2022 Jun 13;13:907309. doi: 10.3389/fimmu.2022.907309 (PMC9234288; doi:10.3389/fimmu.2022.907309)

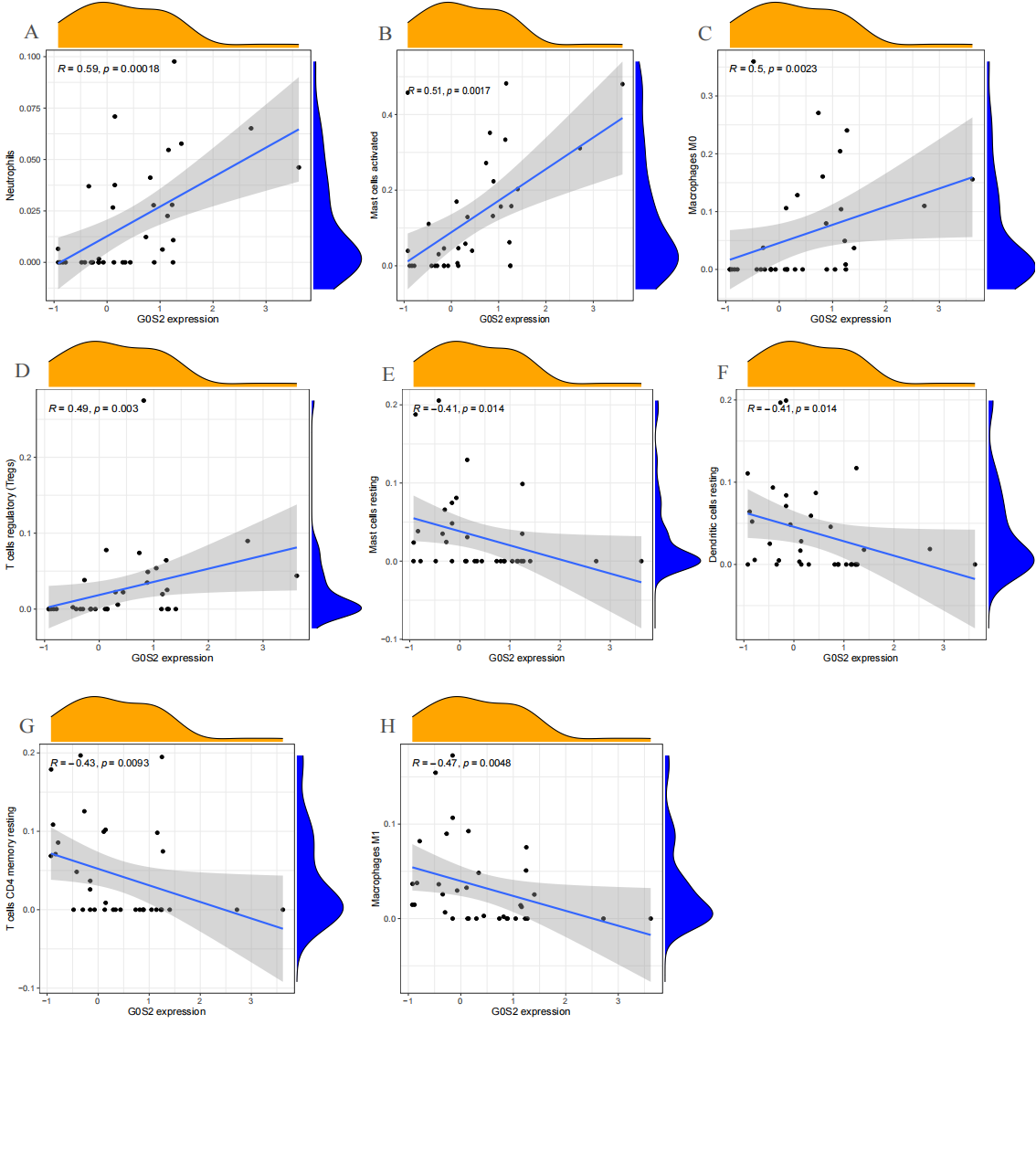

Supplement: Supplementary Figure 1 — Visualization of immune cell infiltration and G0S2 correlation analysis results based on GSE57691. (A) Correlation analysis between the expression of G0S2 and neutrophils. (B) Correlation between G0S2 and activated mast cells. (C) Correlation analysis of G0S2 and M0 macrophage expression. (D) Correlation analysis between the expression of G0S2 and regulatory T cells (Tregs). (E) Correlation analysis between the expression of G0S2 and resting mast cells. (F) Correlation analysis in the expression of G0S2 and resting dendritic cells. (G) Correlation analysis of the expression of G0S2 and resting CD4 memory T cells. (H) Correlation between G0S2 and M1 macrophage expression. [file Image_1.tif]

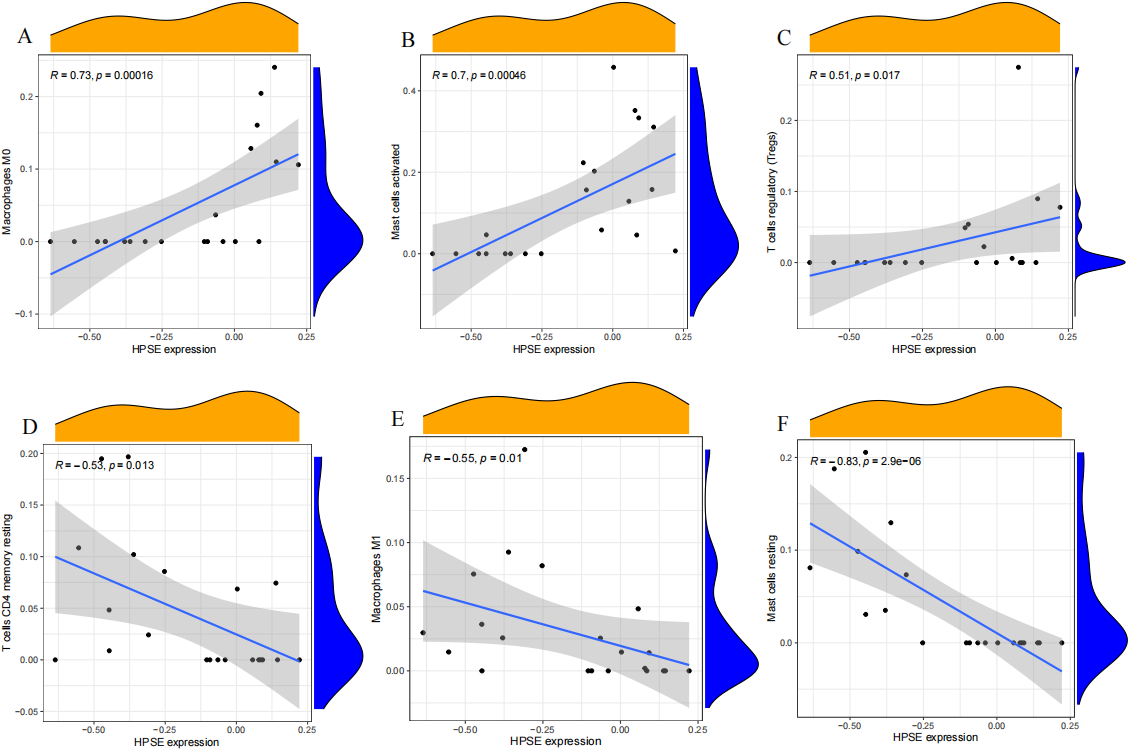

Supplement: Supplementary Figure 2 — Visualization of the results of immune cell infiltration and HSPE correlation analysis based on GSE57691. (A) Correlation analysis of G0S2 and M0 macrophage expression. (B) Correlation analysis in the expression of G0S2 and activated mast cells. (C) Correlation analysis between the expression of G0S2 and regulatory T cells (Tregs). (D) Correlation analysis of the expression of G0S2 and resting memory CD4 T cells. (E) Correlation analysis of G0S2 and M1 macrophage expression. (F) Correlation between G0S2 and resting mast cells. [file Image_2.tif]
