# Supplementary material for: Single-Cell Sequencing Analysis and Multiple Machine Learning Methods Identified G0S2 and HPSE as Novel Biomarkers for Abdominal Aortic Aneurysm
Source: Front Immunol. 2022 Jun 13;13:907309. doi: 10.3389/fimmu.2022.907309 (PMC9234288; doi:10.3389/fimmu.2022.907309)
Supplement: Supplementary Table 1 — “SingleR” package was used to annotate the cells and produce 20 clusters. [file Table_1.doc]

| Cluster ID | Celltype |
| --- | --- |
| 0 | T_cells |
| 1 | Epithelial_cells |
| 2 | B_cell |
| 3 | Monocyte |
| 4 | Epithelial_cells |
| 5 | T_cells |
| 6 | MSC |
| 7 | Monocyte |
| 8 | Macrophage |
| 9 | T_cells |
| 10 | B_cell |
| 11 | Monocyte |
| 12 | NK_cell |
| 13 | Epithelial_cells |
| 14 | Fibroblasts |
| 15 | Endothelial_cells |
| 16 | MSC |
| 17 | Epithelial_cells |
| 18 | Monocyte |
| 19 | Keratinocytes |

**Supplementary table 1**
