# Supplementary material for: Single-Cell Sequencing Analysis and Multiple Machine Learning Methods Identified G0S2 and HPSE as Novel Biomarkers for Abdominal Aortic Aneurysm
Source: Front Immunol. 2022 Jun 13;13:907309. doi: 10.3389/fimmu.2022.907309 (PMC9234288; doi:10.3389/fimmu.2022.907309)
Supplement: Supplementary Table 2 — The “FindMarkers” method was used to identify genes that differed significantly between AAA and normal cells. [file Table_2.doc]

**Supplementary table 2**

| HBB |
| --- |
| KLF6 |
| SOD2 |
| HBA2 |
| PLAUR |
| IL8 |
| CXCL3 |
| CXCL2 |
| ICAM1 |
| CCL3 |
| HLA-DRB5 |
| PPP1R15A |
| IL1B |
| MARCKS |
| NINJ1 |
| CCL20 |
| S100A9 |
| BASP1 |
| SAT1 |
| HLA-DQA1 |
| TXN |
| TNFAIP3 |
| G0S2 |
| CSTB |
| PNRC1 |
| CCL4 |
| S100A8 |
| CFD |
| SMDT1 |
| RAB11FIP1 |
| THBS1 |
| C15orf48 |
| SUB1 |
| FCGR2A |
| CXCL1 |
| IGLL5 |
| MMP19 |
| IL7R |
| ETS2 |
| CFLAR |
| TPI1 |
| PTGS2 |
| HCST |
| MS4A7 |
| TPM4 |
| CCNL1 |
| FTH1 |
| SLC2A3 |
| PLEK |
| TREM1 |
| ATP6V1F |
| TGFBI |
| NFKBIA |
| CXCL5 |
| EREG |
| RPS4Y1 |
| RPL22L1 |
| ZFP36L2 |
| STAB1 |
| CTSL |
| IL1RN |
| LGALS3 |
| JUND |
| SPAG9 |
| TMEM176B |
| SAMSN1 |
| CMTM6 |
| EPB41L3 |
| CLEC12A |
| CECR1 |
| LGALS2 |
| POMP |
| HLA-DMA |
| MT-ND4L |
| DUSP6 |
| HLA-DRA |
| MT-CYB |
| CIB1 |
| INHBA |
| FTL |
| HBA1 |
| HLA-DPB1 |
| FAM118A |
| PDXK |
| CTSB |
| RPS27 |
| TIMP1 |
| CD83 |
| IER3 |
| CALM1 |
| RAB7A |
| NFKB1 |
| PPIF |
| LITAF |
| NLRP3 |
| IL6 |
| RSRC2 |
| ZFP36L1 |
| RP11-701P16.5 |
| SULT1A1 |
| ATP6V1B2 |
| CD81 |
| UBB |
| ABL2 |
| MT-ND3 |
| FOSB |
| BNIP3L |
| TNIP1 |
| TMEM50A |
| TNFAIP8 |
| PMP22 |
| CASP4 |
| ITGAX |
| RNF144B |
| ANXA5 |
| MALAT1 |
| IVNS1ABP |
| GADD45GIP1 |
| MXD1 |
| SH3BP5 |
| PID1 |
| TNFRSF14 |
| UPP1 |
| WAC |
| LGALS1 |
| RPS29 |
| DNAJB6 |
| PIM3 |
| NAMPT |
| FOLR2 |
| CREM |
| BCL2A1 |
| CCL3L1 |
| MAP2K3 |
| PLA2G7 |
| PEBP1 |
| LINC00152 |
| F13A1 |
| C1orf122 |
| HSP90AB1 |
| CD59 |
| PLD3 |
| ISG15 |
| SQSTM1 |
| METRNL |
| ANKRD13D |
| CD74 |
| CYSTM1 |
| SOCS3 |
| C1QC |
| IL1A |
| PLEKHB2 |
| CD44 |
| PDE4B |
| DUSP2 |
| CLEC2B |
| MT2A |
| CYTH4 |
| SERPINB9 |
| C1QA |
| EHD1 |
| NFKBIZ |
| GK |
| CAMKK2 |
| NBN |
| TMEM176A |
| MT-ATP6 |
| ATP1B3 |
| ATP13A3 |
| NFKB2 |
| TLR2 |
| TSPAN4 |
| ZFP36 |
| RAB13 |
| SNX8 |
| LMNA |
| HSPE1 |
| TCEB1 |
| RHOH |
| TANK |
| HLA-DPA1 |
| PLK3 |
| MFSD1 |
| SIPA1L1 |
| EIF4G1 |
| IL18 |
| PPP4R2 |
| FUCA1 |
| PDE4DIP |
| S100A6 |
| MYO9B |
| GSN |
| FNIP2 |
| MAP1LC3B |
| MX1 |
| TAGAP |
| HLA-C |
| NDUFS3 |
| VEGFA |
| ARID5B |
| CD63 |
| GPR183 |
| ZNF267 |
| H3F3B |
| HERPUD1 |
| EEF1A1 |
| CXCL16 |
| GNA15 |
| LARP7 |
| ACSL1 |
| VCP |
| WASH4P |
| CLTA |
| JUN |
| INSIG1 |
| PLIN2 |
| C1QB |
| RALA |
| NAPA |
| FCGR2B |
| NFATC2IP |
| LPCAT1 |
| PPP1R15B |
| FOSL2 |
| HSPD1 |
| UBE2M |
| EIF4A3 |
| POLK |
| ST3GAL1 |
| TFRC |
| GADD45B |
| RIPK2 |
| DUSP4 |
| HCLS1 |
| RPL37 |
| RNASE1 |
| MS4A4A |
| LCP2 |
| NUMA1 |
| C19orf43 |
| MARCKSL1 |
| ARL4C |
| AKR1B1 |
| BIRC3 |
| DENND3 |
| VCAN |
| TOM1 |
| IFI35 |
| MT-CO3 |
| SLC3A2 |
| PSMA7 |
| ZFYVE16 |
| PTPN1 |
| ATP5B |
| H3F3A |
| ARFGAP3 |
| C19orf38 |
| RBX1 |
| CCS |
| UGP2 |
| EIF3F |
| RPL37A |
| DBI |
| SGK1 |
| HSPB1 |
| HBEGF |
| SDC2 |
| AOAH |
| RAB20 |
| NDUFS6 |
| NEDD8 |
| PFKFB3 |
| SLC16A10 |
| CYFIP1 |
| GLUL |
| RNH1 |
| EIF3H |
| WTAP |
| PSMA4 |
| CPVL |
| HNRNPK |
| DDIT4 |
| SLC43A3 |
| ACTR3 |
| RNF19B |
| ADAM8 |
| PGK1 |
| ATP5J2 |
| ERP29 |
| HSPA1A |
| DOK2 |
| ADM |
| SNX3 |
| GLIPR2 |
| SLC20A1 |
| POLR2L |
| ATP5H |
| MSN |
| SCARB2 |
| SPCS1 |
| NCOA4 |
| ATP5A1 |
| SRRM1 |
| ARL6IP4 |
| COX5B |
| RNMT |
| SYAP1 |
| LSM10 |
| ZNF106 |
| GRASP |
| FKBP1A |
| HSBP1 |
| SRSF9 |
| LINC00493 |
| CTSS |
| PTPN6 |
| ARRB2 |
| RPS19BP1 |
| MT1X |
| DYNLL1 |
| MIR24-2 |
| GLRX |
| RNF7 |
| ENY2 |
| VPS28 |
| RGCC |
| HSP90AA1 |
| MT-ATP8 |
| CLEC10A |
| DRAP1 |
| GLIPR1 |
| SPP1 |
| POLR1D |
| PRELID1 |
| CCRL2 |
| RETN |
| CANX |
| ACAA1 |
| TCIRG1 |
| UQCRFS1 |
| PSMB1 |
| CDKN1A |
| C14orf2 |
| TMBIM6 |
| CDV3 |
| RPS9 |
| TMEM59 |
| HK2 |
| PGLS |
| NAP1L1 |
| S100A4 |
| PSME2 |
| TALDO1 |
| DDT |
| ANKRD28 |
| BAX |
| RAC2 |
| HMGN2 |
| SHFM1 |
| ARPC4 |
| ZFAS1 |
| LAMTOR4 |
| MRPL20 |
| TUBA1A |
| SUMO1 |
| C7orf50 |
| THBD |
| LMO2 |
| TNFSF10 |
| RHOA |
| PSMB8 |
| TMA7 |
| TRIB1 |
| IFITM3 |
| ANAPC11 |
| HEBP2 |
| CTSZ |
| JTB |
| CD48 |
| PCBP1 |
| RNASET2 |
| PTP4A2 |
| EFHD2 |
| RAB10 |
| IQGAP2 |
| EIF4E2 |
| C1orf162 |
| SNX2 |
| PRAM1 |
| CD33 |
| DAB2 |
| PSMB9 |
| NUDT16 |
| ALOX5AP |
| AZIN1 |
| ZNHIT1 |
| SCPEP1 |
| WASF2 |
| PPP1R18 |
| ADRBK1 |
| PDCD6 |
| PPA1 |
| HMOX1 |
| CFP |
| LAMTOR5 |
| TRAF3IP3 |
| SCAND1 |
| WAS |
| AQP9 |
| LRP1 |
| SUMO2 |
| TMEM167A |
| ELL2 |
| TMBIM4.1 |
| H2AFY |
| FOXO3 |
| GSTK1 |
| FERMT3 |
| GTF2A2 |
| LGALS9 |
| SSR3 |
| ARHGAP18 |
| SERPINB1 |
| VIM |
| KIAA0930 |
| POLR2J |
| IFNGR2 |
| TMEM258 |
| CD52 |
| CAPZA1 |
| NDUFAF3 |
| PNP |
| MTPN |
| EIF3M |
| ACTG1 |
| SERP1 |
| FAM49B |
| CAST |
| ZBTB7A |
| KHDRBS1 |
| C11orf58 |
| ARL5A |
| NSA2 |
| AP2M1 |
| STAT3 |
| ANP32B |
| RBP7 |
| BANF1 |
| NDUFA11 |
| NDUFS2 |
| CD300A |
| TXNL4A |
| LILRA2 |
| RPL19 |
| NME3 |
| SNX10 |
| PAK1 |
| SKAP2 |
| RRAS |
| FAM204A |
| RPS18 |
| CTSA |
| VPS29 |
| RCSD1 |
| UBXN4 |
| PARVG |
| MTCH2 |
| BOD1L1 |
| CCT5 |
| GGNBP2 |
| SYF2 |
| FAM26F |
| SLC25A3 |
| NDUFB1 |
| RPL26 |
| SH3BGRL |
| LSM6 |
| PYURF |
| NACA |
| LSP1 |
| GNAI2 |
| POU2F2 |
| ICAM3 |
| FLI1 |
| POLE4 |
| CLNS1A |
| NAPRT1 |
| SHKBP1 |
| UBE2R2 |
| PGAM1 |
| ZC3H13 |
| EEF2 |
| ABRACL |
| C11orf31 |
| TERF2IP |
| SNRPC |
| PTRHD1 |
| THRAP3 |
| NOL7 |
| IKZF1 |
| MYO1F |
| ENO1 |
| LILRB2 |
| HLA-DRB1 |
| MNDA |
| LSMD1 |
| TMEM179B |
| BLOC1S1 |
| PAFAH1B1 |
| PDAP1 |
| MPHOSPH8 |
| XIST |
| STXBP2 |
| NPC2 |
| COTL1 |
| EVI2B |
| RPN2 |
| GNAQ |
| CAT |
| ANXA7 |
| OSTF1 |
| SNHG8 |
| S100A12 |
| DAPP1 |
| MSRB1 |
| TMEM205 |
| GCA |
| GIMAP7 |
| SEC11A |
| NUDC |
| PAPOLA |
| C20orf24 |
| TMPO |
| RGS19 |
| RIN3 |
| SMCO4 |
| ABCA1 |
| CCDC69 |
| CCND3 |
| HMGB2 |
| RB1CC1 |
| HSPA5 |
| MAPKAPK3 |
| GAA |
| HSPA4 |
| CHURC1 |
| TNFRSF1A |
| SNX17 |
| YWHAB |
| DOCK8 |
| RPL15 |
| SNX18 |
| CD300E |
| DECR1 |
| IER2 |
| CCNY |
| PTTG1IP |
| MFSD10 |
| ORMDL1 |
| SLC25A11 |
| S1PR4 |
| SDHC |
| PIH1D1 |
| CSK |
| MAPK14 |
| PSME1 |
| MTMR14 |
| AP1S2 |
| GRN |
| GAS7 |
| MBD2 |
| BLVRB |
| DBNL |
| RPS16 |
| ACADVL |
| CLINT1 |
| MEGF9 |
| ITGB1BP1 |
| CMTM7 |
| SSH2 |
| PLCB2 |
| NUP214 |
| FCGRT |
| WARS |
| ATP5G2 |
| PLP2 |
| RPS4X |
| FGR |
| SH3GLB1 |
| ECHDC1 |
| CDC40 |
| PKN1 |
| RPS8 |
| PILRA |
| UBE2J1 |
| NRGN |
| PDCD6IP |
| SNRPB2 |
| ATP5I |
| SURF1 |
| NAIP |
| OAS1 |
| UBE2N |
| SCIMP |
| CREB5 |
| PBRM1 |
| METTL7A |
| NUDT22 |
| CAPN1 |
| LRRK2 |
| RHOB |
| GABARAP |
| CUX1 |
| PCMT1 |
| PRMT2 |
| OSCAR |
| BTG2 |
| RPL36A |
| FRAT2 |
| FAM65B |
| HELZ |
| GTF3A |
| SET |
| MLEC |
| GBP2 |
| MIDN |
| DNAJB1 |
| PYCARD |
| ERO1L |
| HADHB |
| LAP3 |
| ECH1 |
| USP3 |
| RAB27A |
| CAPNS1 |
| GBAS |
| RNPEPL1 |
| CPNE1 |
| SURF4 |
| FKBP5 |
| LILRB1 |
| P4HA1 |
| ARPC5 |
| MCTS1 |
| FYB |
| ALOX5 |
| C1orf86 |
| CDA |
| PRDX5 |
| ASGR1 |
| ARPC3 |
| GSTP1 |
| RAB3D |
| MSL1 |
| KIDINS220 |
| HSD17B11 |
| C20orf27 |
| FPR1 |
| EMP3 |
| AGFG1 |
| STX10 |
| BRI3 |
| PUM1 |
| RPS10 |
| FBXL5 |
| PHC2 |
| CSTA |
| CYTIP |
| RAD23B |
| ANXA2 |
| SDHB |
| MIF |
| BLOC1S2 |
| ADAM10 |
| MAP3K1 |
| IRF2 |
| FAM200B |
| RPL17 |
| RPL4 |
| CEBPB |
| ZYX |
| CCR2 |
| SEC63 |
| MTIF3 |
| CCR1 |
| G6PD |
| ARHGDIB |
| GIMAP4 |
| SRPR |
| KIAA1551 |
| PTPN12 |
| TSPO |
| CCNDBP1 |
| PTPRC |
| MYD88 |
| CD46 |
