# Supplementary material for: Single-Cell Sequencing Analysis and Multiple Machine Learning Methods Identified G0S2 and HPSE as Novel Biomarkers for Abdominal Aortic Aneurysm
Source: Front Immunol. 2022 Jun 13;13:907309. doi: 10.3389/fimmu.2022.907309 (PMC9234288; doi:10.3389/fimmu.2022.907309)
Supplement: Supplementary Table 3 — All modular genes obtained from WGCNA. [file Table_3.doc]

**Supplementary table 3**

| Genes | Module Color |
| --- | --- |
| AACSL | blue |
| AADACL3 | blue |
| AATF | blue |
| AATK | blue |
| ABCB7 | blue |
| ACADSB | blue |
| ACAT1 | blue |
| ACBD5 | blue |
| ACCSL | blue |
| ACD | blue |
| ACE2 | blue |
| ACPP | blue |
| ACSM1 | blue |
| ADAM23 | blue |
| ADAM5P | blue |
| ADAMTS8 | blue |
| ADAMTS9 | blue |
| ADAMTSL5 | blue |
| ADCY4 | blue |
| ADCY9 | blue |
| ADH1B | blue |
| ADM | blue |
| ADO | blue |
| ADORA2A | blue |
| ADPRH | blue |
| ADRB1 | blue |
| ADRB2 | blue |
| AFAP1L2 | blue |
| AGBL2 | blue |
| AHSG | blue |
| AIM2 | blue |
| AKIRIN2 | blue |
| AKR1B1 | blue |
| AKR1C3 | blue |
| AKR1E2 | blue |
| ALDH1L2 | blue |
| ALDH9A1 | blue |
| ALG1 | blue |
| ALKBH2 | blue |
| ALKBH4 | blue |
| ALOX12 | blue |
| ALOXE3 | blue |
| ALS2CR4 | blue |
| AMY1A | blue |
| ANAPC2 | blue |
| ANKRD28 | blue |
| ANO4 | blue |
| ANO8 | blue |
| ANRIL | blue |
| ANXA13 | blue |
| ANXA5 | blue |
| ANXA8L2 | blue |
| AP1M1 | blue |
| APBB1IP | blue |
| APCDD1L | blue |
| APIP | blue |
| APOBEC1 | blue |
| APOBEC4 | blue |
| APOH | blue |
| APOOL | blue |
| AQP7 | blue |
| ARAP2 | blue |
| ARGLU1 | blue |
| ARHGAP11B | blue |
| ARHGAP20 | blue |
| ARHGAP29 | blue |
| ARHGEF2 | blue |
| ARHGEF9 | blue |
| ARIH1 | blue |
| ARL11 | blue |
| ARL9 | blue |
| ARMC10 | blue |
| ARMC6 | blue |
| ARPC3 | blue |
| ARPC5L | blue |
| ARPM1 | blue |
| ARSG | blue |
| ARSI | blue |
| ARVCF | blue |
| ASB3 | blue |
| ASCL2 | blue |
| ASF1B | blue |
| ASH1L | blue |
| ASH2L | blue |
| ASPM | blue |
| ASPN | blue |
| ATAD3B | blue |
| ATG4B | blue |
| ATOH8 | blue |
| ATP13A1 | blue |
| ATP13A3 | blue |
| ATP5B | blue |
| ATP6V0D2 | blue |
| ATP6V1G1 | blue |
| ATPGD1 | blue |
| ATXN7L1 | blue |
| ATXN8OS | blue |
| AUH | blue |
| AURKAPS1 | blue |
| AVIL | blue |
| B4GALT6 | blue |
| BBC3 | blue |
| BCAR3 | blue |
| BCKDK | blue |
| BCL2L11 | blue |
| BCL3 | blue |
| BCL7C | blue |
| BDP1 | blue |
| BET1 | blue |
| BET1L | blue |
| BHMT | blue |
| BICD1 | blue |
| BLID | blue |
| BLR1 | blue |
| BMP8A | blue |
| BOK | blue |
| BPHL | blue |
| BPI | blue |
| BRI3 | blue |
| BRIP1 | blue |
| BRMS1L | blue |
| BRSK1 | blue |
| BSG | blue |
| BST2 | blue |
| BTBD8 | blue |
| BUD31 | blue |
| BYSL | blue |
| BZRAP1 | blue |
| C10orf11 | blue |
| C10orf115 | blue |
| C10orf32 | blue |
| C10orf58 | blue |
| C10orf85 | blue |
| C10orf90 | blue |
| C11orf39 | blue |
| C11orf9 | blue |
| C12orf30 | blue |
| C12orf5 | blue |
| C12orf61 | blue |
| C12orf65 | blue |
| C13orf15 | blue |
| C13orf26 | blue |
| C14orf102 | blue |
| C14orf32 | blue |
| C14orf39 | blue |
| C14orf50 | blue |
| C14orf73 | blue |
| C15orf37 | blue |
| C15orf42 | blue |
| C15orf5 | blue |
| C16orf11 | blue |
| C16orf54 | blue |
| C16orf58 | blue |
| C16orf75 | blue |
| C16orf78 | blue |
| C17orf101 | blue |
| C17orf45 | blue |
| C17orf90 | blue |
| C18orf10 | blue |
| C19orf30 | blue |
| C19orf53 | blue |
| C19orf55 | blue |
| C19orf61 | blue |
| C19orf70 | blue |
| C1QL1 | blue |
| C1orf100 | blue |
| C1orf113 | blue |
| C1orf132 | blue |
| C1orf150 | blue |
| C1orf173 | blue |
| C1orf175 | blue |
| C1orf180 | blue |
| C1orf188 | blue |
| C1orf54 | blue |
| C1orf57 | blue |
| C1orf89 | blue |
| C1orf92 | blue |
| C1orf96 | blue |
| C20orf201 | blue |
| C20orf85 | blue |
| C20orf94 | blue |
| C20orf96 | blue |
| C21orf123 | blue |
| C2orf16 | blue |
| C3orf10 | blue |
| C3orf42 | blue |
| C3orf46 | blue |
| C4orf31 | blue |
| C4orf42 | blue |
| C5 | blue |
| C5orf13 | blue |
| C5orf30 | blue |
| C5orf54 | blue |
| C6orf130 | blue |
| C6orf162 | blue |
| C6orf204 | blue |
| C6orf218 | blue |
| C6orf225 | blue |
| C6orf57 | blue |
| C6orf66 | blue |
| C7orf34 | blue |
| C7orf42 | blue |
| C7orf50 | blue |
| C7orf53 | blue |
| C7orf63 | blue |
| C8orf40 | blue |
| C9orf150 | blue |
| C9orf21 | blue |
| C9orf36 | blue |
| C9orf4 | blue |
| C9orf40 | blue |
| C9orf45 | blue |
| C9orf78 | blue |
| C9orf82 | blue |
| C9orf9 | blue |
| CA3 | blue |
| CA6 | blue |
| CACNA1F | blue |
| CADM3 | blue |
| CALCOCO2 | blue |
| CALML3 | blue |
| CAMKK2 | blue |
| CAPN1 | blue |
| CATSPER4 | blue |
| CBLB | blue |
| CBLC | blue |
| CBWD6 | blue |
| CBX3 | blue |
| CCDC107 | blue |
| CCDC116 | blue |
| CCDC130 | blue |
| CCDC137 | blue |
| CCDC25 | blue |
| CCDC26 | blue |
| CCDC6 | blue |
| CCDC67 | blue |
| CCDC8 | blue |
| CCL23 | blue |
| CCL27 | blue |
| CCNI | blue |
| CCNL2 | blue |
| CD109 | blue |
| CD200 | blue |
| CD300E | blue |
| CD300LG | blue |
| CD3G | blue |
| CD69 | blue |
| CD7 | blue |
| CD72 | blue |
| CD74 | blue |
| CD99 | blue |
| CDC37 | blue |
| CDC42EP1 | blue |
| CDC45L | blue |
| CDC7 | blue |
| CDCA2 | blue |
| CDH12 | blue |
| CDH4 | blue |
| CDKN3 | blue |
| CDRT15P | blue |
| CDS2 | blue |
| CDX4 | blue |
| CEACAM1 | blue |
| CEACAM21 | blue |
| CEACAM3 | blue |
| CEBPA | blue |
| CELA2B | blue |
| CEMP1 | blue |
| CENPF | blue |
| CENPV | blue |
| CENTG2 | blue |
| CEP164 | blue |
| CEP27 | blue |
| CER1 | blue |
| CFC1B | blue |
| CFHR5 | blue |
| CGRRF1 | blue |
| CHD3 | blue |
| CHGA | blue |
| CHIC2 | blue |
| CHMP4B | blue |
| CHMP7 | blue |
| CHRNA3 | blue |
| CHST2 | blue |
| CHST8 | blue |
| CIDEB | blue |
| CISH | blue |
| CKM | blue |
| CLCN2 | blue |
| CLEC4G | blue |
| CLEC4GP1 | blue |
| CLIP4 | blue |
| CLLU1 | blue |
| CLP1 | blue |
| CLRN3 | blue |
| CLSTN3 | blue |
| CLUL1 | blue |
| CMKLR1 | blue |
| CMTM2 | blue |
| CMTM8 | blue |
| CNGA2 | blue |
| CNOT2 | blue |
| CNOT4 | blue |
| CNTF | blue |
| CNTLN | blue |
| CNTN6 | blue |
| COL24A1 | blue |
| COL6A1 | blue |
| COMMD5 | blue |
| COPE | blue |
| COPS3 | blue |
| COX7A2 | blue |
| CPA2 | blue |
| CPA4 | blue |
| CPA5 | blue |
| CPLX4 | blue |
| CPNE3 | blue |
| CRLF3 | blue |
| CRNN | blue |
| CROCC | blue |
| CRX | blue |
| CRY2 | blue |
| CSF3R | blue |
| CSGALNACT1 | blue |
| CSN2 | blue |
| CSNK1G2 | blue |
| CST9 | blue |
| CTTNBP2NL | blue |
| CTXN3 | blue |
| CUEDC2 | blue |
| CUL5 | blue |
| CWF19L1 | blue |
| CXCL1 | blue |
| CXorf26 | blue |
| CYB5RL | blue |
| CYP11B2 | blue |
| CYP1A2 | blue |
| CYP27A1 | blue |
| CYP39A1 | blue |
| CYP4F12 | blue |
| CYP4F8 | blue |
| CYYR1 | blue |
| DAAM2 | blue |
| DAOA | blue |
| DAPK2 | blue |
| DAPK3 | blue |
| DAXX | blue |
| DAZ2 | blue |
| DBR1 | blue |
| DCAKD | blue |
| DCST1 | blue |
| DCUN1D1 | blue |
| DDA1 | blue |
| DDAH2 | blue |
| DDX41 | blue |
| DDX49 | blue |
| DEFB103A | blue |
| DEFB110 | blue |
| DEFB118 | blue |
| DEFB119 | blue |
| DENND2C | blue |
| DENND3 | blue |
| DEPDC4 | blue |
| DERPC | blue |
| DHRS1 | blue |
| DHRS13 | blue |
| DHX16 | blue |
| DIABLO | blue |
| DKFZP434I0714 | blue |
| DKFZP434L187 | blue |
| DLAT | blue |
| DLG4 | blue |
| DLG5 | blue |
| DNA2 | blue |
| DNAJC14 | blue |
| DNAJC8 | blue |
| DNCL1 | blue |
| DNCL2A | blue |
| DOCK9 | blue |
| DPYS | blue |
| DR1 | blue |
| DRAP1 | blue |
| DSCR5 | blue |
| DTX3L | blue |
| DUSP1 | blue |
| DUSP15 | blue |
| DUSP18 | blue |
| DYNC1H1 | blue |
| DYNLL2 | blue |
| DYSF | blue |
| EAF2 | blue |
| EDA2R | blue |
| EDEM1 | blue |
| EEF1A2 | blue |
| EFCAB7 | blue |
| EFCBP1 | blue |
| EGFL7 | blue |
| EGLN3 | blue |
| EGR1 | blue |
| EID3 | blue |
| EIF2S1 | blue |
| EIF3A | blue |
| EIF4A1 | blue |
| EIF5A | blue |
| EIF6 | blue |
| ELK1 | blue |
| ELL2 | blue |
| ELOVL4 | blue |
| EMD | blue |
| EMID1 | blue |
| ENO2 | blue |
| ENTPD7 | blue |
| ENY2 | blue |
| EPHA3 | blue |
| EPHA7 | blue |
| EPM2AIP1 | blue |
| ERICH1 | blue |
| ERO1LB | blue |
| ERVWE1 | blue |
| ESR2 | blue |
| ETFDH | blue |
| ETV4 | blue |
| EXD3 | blue |
| EXOC3L2 | blue |
| EXOSC4 | blue |
| F11R | blue |
| F8A3 | blue |
| FADS2 | blue |
| FAM10A6 | blue |
| FAM116B | blue |
| FAM122B | blue |
| FAM126B | blue |
| FAM127A | blue |
| FAM128A | blue |
| FAM129B | blue |
| FAM149A | blue |
| FAM162B | blue |
| FAM164A | blue |
| FAM173B | blue |
| FAM19A5 | blue |
| FAM22F | blue |
| FAM38B | blue |
| FAM39DP | blue |
| FAM3A | blue |
| FAM46D | blue |
| FAM71D | blue |
| FAM75A7 | blue |
| FAM89B | blue |
| FAM91A1 | blue |
| FAM96A | blue |
| FASTKD1 | blue |
| FBXL18 | blue |
| FBXL3 | blue |
| FBXO41 | blue |
| FCGR1A | blue |
| FCGR3A | blue |
| FCHO1 | blue |
| FDPS | blue |
| FER1L3 | blue |
| FERD3L | blue |
| FERMT1 | blue |
| FERMT2 | blue |
| FEZF1 | blue |
| FGD2 | blue |
| FGF10 | blue |
| FGF12 | blue |
| FIG4 | blue |
| FILIP1 | blue |
| FKBP11 | blue |
| FKBP8 | blue |
| FLG | blue |
| FLJ10246 | blue |
| FLJ10781 | blue |
| FLJ14166 | blue |
| FLJ16734 | blue |
| FLJ21511 | blue |
| FLJ21865 | blue |
| FLJ23152 | blue |
| FLJ30679 | blue |
| FLJ32154 | blue |
| FLJ32569 | blue |
| FLJ33590 | blue |
| FLJ34306 | blue |
| FLJ35409 | blue |
| FLJ36492 | blue |
| FLJ41766 | blue |
| FLJ42393 | blue |
| FLJ42709 | blue |
| FLJ42953 | blue |
| FLJ43987 | blue |
| FLJ44379 | blue |
| FLJ90757 | blue |
| FLNB | blue |
| FLYWCH1 | blue |
| FMNL3 | blue |
| FMO2 | blue |
| FMO5 | blue |
| FOXH1 | blue |
| FOXJ3 | blue |
| FREQ | blue |
| FRG2 | blue |
| FRMPD2L1 | blue |
| FSTL3 | blue |
| FUT3 | blue |
| FUZ | blue |
| FZD3 | blue |
| GABRA1 | blue |
| GALNT12 | blue |
| GATA2 | blue |
| GATS | blue |
| GBX2 | blue |
| GCH1 | blue |
| GCN1L1 | blue |
| GDA | blue |
| GDF10 | blue |
| GDF9 | blue |
| GDI1 | blue |
| GEMIN6 | blue |
| GFI1B | blue |
| GFRA2 | blue |
| GGT7 | blue |
| GIMAP4 | blue |
| GINS1 | blue |
| GJC1 | blue |
| GLI4 | blue |
| GLT25D1 | blue |
| GNG11 | blue |
| GNGT2 | blue |
| GNPAT | blue |
| GOLGA7B | blue |
| GOLT1B | blue |
| GPIHBP1 | blue |
| GPR113 | blue |
| GPR133 | blue |
| GPR137B | blue |
| GPR139 | blue |
| GPR151 | blue |
| GPR156 | blue |
| GPR18 | blue |
| GPR61 | blue |
| GPR75 | blue |
| GPR88 | blue |
| GPR89B | blue |
| GPX1 | blue |
| GPX2 | blue |
| GRID1 | blue |
| GRIK5 | blue |
| GRIN2A | blue |
| GRM1 | blue |
| GRPEL1 | blue |
| GSG1L | blue |
| GSG2 | blue |
| GSS | blue |
| GTF2H3 | blue |
| GUCY2C | blue |
| GUCY2E | blue |
| GYS1 | blue |
| GZF1 | blue |
| GZMH | blue |
| GZMM | blue |
| H1FX | blue |
| H2AFZ | blue |
| HAAO | blue |
| HADH | blue |
| HAGH | blue |
| HAPLN4 | blue |
| HAS1 | blue |
| HBQ1 | blue |
| HCG9 | blue |
| HCLS1 | blue |
| HDAC11 | blue |
| HDAC3 | blue |
| HDGF | blue |
| HECTD2 | blue |
| HERC1 | blue |
| HERC6 | blue |
| HES4 | blue |
| HES5 | blue |
| HES7 | blue |
| HESX1 | blue |
| HEXDC | blue |
| HIST1H1C | blue |
| HIST1H2BG | blue |
| HIST1H2BM | blue |
| HIST1H3A | blue |
| HIST1H3H | blue |
| HIST1H4A | blue |
| HK2 | blue |
| HLA-A | blue |
| HLA-DMA | blue |
| HLA-DQB2 | blue |
| HLA-DRA | blue |
| HLA-DRB3 | blue |
| HLA-DRB6 | blue |
| HMG20A | blue |
| HMX3 | blue |
| HNRNPL | blue |
| HOM-TES-103 | blue |
| HOXA11 | blue |
| HOXA2 | blue |
| HOXA6 | blue |
| HOXD13 | blue |
| HPCA | blue |
| HRES1 | blue |
| HRH2 | blue |
| HSD17B1 | blue |
| HSD17B10 | blue |
| HSD17B7P2 | blue |
| HSD17B8 | blue |
| HSD3B1 | blue |
| HSH2D | blue |
| HSP90B1 | blue |
| HSP90B3P | blue |
| HSPB8 | blue |
| HSPBAP1 | blue |
| HTN1 | blue |
| HYOU1 | blue |
| IAH1 | blue |
| IDH1 | blue |
| IDH2 | blue |
| IFIH1 | blue |
| IFITM3 | blue |
| IFNA17 | blue |
| IFNA6 | blue |
| IFNB1 | blue |
| IFNG | blue |
| IFNGR2 | blue |
| IGSF10 | blue |
| IGSF2 | blue |
| IKBKE | blue |
| IKZF4 | blue |
| IL10RA | blue |
| IL11RA | blue |
| IL1F5 | blue |
| IL2 | blue |
| IL20RA | blue |
| IL20RB | blue |
| IL21 | blue |
| IL23A | blue |
| IL34 | blue |
| IMMT | blue |
| IMP5 | blue |
| IMPG2 | blue |
| INPP5D | blue |
| INS | blue |
| INSRR | blue |
| INTS12 | blue |
| INTS2 | blue |
| IQCE | blue |
| ITGA2 | blue |
| ITGAL | blue |
| ITGAM | blue |
| ITIH1 | blue |
| ITIH4 | blue |
| ITPR2 | blue |
| IYD | blue |
| JAM3 | blue |
| JARID1D | blue |
| JDP2 | blue |
| JMJD1A | blue |
| KARS | blue |
| KBTBD3 | blue |
| KCNA2 | blue |
| KCNE3 | blue |
| KCNG2 | blue |
| KCNJ3 | blue |
| KCNJ6 | blue |
| KCNK5 | blue |
| KCNK9 | blue |
| KCTD18 | blue |
| KCTD20 | blue |
| KCTD9 | blue |
| KDELC1 | blue |
| KHSRP | blue |
| KIAA0240 | blue |
| KIAA0514 | blue |
| KIAA0528 | blue |
| KIAA0562 | blue |
| KIAA0586 | blue |
| KIAA0776 | blue |
| KIAA0953 | blue |
| KIAA1009 | blue |
| KIAA1333 | blue |
| KIAA1467 | blue |
| KIAA1545 | blue |
| KIAA1586 | blue |
| KIAA2010 | blue |
| KIF6 | blue |
| KIFC1 | blue |
| KIR3DX1 | blue |
| KLC1 | blue |
| KLF1 | blue |
| KLHDC4 | blue |
| KLHDC5 | blue |
| KLHL1 | blue |
| KLHL35 | blue |
| KLK6 | blue |
| KPNA2 | blue |
| KREMEN2 | blue |
| KRT73 | blue |
| KRTAP1-3 | blue |
| KRTAP10-8 | blue |
| KRTAP12-4 | blue |
| KRTAP3-1 | blue |
| KRTAP4-1 | blue |
| KRTAP4-2 | blue |
| KRTAP5-10 | blue |
| LAS1L | blue |
| LHFP | blue |
| LHFPL4 | blue |
| LIMA1 | blue |
| LIX1L | blue |
| LMBR1L | blue |
| LMNA | blue |
| LNP1 | blue |
| LNX1 | blue |
| LOC122038 | blue |
| LOC124220 | blue |
| LOC127150 | blue |
| LOC143543 | blue |
| LOC144776 | blue |
| LOC145820 | blue |
| LOC150763 | blue |
| LOC151579 | blue |
| LOC152586 | blue |
| LOC169834 | blue |
| LOC199882 | blue |
| LOC220416 | blue |
| LOC253820 | blue |
| LOC255130 | blue |
| LOC255809 | blue |
| LOC283202 | blue |
| LOC283412 | blue |
| LOC283547 | blue |
| LOC283849 | blue |
| LOC283932 | blue |
| LOC284215 | blue |
| LOC284232 | blue |
| LOC284288 | blue |
| LOC284379 | blue |
| LOC284757 | blue |
| LOC284912 | blue |
| LOC285216 | blue |
| LOC285929 | blue |
| LOC338758 | blue |
| LOC338829 | blue |
| LOC339902 | blue |
| LOC341112 | blue |
| LOC341604 | blue |
| LOC343384 | blue |
| LOC344065 | blue |
| LOC344595 | blue |
| LOC347292 | blue |
| LOC374443 | blue |
| LOC387761 | blue |
| LOC387870 | blue |
| LOC388022 | blue |
| LOC388333 | blue |
| LOC388344 | blue |
| LOC388692 | blue |
| LOC388755 | blue |
| LOC388789 | blue |
| LOC388948 | blue |
| LOC389102 | blue |
| LOC389120 | blue |
| LOC389257 | blue |
| LOC389316 | blue |
| LOC389405 | blue |
| LOC389631 | blue |
| LOC389633 | blue |
| LOC389634 | blue |
| LOC389672 | blue |
| LOC389791 | blue |
| LOC389813 | blue |
| LOC390364 | blue |
| LOC390414 | blue |
| LOC391037 | blue |
| LOC391269 | blue |
| LOC391347 | blue |
| LOC391574 | blue |
| LOC391817 | blue |
| LOC392145 | blue |
| LOC392506 | blue |
| LOC399937 | blue |
| LOC400019 | blue |
| LOC400299 | blue |
| LOC400942 | blue |
| LOC400954 | blue |
| LOC400968 | blue |
| LOC401115 | blue |
| LOC401131 | blue |
| LOC401915 | blue |
| LOC401934 | blue |
| LOC402110 | blue |
| LOC402198 | blue |
| LOC402282 | blue |
| LOC402677 | blue |
| LOC402694 | blue |
| LOC404266 | blue |
| LOC439936 | blue |
| LOC439951 | blue |
| LOC440082 | blue |
| LOC440157 | blue |
| LOC440338 | blue |
| LOC440386 | blue |
| LOC440414 | blue |
| LOC440993 | blue |
| LOC441056 | blue |
| LOC441150 | blue |
| LOC441246 | blue |
| LOC441698 | blue |
| LOC441931 | blue |
| LOC442132 | blue |
| LOC442180 | blue |
| LOC442208 | blue |
| LOC442316 | blue |
| LOC442501 | blue |
| LOC442519 | blue |
| LOC442570 | blue |
| LOC54103 | blue |
| LOC541473 | blue |
| LOC553158 | blue |
| LOC641693 | blue |
| LOC641801 | blue |
| LOC641804 | blue |
| LOC641849 | blue |
| LOC641926 | blue |
| LOC641929 | blue |
| LOC641941 | blue |
| LOC641943 | blue |
| LOC641972 | blue |
| LOC641989 | blue |
| LOC641995 | blue |
| LOC642003 | blue |
| LOC642032 | blue |
| LOC642033 | blue |
| LOC642049 | blue |
| LOC642083 | blue |
| LOC642113 | blue |
| LOC642118 | blue |
| LOC642130 | blue |
| LOC642132 | blue |
| LOC642149 | blue |
| LOC642194 | blue |
| LOC642196 | blue |
| LOC642278 | blue |
| LOC642321 | blue |
| LOC642333 | blue |
| LOC642347 | blue |
| LOC642351 | blue |
| LOC642356 | blue |
| LOC642359 | blue |
| LOC642361 | blue |
| LOC642383 | blue |
| LOC642403 | blue |
| LOC642497 | blue |
| LOC642506 | blue |
| LOC642548 | blue |
| LOC642587 | blue |
| LOC642588 | blue |
| LOC642626 | blue |
| LOC642677 | blue |
| LOC642755 | blue |
| LOC642757 | blue |
| LOC642775 | blue |
| LOC642820 | blue |
| LOC642869 | blue |
| LOC642888 | blue |
| LOC642946 | blue |
| LOC642953 | blue |
| LOC642962 | blue |
| LOC643002 | blue |
| LOC643008 | blue |
| LOC643031 | blue |
| LOC643032 | blue |
| LOC643070 | blue |
| LOC643090 | blue |
| LOC643149 | blue |
| LOC643186 | blue |
| LOC643189 | blue |
| LOC643246 | blue |
| LOC643265 | blue |
| LOC643272 | blue |
| LOC643278 | blue |
| LOC643287 | blue |
| LOC643302 | blue |
| LOC643319 | blue |
| LOC643324 | blue |
| LOC643356 | blue |
| LOC643377 | blue |
| LOC643378 | blue |
| LOC643382 | blue |
| LOC643416 | blue |
| LOC643444 | blue |
| LOC643453 | blue |
| LOC643466 | blue |
| LOC643495 | blue |
| LOC643547 | blue |
| LOC643550 | blue |
| LOC643596 | blue |
| LOC643598 | blue |
| LOC643604 | blue |
| LOC643690 | blue |
| LOC643717 | blue |
| LOC643722 | blue |
| LOC643734 | blue |
| LOC643748 | blue |
| LOC643809 | blue |
| LOC643825 | blue |
| LOC643844 | blue |
| LOC643870 | blue |
| LOC643882 | blue |
| LOC643913 | blue |
| LOC643932 | blue |
| LOC643986 | blue |
| LOC643988 | blue |
| LOC644005 | blue |
| LOC644023 | blue |
| LOC644044 | blue |
| LOC644046 | blue |
| LOC644047 | blue |
| LOC644063 | blue |
| LOC644072 | blue |
| LOC644087 | blue |
| LOC644090 | blue |
| LOC644099 | blue |
| LOC644112 | blue |
| LOC644133 | blue |
| LOC644184 | blue |
| LOC644234 | blue |
| LOC644267 | blue |
| LOC644268 | blue |
| LOC644278 | blue |
| LOC644295 | blue |
| LOC644313 | blue |
| LOC644472 | blue |
| LOC644532 | blue |
| LOC644538 | blue |
| LOC644612 | blue |
| LOC644655 | blue |
| LOC644669 | blue |
| LOC644694 | blue |
| LOC644773 | blue |
| LOC644774 | blue |
| LOC644789 | blue |
| LOC644813 | blue |
| LOC644846 | blue |
| LOC644897 | blue |
| LOC644974 | blue |
| LOC645058 | blue |
| LOC645116 | blue |
| LOC645138 | blue |
| LOC645153 | blue |
| LOC645225 | blue |
| LOC645246 | blue |
| LOC645333 | blue |
| LOC645336 | blue |
| LOC645391 | blue |
| LOC645416 | blue |
| LOC645435 | blue |
| LOC645459 | blue |
| LOC645485 | blue |
| LOC645550 | blue |
| LOC645565 | blue |
| LOC645580 | blue |
| LOC645609 | blue |
| LOC645636 | blue |
| LOC645660 | blue |
| LOC645662 | blue |
| LOC645681 | blue |
| LOC645683 | blue |
| LOC645743 | blue |
| LOC645812 | blue |
| LOC645818 | blue |
| LOC645850 | blue |
| LOC645955 | blue |
| LOC646090 | blue |
| LOC646129 | blue |
| LOC646130 | blue |
| LOC646168 | blue |
| LOC646194 | blue |
| LOC646210 | blue |
| LOC646215 | blue |
| LOC646241 | blue |
| LOC646280 | blue |
| LOC646303 | blue |
| LOC646310 | blue |
| LOC646360 | blue |
| LOC646365 | blue |
| LOC646372 | blue |
| LOC646374 | blue |
| LOC646471 | blue |
| LOC646509 | blue |
| LOC646552 | blue |
| LOC646561 | blue |
| LOC646574 | blue |
| LOC646625 | blue |
| LOC646638 | blue |
| LOC646686 | blue |
| LOC646717 | blue |
| LOC646748 | blue |
| LOC646795 | blue |
| LOC646796 | blue |
| LOC646813 | blue |
| LOC646858 | blue |
| LOC646861 | blue |
| LOC646882 | blue |
| LOC646917 | blue |
| LOC646997 | blue |
| LOC647049 | blue |
| LOC647130 | blue |
| LOC647176 | blue |
| LOC647191 | blue |
| LOC647206 | blue |
| LOC647295 | blue |
| LOC647331 | blue |
| LOC647349 | blue |
| LOC647417 | blue |
| LOC647468 | blue |
| LOC647471 | blue |
| LOC647480 | blue |
| LOC647509 | blue |
| LOC647527 | blue |
| LOC647536 | blue |
| LOC647540 | blue |
| LOC647542 | blue |
| LOC647551 | blue |
| LOC647585 | blue |
| LOC647646 | blue |
| LOC647651 | blue |
| LOC647677 | blue |
| LOC647704 | blue |
| LOC647707 | blue |
| LOC647713 | blue |
| LOC647802 | blue |
| LOC648000 | blue |
| LOC648003 | blue |
| LOC648024 | blue |
| LOC648041 | blue |
| LOC648058 | blue |
| LOC648080 | blue |
| LOC648099 | blue |
| LOC648133 | blue |
| LOC648164 | blue |
| LOC648169 | blue |
| LOC648245 | blue |
| LOC648256 | blue |
| LOC648257 | blue |
| LOC648365 | blue |
| LOC648447 | blue |
| LOC648456 | blue |
| LOC648469 | blue |
| LOC648473 | blue |
| LOC648489 | blue |
| LOC648496 | blue |
| LOC648533 | blue |
| LOC648552 | blue |
| LOC648556 | blue |
| LOC648596 | blue |
| LOC648605 | blue |
| LOC648608 | blue |
| LOC648744 | blue |
| LOC648801 | blue |
| LOC648827 | blue |
| LOC648855 | blue |
| LOC648926 | blue |
| LOC648974 | blue |
| LOC648979 | blue |
| LOC648997 | blue |
| LOC649009 | blue |
| LOC649025 | blue |
| LOC649060 | blue |
| LOC649075 | blue |
| LOC649086 | blue |
| LOC649128 | blue |
| LOC649137 | blue |
| LOC649169 | blue |
| LOC649198 | blue |
| LOC649383 | blue |
| LOC649504 | blue |
| LOC649542 | blue |
| LOC649548 | blue |
| LOC649593 | blue |
| LOC649700 | blue |
| LOC649771 | blue |
| LOC649826 | blue |
| LOC649853 | blue |
| LOC649857 | blue |
| LOC649963 | blue |
| LOC649993 | blue |
| LOC650036 | blue |
| LOC650037 | blue |
| LOC650116 | blue |
| LOC650167 | blue |
| LOC650188 | blue |
| LOC650243 | blue |
| LOC650251 | blue |
| LOC650293 | blue |
| LOC650314 | blue |
| LOC650329 | blue |
| LOC650361 | blue |
| LOC650373 | blue |
| LOC650390 | blue |
| LOC650407 | blue |
| LOC650433 | blue |
| LOC650599 | blue |
| LOC650680 | blue |
| LOC650681 | blue |
| LOC650689 | blue |
| LOC650724 | blue |
| LOC650749 | blue |
| LOC650781 | blue |
| LOC650823 | blue |
| LOC650845 | blue |
| LOC650867 | blue |
| LOC650883 | blue |
| LOC650922 | blue |
| LOC651096 | blue |
| LOC651143 | blue |
| LOC651289 | blue |
| LOC651315 | blue |
| LOC651316 | blue |
| LOC651495 | blue |
| LOC651513 | blue |
| LOC651537 | blue |
| LOC651577 | blue |
| LOC651752 | blue |
| LOC651872 | blue |
| LOC651886 | blue |
| LOC651896 | blue |
| LOC651916 | blue |
| LOC651936 | blue |
| LOC651966 | blue |
| LOC651987 | blue |
| LOC652002 | blue |
| LOC652100 | blue |
| LOC652113 | blue |
| LOC652126 | blue |
| LOC652173 | blue |
| LOC652185 | blue |
| LOC652233 | blue |
| LOC652272 | blue |
| LOC652290 | blue |
| LOC652305 | blue |
| LOC652367 | blue |
| LOC652454 | blue |
| LOC652466 | blue |
| LOC652553 | blue |
| LOC652646 | blue |
| LOC652679 | blue |
| LOC652712 | blue |
| LOC652745 | blue |
| LOC652779 | blue |
| LOC652803 | blue |
| LOC652828 | blue |
| LOC652831 | blue |
| LOC652839 | blue |
| LOC652840 | blue |
| LOC652848 | blue |
| LOC652883 | blue |
| LOC652906 | blue |
| LOC653034 | blue |
| LOC653052 | blue |
| LOC653066 | blue |
| LOC653118 | blue |
| LOC653163 | blue |
| LOC653189 | blue |
| LOC653204 | blue |
| LOC653257 | blue |
| LOC653269 | blue |
| LOC653280 | blue |
| LOC653293 | blue |
| LOC653338 | blue |
| LOC653346 | blue |
| LOC653425 | blue |
| LOC653429 | blue |
| LOC653431 | blue |
| LOC653437 | blue |
| LOC653481 | blue |
| LOC653483 | blue |
| LOC653513 | blue |
| LOC653514 | blue |
| LOC653527 | blue |
| LOC653559 | blue |
| LOC653563 | blue |
| LOC653576 | blue |
| LOC653609 | blue |
| LOC653648 | blue |
| LOC653676 | blue |
| LOC653701 | blue |
| LOC653706 | blue |
| LOC653711 | blue |
| LOC653748 | blue |
| LOC653778 | blue |
| LOC653781 | blue |
| LOC653809 | blue |
| LOC653852 | blue |
| LOC653853 | blue |
| LOC653874 | blue |
| LOC653888 | blue |
| LOC654101 | blue |
| LOC654109 | blue |
| LOC654113 | blue |
| LOC654115 | blue |
| LOC654117 | blue |
| LOC654155 | blue |
| LOC654187 | blue |
| LOC654235 | blue |
| LOC654340 | blue |
| LOC727815 | blue |
| LOC727944 | blue |
| LOC728193 | blue |
| LOC728285 | blue |
| LOC728403 | blue |
| LOC728504 | blue |
| LOC728518 | blue |
| LOC728636 | blue |
| LOC728747 | blue |
| LOC729196 | blue |
| LOC729393 | blue |
| LOC729766 | blue |
| LOC730015 | blue |
| LOC730249 | blue |
| LOC730262 | blue |
| LOC730546 | blue |
| LOC730740 | blue |
| LOC730760 | blue |
| LOC730818 | blue |
| LOC731007 | blue |
| LOC731049 | blue |
| LOC731158 | blue |
| LOC731835 | blue |
| LOC732111 | blue |
| LOC732172 | blue |
| LOC732425 | blue |
| LOC85389 | blue |
| LOC85391 | blue |
| LOC92154 | blue |
| LOH3CR2A | blue |
| LOXL3 | blue |
| LPCAT3 | blue |
| LPCAT4 | blue |
| LPP | blue |
| LPPR2 | blue |
| LRCH2 | blue |
| LRFN4 | blue |
| LRRC31 | blue |
| LRRC42 | blue |
| LRRC49 | blue |
| LRRC61 | blue |
| LRRTM1 | blue |
| LRTM1 | blue |
| LTBP1 | blue |
| LTBR | blue |
| LTV1 | blue |
| LUZP4 | blue |
| LY75 | blue |
| LYN | blue |
| LYPD3 | blue |
| LYPD4 | blue |
| LYPD6B | blue |
| LZTFL1 | blue |
| MACF1 | blue |
| MAGEA12 | blue |
| MAGEA8 | blue |
| MAGEL2 | blue |
| MAGOH | blue |
| MAP3K12 | blue |
| MAP3K8 | blue |
| MAP4K1 | blue |
| MAP4K2 | blue |
| MAP6D1 | blue |
| MAPK12 | blue |
| MARK3 | blue |
| MASP2 | blue |
| MCART6 | blue |
| MEA1 | blue |
| MED24 | blue |
| MESDC1 | blue |
| MEX3C | blue |
| MFSD10 | blue |
| MFSD3 | blue |
| MFSD6 | blue |
| MFSD6L | blue |
| MGAT1 | blue |
| MGAT4A | blue |
| MGAT5 | blue |
| MGC16169 | blue |
| MGC24125 | blue |
| MGC27121 | blue |
| MGC3020 | blue |
| MGC33556 | blue |
| MGC40499 | blue |
| MGC40574 | blue |
| MGC52282 | blue |
| MGC61598 | blue |
| MGEA5 | blue |
| MICAL2 | blue |
| MICALCL | blue |
| MKRN3 | blue |
| MLL4 | blue |
| MMGT1 | blue |
| MMP11 | blue |
| MMP3 | blue |
| MMPL1 | blue |
| MMRN1 | blue |
| MND1 | blue |
| MON1A | blue |
| MORF4 | blue |
| MORN2 | blue |
| MPV17 | blue |
| MRPL12 | blue |
| MRPL41 | blue |
| MRPL50 | blue |
| MRTO4 | blue |
| MS4A15 | blue |
| MSL3 | blue |
| MSLNL | blue |
| MSMP | blue |
| MST1R | blue |
| MT1L | blue |
| MTERFD2 | blue |
| MTHFD2 | blue |
| MTMR7 | blue |
| MYL9 | blue |
| MYO15B | blue |
| MYO6 | blue |
| MYOG | blue |
| MYPOP | blue |
| MYSM1 | blue |
| NACC1 | blue |
| NAGK | blue |
| NAGPA | blue |
| NASP | blue |
| NAT12 | blue |
| NBAS | blue |
| NBEA | blue |
| NCAPD2 | blue |
| NCOA6 | blue |
| NCOA7 | blue |
| NCR3 | blue |
| NCSTN | blue |
| NDUFA11 | blue |
| NDUFAF1 | blue |
| NDUFB11 | blue |
| NDUFB7 | blue |
| NECAB1 | blue |
| NEGR1 | blue |
| NEUROD2 | blue |
| NEXN | blue |
| NFATC4 | blue |
| NFS1 | blue |
| NGFR | blue |
| NHLRC2 | blue |
| NIF3L1 | blue |
| NIP7 | blue |
| NIPA1 | blue |
| NIPBL | blue |
| NKIRAS1 | blue |
| NKX1-2 | blue |
| NKX2-3 | blue |
| NLRP11 | blue |
| NMU | blue |
| NNT | blue |
| NOS1AP | blue |
| NOTO | blue |
| NOXA1 | blue |
| NPL | blue |
| NPM2 | blue |
| NPR1 | blue |
| NR2E1 | blue |
| NR5A2 | blue |
| NRG4 | blue |
| NRIP2 | blue |
| NRXN1 | blue |
| NT5C1A | blue |
| NT5C3 | blue |
| NTSR2 | blue |
| NUCB1 | blue |
| NUDT18 | blue |
| NUDT5 | blue |
| NUP37 | blue |
| NUS1 | blue |
| OAF | blue |
| OCIAD1 | blue |
| OCM | blue |
| ODZ1 | blue |
| OLFML2B | blue |
| OR10AG1 | blue |
| OR10H2 | blue |
| OR10K1 | blue |
| OR11G2 | blue |
| OR13H1 | blue |
| OR1J4 | blue |
| OR1L1 | blue |
| OR2B2 | blue |
| OR2L3 | blue |
| OR2L8 | blue |
| OR4D1 | blue |
| OR51B6 | blue |
| OR51D1 | blue |
| OR52E8 | blue |
| OR52L1 | blue |
| OR56B1 | blue |
| OR5B2 | blue |
| OR5H14 | blue |
| OR5M3 | blue |
| OR6C6 | blue |
| OR7A17 | blue |
| OR8B4 | blue |
| OR8D4 | blue |
| OSCAR | blue |
| OSMR | blue |
| OSTalpha | blue |
| OVCA2 | blue |
| OVCH1 | blue |
| OXSM | blue |
| P4HA2 | blue |
| P704P | blue |
| PACAP | blue |
| PACS1 | blue |
| PAFAH1B1 | blue |
| PAFAH1B3 | blue |
| PALM | blue |
| PAPOLA | blue |
| PAPSS2 | blue |
| PARD6B | blue |
| PARD6G | blue |
| PAX4 | blue |
| PCBP3 | blue |
| PCDHB15 | blue |
| PCDHB18 | blue |
| PCDHGB2 | blue |
| PCIF1 | blue |
| PCK1 | blue |
| PCNT | blue |
| PCP4 | blue |
| PCYT1A | blue |
| PDAP1 | blue |
| PDCD1LG2 | blue |
| PDCD2L | blue |
| PDE1B | blue |
| PDHX | blue |
| PDIA4 | blue |
| PDLIM4 | blue |
| PDSS2 | blue |
| PDZD3 | blue |
| PDZK1 | blue |
| PEG3 | blue |
| PEMT | blue |
| PEX16 | blue |
| PGAP3 | blue |
| PGBD4 | blue |
| PHCA | blue |
| PHF1 | blue |
| PHF20L1 | blue |
| PHKA1 | blue |
| PHKG2 | blue |
| PHLDA1 | blue |
| PI4KAP2 | blue |
| PIGO | blue |
| PIH1D2 | blue |
| PIK3C2B | blue |
| PIK3CB | blue |
| PINX1 | blue |
| PIP5K3 | blue |
| PITPNM2 | blue |
| PKD1 | blue |
| PKD1L1 | blue |
| PKD1L3 | blue |
| PKD2L2 | blue |
| PKNOX2 | blue |
| PLA2G1B | blue |
| PLA2G2F | blue |
| PLAC2 | blue |
| PLAGL1 | blue |
| PLCB2 | blue |
| PLCG2 | blue |
| PLEKHA3 | blue |
| PLEKHH1 | blue |
| PLEKHM1 | blue |
| PLEKHO2 | blue |
| PLXNA1 | blue |
| PLXND1 | blue |
| PMAIP1 | blue |
| PMM2 | blue |
| PNMA5 | blue |
| POLD1 | blue |
| POLR1D | blue |
| POLR2J | blue |
| POLR2K | blue |
| PON1 | blue |
| POP5 | blue |
| PPCDC | blue |
| PPIF | blue |
| PPM1D | blue |
| PPP1R13L | blue |
| PPP1R3F | blue |
| PPP2R2D | blue |
| PPP4R4 | blue |
| PRAMEF6 | blue |
| PRDX5 | blue |
| PRKAB2 | blue |
| PRKRIP1 | blue |
| PRLHR | blue |
| PRMT3 | blue |
| PRNP | blue |
| PROCA1 | blue |
| PRODH | blue |
| PROZ | blue |
| PRPF31 | blue |
| PRPF4 | blue |
| PRRC1 | blue |
| PRRG2 | blue |
| PRSS16 | blue |
| PRSS22 | blue |
| PRSS48 | blue |
| PRX | blue |
| PSAT1 | blue |
| PSG4 | blue |
| PSG6 | blue |
| PSMB5 | blue |
| PSMD3 | blue |
| PSMD5 | blue |
| PTCD1 | blue |
| PTCH1 | blue |
| PTGS1 | blue |
| PTH2R | blue |
| PTK9 | blue |
| PTPLA | blue |
| PTPN11 | blue |
| PTPN4 | blue |
| PUF60 | blue |
| PURB | blue |
| PUS10 | blue |
| PYDC1 | blue |
| RAB15 | blue |
| RAB31 | blue |
| RAB3GAP1 | blue |
| RAB40A | blue |
| RAB7L1 | blue |
| RABGGTA | blue |
| RAD17 | blue |
| RAET1E | blue |
| RAPGEF1 | blue |
| RARG | blue |
| RARRES2 | blue |
| RASD2 | blue |
| RASGRF2 | blue |
| RBM10 | blue |
| RBM11 | blue |
| RBM34 | blue |
| RBM44 | blue |
| RBM8A | blue |
| RDBP | blue |
| RDH8 | blue |
| RESP18 | blue |
| RFTN1 | blue |
| RFX6 | blue |
| RGL3 | blue |
| RGS6 | blue |
| RHOD | blue |
| RING1 | blue |
| RIPK3 | blue |
| RLBP1 | blue |
| RLTPR | blue |
| RNF123 | blue |
| RNF13 | blue |
| RNF169 | blue |
| RNF17 | blue |
| RNF20 | blue |
| RNF217 | blue |
| RNF6 | blue |
| RNFT1 | blue |
| RNPS1 | blue |
| ROBO1 | blue |
| RORB | blue |
| RPF2 | blue |
| RPL26 | blue |
| RPL34 | blue |
| RPL3L | blue |
| RPN2 | blue |
| RPP38 | blue |
| RPRD1A | blue |
| RPS18 | blue |
| RPS19 | blue |
| RPS6KA3 | blue |
| RPS6KB2 | blue |
| RPUSD3 | blue |
| RRAGD | blue |
| RRP15 | blue |
| RSPH9 | blue |
| RTTN | blue |
| RUNDC2C | blue |
| RUVBL1 | blue |
| RYR3 | blue |
| S100A11 | blue |
| SAA2 | blue |
| SALL3 | blue |
| SAT1 | blue |
| SATB1 | blue |
| SAV1 | blue |
| SBF1 | blue |
| SC65 | blue |
| SCAF1 | blue |
| SCG2 | blue |
| SCGN | blue |
| SCLT1 | blue |
| SCLY | blue |
| SCN10A | blue |
| SCN11A | blue |
| SCRG1 | blue |
| SCRT2 | blue |
| SDC4P | blue |
| SDCCAG8 | blue |
| SDSL | blue |
| SEC23A | blue |
| SELI | blue |
| SELV | blue |
| SEMA4A | blue |
| SENP8 | blue |
| SERHL | blue |
| SERPIND1 | blue |
| SETD8 | blue |
| SFPQ | blue |
| SFRS16 | blue |
| SFTPA1 | blue |
| SH2D6 | blue |
| SH3BP5L | blue |
| SH3D19 | blue |
| SH3MD4 | blue |
| SHISA5 | blue |
| SHOC2 | blue |
| SIAE | blue |
| SIN3A | blue |
| SIX5 | blue |
| SLAMF8 | blue |
| SLC10A4 | blue |
| SLC12A8 | blue |
| SLC16A4 | blue |
| SLC17A2 | blue |
| SLC17A8 | blue |
| SLC17A9 | blue |
| SLC1A2 | blue |
| SLC22A18 | blue |
| SLC22A6 | blue |
| SLC24A3 | blue |
| SLC25A41 | blue |
| SLC25A46 | blue |
| SLC29A4 | blue |
| SLC30A9 | blue |
| SLC35A3 | blue |
| SLC35F5 | blue |
| SLC38A2 | blue |
| SLC38A3 | blue |
| SLC38A6 | blue |
| SLC39A11 | blue |
| SLC39A12 | blue |
| SLC39A7 | blue |
| SLC5A1 | blue |
| SLC5A9 | blue |
| SLC6A6 | blue |
| SLC9A2 | blue |
| SLCO1B3 | blue |
| SLITRK1 | blue |
| SLITRK2 | blue |
| SLN | blue |
| SMARCD1 | blue |
| SMARCE1 | blue |
| SNAP23 | blue |
| SNAPC4 | blue |
| SNHG5 | blue |
| SNORA62 | blue |
| SNORA70 | blue |
| SNORD116-2 | blue |
| SNORD13 | blue |
| SNORD30 | blue |
| SNORD4B | blue |
| SNX10 | blue |
| SNX12 | blue |
| SNX26 | blue |
| SNX31 | blue |
| SORCS2 | blue |
| SOX15 | blue |
| SOX21 | blue |
| SOX9 | blue |
| SP3 | blue |
| SPAG4L | blue |
| SPANXC | blue |
| SPATA1 | blue |
| SPATA4 | blue |
| SPATA5 | blue |
| SPCS1 | blue |
| SPG11 | blue |
| SPIB | blue |
| SPINK5L2 | blue |
| SPRR1A | blue |
| SPRR2F | blue |
| SPSB1 | blue |
| SPSB3 | blue |
| SRP68 | blue |
| SRPR | blue |
| SSFA2 | blue |
| SSSCA1 | blue |
| STAT5B | blue |
| STAT6 | blue |
| STC2 | blue |
| STK11 | blue |
| STK11IP | blue |
| STK38L | blue |
| STMN1 | blue |
| STX12 | blue |
| STXBP4 | blue |
| STYXL1 | blue |
| SURF1 | blue |
| SYNGR4 | blue |
| SYT9 | blue |
| TAAR8 | blue |
| TAF4B | blue |
| TAL2 | blue |
| TAS2R3 | blue |
| TAS2R49 | blue |
| TATDN2 | blue |
| TBC1D10C | blue |
| TBC1D2B | blue |
| TBC1D3C | blue |
| TBCC | blue |
| TBKBP1 | blue |
| TBL1X | blue |
| TBN | blue |
| TBX21 | blue |
| TCBA1 | blue |
| TCEAL4 | blue |
| TCF4 | blue |
| TCN2 | blue |
| TEC | blue |
| TERF2IP | blue |
| TFB2M | blue |
| TFEB | blue |
| TFF1 | blue |
| TFF2 | blue |
| TGFB1 | blue |
| TGFB3 | blue |
| TGFBRAP1 | blue |
| THG1L | blue |
| TIGD7 | blue |
| TIMM8B | blue |
| TIMP4 | blue |
| TLR2 | blue |
| TLR7 | blue |
| TM9SF2 | blue |
| TMC1 | blue |
| TMED5 | blue |
| TMEM100 | blue |
| TMEM104 | blue |
| TMEM105 | blue |
| TMEM130 | blue |
| TMEM14C | blue |
| TMEM151 | blue |
| TMEM161A | blue |
| TMEM167A | blue |
| TMEM189-UBE2V1 | blue |
| TMEM200B | blue |
| TMEM38A | blue |
| TMEM4 | blue |
| TMEM57 | blue |
| TMEM82 | blue |
| TMEM86B | blue |
| TMEM87A | blue |
| TMEM9B | blue |
| TNFRSF8 | blue |
| TNK1 | blue |
| TNKS | blue |
| TNNC1 | blue |
| TNS3 | blue |
| TOP1 | blue |
| TOPORS | blue |
| TOR1B | blue |
| TP53BP1 | blue |
| TPM1 | blue |
| TRA1P2 | blue |
| TRAPPC5 | blue |
| TRAPPC6A | blue |
| TRERF1 | blue |
| TRIM22 | blue |
| TRIM25 | blue |
| TRIM27 | blue |
| TRIM45 | blue |
| TRIM74 | blue |
| TRIP11 | blue |
| TRIT1 | blue |
| TRMT11 | blue |
| TRMT6 | blue |
| TRPM4 | blue |
| TRY6 | blue |
| TSPAN14 | blue |
| TSPAN31 | blue |
| TSPY3 | blue |
| TSR2 | blue |
| TST | blue |
| TSTA3 | blue |
| TTBK2 | blue |
| TTC23L | blue |
| TTC35 | blue |
| TTF2 | blue |
| TTTY14 | blue |
| TTYH3 | blue |
| TUBB6 | blue |
| TULP4 | blue |
| TUSC2 | blue |
| TUT1 | blue |
| TWF2 | blue |
| TXN | blue |
| TYK2 | blue |
| TYW3 | blue |
| UBE2N | blue |
| UBE2W | blue |
| UBE2Z | blue |
| UBL4A | blue |
| UGCG | blue |
| UGT1A1 | blue |
| UGT1A5 | blue |
| ULBP1 | blue |
| UPK1A | blue |
| USP12 | blue |
| USP3 | blue |
| USP33 | blue |
| UTP18 | blue |
| UTX | blue |
| VANGL2 | blue |
| VAT1 | blue |
| VCX-C | blue |
| VDAC3 | blue |
| VENTX | blue |
| VENTXP7 | blue |
| VEZF1 | blue |
| VEZT | blue |
| VN1R1 | blue |
| VNN1 | blue |
| VPREB3 | blue |
| VPS26A | blue |
| VPS36 | blue |
| VPS39 | blue |
| VPS52 | blue |
| VPS8 | blue |
| VSIG2 | blue |
| VWF | blue |
| WASF3 | blue |
| WASL | blue |
| WDR12 | blue |
| WDR5B | blue |
| WDR61 | blue |
| WDR81 | blue |
| WDR85 | blue |
| WFIKKN1 | blue |
| WNT4 | blue |
| XAB2 | blue |
| XAGE1B | blue |
| XKR5 | blue |
| XPO1 | blue |
| XRCC3 | blue |
| YARS | blue |
| YBX1 | blue |
| YIPF3 | blue |
| ZAN | blue |
| ZAR1 | blue |
| ZBTB2 | blue |
| ZC3H12A | blue |
| ZC3H12B | blue |
| ZCCHC16 | blue |
| ZDHHC19 | blue |
| ZDHHC3 | blue |
| ZDHHC6 | blue |
| ZFP95 | blue |
| ZFPM1 | blue |
| ZFPM2 | blue |
| ZIC1 | blue |
| ZIC5 | blue |
| ZIK1 | blue |
| ZMYM6 | blue |
| ZNF10 | blue |
| ZNF114 | blue |
| ZNF131 | blue |
| ZNF155 | blue |
| ZNF175 | blue |
| ZNF219 | blue |
| ZNF224 | blue |
| ZNF239 | blue |
| ZNF25 | blue |
| ZNF268 | blue |
| ZNF275 | blue |
| ZNF282 | blue |
| ZNF300 | blue |
| ZNF33B | blue |
| ZNF395 | blue |
| ZNF409 | blue |
| ZNF419 | blue |
| ZNF436 | blue |
| ZNF486 | blue |
| ZNF490 | blue |
| ZNF502 | blue |
| ZNF518A | blue |
| ZNF527 | blue |
| ZNF530 | blue |
| ZNF532 | blue |
| ZNF606 | blue |
| ZNF616 | blue |
| ZNF653 | blue |
| ZNF663 | blue |
| ZNF664 | blue |
| ZNF687 | blue |
| ZNF70 | blue |
| ZNF717 | blue |
| ZNF718 | blue |
| ZNF780A | blue |
| ZNF79 | blue |
| ZPBP2 | blue |
| ZZEF1 | blue |
| AACS | greenyellow |
| AANAT | greenyellow |
| AARS | greenyellow |
| ABCA1 | greenyellow |
| ABCC11 | greenyellow |
| ABCC12 | greenyellow |
| ABI2 | greenyellow |
| ACADVL | greenyellow |
| ACAN | greenyellow |
| ACAT2 | greenyellow |
| ACO2 | greenyellow |
| ACOT9 | greenyellow |
| ACTL6B | greenyellow |
| ACTN4 | greenyellow |
| ACTR6 | greenyellow |
| ACYP1 | greenyellow |
| ADAM20 | greenyellow |
| ADCY8 | greenyellow |
| AFF2 | greenyellow |
| AFF3 | greenyellow |
| AFMID | greenyellow |
| AFP | greenyellow |
| AGRP | greenyellow |
| AGXT2L1 | greenyellow |
| AHSA1 | greenyellow |
| AKAP5 | greenyellow |
| AKAP8 | greenyellow |
| ALDH1A3 | greenyellow |
| ALDOB | greenyellow |
| ALG14 | greenyellow |
| ALG2 | greenyellow |
| ALS2CR16 | greenyellow |
| AMIGO3 | greenyellow |
| AMOT | greenyellow |
| AMT | greenyellow |
| AMZ2 | greenyellow |
| ANGPT4 | greenyellow |
| ANKHD1-EIF4EBP3 | greenyellow |
| ANKRD10 | greenyellow |
| ANKRD24 | greenyellow |
| ANKRD55 | greenyellow |
| ANKS4B | greenyellow |
| ANP32B | greenyellow |
| ANXA3 | greenyellow |
| ANXA4 | greenyellow |
| ANXA6 | greenyellow |
| AP3D1 | greenyellow |
| APBA3 | greenyellow |
| APCS | greenyellow |
| APEX1 | greenyellow |
| APLN | greenyellow |
| APRIN | greenyellow |
| APTX | greenyellow |
| ARCN1 | greenyellow |
| ARHGAP17 | greenyellow |
| ARHGDIB | greenyellow |
| ARHGEF10L | greenyellow |
| ARL6IP5 | greenyellow |
| ARPC4 | greenyellow |
| ARPP-21 | greenyellow |
| ARSB | greenyellow |
| ATCAY | greenyellow |
| ATE1 | greenyellow |
| ATP10A | greenyellow |
| ATP13A2 | greenyellow |
| ATP1A4 | greenyellow |
| ATP6V1E2 | greenyellow |
| ATP8B3 | greenyellow |
| AVPR1B | greenyellow |
| B3GALT6 | greenyellow |
| B3GAT2 | greenyellow |
| B4GALT3 | greenyellow |
| BACE1 | greenyellow |
| BAG1 | greenyellow |
| BAGE3 | greenyellow |
| BBS1 | greenyellow |
| BCAS2 | greenyellow |
| BCOR | greenyellow |
| BCYRN1 | greenyellow |
| BDKRB2 | greenyellow |
| BMS1 | greenyellow |
| BPY2B | greenyellow |
| BRMS1 | greenyellow |
| BRWD2 | greenyellow |
| BTBD11 | greenyellow |
| BTNL9 | greenyellow |
| BUD13 | greenyellow |
| C10orf25 | greenyellow |
| C10orf72 | greenyellow |
| C10orf99 | greenyellow |
| C12orf10 | greenyellow |
| C12orf64 | greenyellow |
| C13orf34 | greenyellow |
| C14orf169 | greenyellow |
| C14orf85 | greenyellow |
| C15orf54 | greenyellow |
| C16orf3 | greenyellow |
| C16orf70 | greenyellow |
| C16orf90 | greenyellow |
| C17orf108 | greenyellow |
| C17orf48 | greenyellow |
| C17orf50 | greenyellow |
| C17orf71 | greenyellow |
| C19orf51 | greenyellow |
| C1orf118 | greenyellow |
| C1orf123 | greenyellow |
| C1orf137 | greenyellow |
| C1orf177 | greenyellow |
| C1orf35 | greenyellow |
| C2 | greenyellow |
| C20orf11 | greenyellow |
| C20orf177 | greenyellow |
| C20orf30 | greenyellow |
| C20orf54 | greenyellow |
| C21orf62 | greenyellow |
| C21orf84 | greenyellow |
| C2CD4A | greenyellow |
| C2orf19 | greenyellow |
| C2orf54 | greenyellow |
| C4orf27 | greenyellow |
| C4orf36 | greenyellow |
| C4orf41 | greenyellow |
| C6orf111 | greenyellow |
| C6orf114 | greenyellow |
| C6orf129 | greenyellow |
| C6orf165 | greenyellow |
| C6orf35 | greenyellow |
| C7orf10 | greenyellow |
| C7orf68 | greenyellow |
| C7orf70 | greenyellow |
| C8orf17 | greenyellow |
| C8orf58 | greenyellow |
| C9orf102 | greenyellow |
| C9orf125 | greenyellow |
| C9orf31 | greenyellow |
| C9orf50 | greenyellow |
| CABP4 | greenyellow |
| CACNA2D4 | greenyellow |
| CACNB4 | greenyellow |
| CACNG2 | greenyellow |
| CACNG8 | greenyellow |
| CAMK1 | greenyellow |
| CAMLG | greenyellow |
| CAND1 | greenyellow |
| CANX | greenyellow |
| CASC1 | greenyellow |
| CASC3 | greenyellow |
| CASKIN1 | greenyellow |
| CAV2 | greenyellow |
| CAV3 | greenyellow |
| CBS | greenyellow |
| CC2D2A | greenyellow |
| CCDC127 | greenyellow |
| CCDC146 | greenyellow |
| CCDC148 | greenyellow |
| CCDC16 | greenyellow |
| CCDC57 | greenyellow |
| CCDC65 | greenyellow |
| CCDC86 | greenyellow |
| CCK | greenyellow |
| CCL18 | greenyellow |
| CCND3 | greenyellow |
| CCNDBP1 | greenyellow |
| CCNG1 | greenyellow |
| CCT2 | greenyellow |
| CD1B | greenyellow |
| CD52 | greenyellow |
| CDADC1 | greenyellow |
| CDC23 | greenyellow |
| CDC25B | greenyellow |
| CDC2L1 | greenyellow |
| CDC2L2 | greenyellow |
| CDC42SE2 | greenyellow |
| CDC73 | greenyellow |
| CDCA1 | greenyellow |
| CDH8 | greenyellow |
| CDRT15 | greenyellow |
| CEP68 | greenyellow |
| CGB7 | greenyellow |
| CHCHD4 | greenyellow |
| CHES1 | greenyellow |
| CHRNB1 | greenyellow |
| CHRNB3 | greenyellow |
| CHST15 | greenyellow |
| CHSY1 | greenyellow |
| CILP2 | greenyellow |
| CLCA1 | greenyellow |
| CLDN8 | greenyellow |
| CLIP1 | greenyellow |
| CLK4 | greenyellow |
| CLOCK | greenyellow |
| CLSPN | greenyellow |
| CLU | greenyellow |
| CMAH | greenyellow |
| CNGA1 | greenyellow |
| CNGA3 | greenyellow |
| CNGB1 | greenyellow |
| CNOT8 | greenyellow |
| COBL | greenyellow |
| COG4 | greenyellow |
| COMMD1 | greenyellow |
| CORO2B | greenyellow |
| CPEB4 | greenyellow |
| CPT1B | greenyellow |
| CPXM1 | greenyellow |
| CRB2 | greenyellow |
| CRTAM | greenyellow |
| CRYL1 | greenyellow |
| CSAG2 | greenyellow |
| CSK | greenyellow |
| CSN1S2A | greenyellow |
| CSNK1D | greenyellow |
| CSPG4LYP1 | greenyellow |
| CSTA | greenyellow |
| CTAG1B | greenyellow |
| CTAGE1 | greenyellow |
| CTSA | greenyellow |
| CTSO | greenyellow |
| CUL7 | greenyellow |
| CXCL10 | greenyellow |
| CXCL9 | greenyellow |
| CXorf39 | greenyellow |
| CYB5R1 | greenyellow |
| CYB5R3 | greenyellow |
| CYC1 | greenyellow |
| CYP19A1 | greenyellow |
| CYP26C1 | greenyellow |
| CYP2A6 | greenyellow |
| CYTH3 | greenyellow |
| DAZ1 | greenyellow |
| DBH | greenyellow |
| DCTD | greenyellow |
| DCTN1 | greenyellow |
| DCTN3 | greenyellow |
| DDX10 | greenyellow |
| DDX60 | greenyellow |
| DEFA1B | greenyellow |
| DEFA4 | greenyellow |
| DEFB106A | greenyellow |
| DEFB127 | greenyellow |
| DENR | greenyellow |
| DES | greenyellow |
| DGKH | greenyellow |
| DGKK | greenyellow |
| DHRS12 | greenyellow |
| DKFZP686E2158 | greenyellow |
| DKFZP781G0119 | greenyellow |
| DLGAP4 | greenyellow |
| DMRT2 | greenyellow |
| DMRTB1 | greenyellow |
| DMXL1 | greenyellow |
| DNAI1 | greenyellow |
| DNAJB13 | greenyellow |
| DNAJB4 | greenyellow |
| DNAJC5G | greenyellow |
| DNHL1 | greenyellow |
| DOK2 | greenyellow |
| DPP4 | greenyellow |
| DPY19L2 | greenyellow |
| DRD3 | greenyellow |
| DUS3L | greenyellow |
| DUSP14 | greenyellow |
| DUSP22 | greenyellow |
| DUSP27 | greenyellow |
| DUX4C | greenyellow |
| DUXA | greenyellow |
| DYDC2 | greenyellow |
| EBF3 | greenyellow |
| ECD | greenyellow |
| ECEL1 | greenyellow |
| ECHDC3 | greenyellow |
| EFHA2 | greenyellow |
| EFTUD1 | greenyellow |
| EHD4 | greenyellow |
| EHF | greenyellow |
| EIF2AK1 | greenyellow |
| EIF2B5 | greenyellow |
| EIF2S2 | greenyellow |
| EIF3I | greenyellow |
| ELL3 | greenyellow |
| ELOVL3 | greenyellow |
| ELP4 | greenyellow |
| EMCN | greenyellow |
| EMP3 | greenyellow |
| EN2 | greenyellow |
| ENSA | greenyellow |
| ENTPD5 | greenyellow |
| EOMES | greenyellow |
| EPHA5 | greenyellow |
| EPS15L1 | greenyellow |
| EPS8L3 | greenyellow |
| ERAS | greenyellow |
| ERCC1 | greenyellow |
| ERF | greenyellow |
| ERVK6 | greenyellow |
| ESPL1 | greenyellow |
| ESRRB | greenyellow |
| EXOSC10 | greenyellow |
| F3 | greenyellow |
| FAAH | greenyellow |
| FAHD2A | greenyellow |
| FAM101A | greenyellow |
| FAM105B | greenyellow |
| FAM110C | greenyellow |
| FAM113A | greenyellow |
| FAM113B | greenyellow |
| FAM171B | greenyellow |
| FAM175B | greenyellow |
| FAM188B | greenyellow |
| FAM3D | greenyellow |
| FAM44A | greenyellow |
| FAM5C | greenyellow |
| FAM64A | greenyellow |
| FAM72D | greenyellow |
| FAM75A3 | greenyellow |
| FAM84A | greenyellow |
| FAT3 | greenyellow |
| FBF1 | greenyellow |
| FBXL2 | greenyellow |
| FBXO18 | greenyellow |
| FBXO31 | greenyellow |
| FBXO6 | greenyellow |
| FCAMR | greenyellow |
| FEM1A | greenyellow |
| FER1L5 | greenyellow |
| FERMT3 | greenyellow |
| FFAR3 | greenyellow |
| FGD4 | greenyellow |
| FGF21 | greenyellow |
| FGF9 | greenyellow |
| FGFR2 | greenyellow |
| FGG | greenyellow |
| FLJ13224 | greenyellow |
| FLJ20489 | greenyellow |
| FLJ25404 | greenyellow |
| FLJ25996 | greenyellow |
| FLJ30719 | greenyellow |
| FLJ34969 | greenyellow |
| FLJ38717 | greenyellow |
| FLJ40288 | greenyellow |
| FLJ42102 | greenyellow |
| FLJ43663 | greenyellow |
| FLJ43980 | greenyellow |
| FLJ44005 | greenyellow |
| FLJ44606 | greenyellow |
| FLJ44861 | greenyellow |
| FLJ45974 | greenyellow |
| FLJ46347 | greenyellow |
| FLRT1 | greenyellow |
| FLYWCH2 | greenyellow |
| FNBP1L | greenyellow |
| FNBP4 | greenyellow |
| FNTB | greenyellow |
| FPR2 | greenyellow |
| FUK | greenyellow |
| FUT8 | greenyellow |
| G0S2 | greenyellow |
| GABRD | greenyellow |
| GADD45A | greenyellow |
| GAGE8 | greenyellow |
| GALNT10 | greenyellow |
| GALR1 | greenyellow |
| GAPDH | greenyellow |
| GAS2L1 | greenyellow |
| GATAD2A | greenyellow |
| GDF5 | greenyellow |
| GFI1 | greenyellow |
| GFM1 | greenyellow |
| GFPT1 | greenyellow |
| GGH | greenyellow |
| GGTLC1 | greenyellow |
| GHR | greenyellow |
| GHRHR | greenyellow |
| GJC3 | greenyellow |
| GLIPR1L1 | greenyellow |
| GLT6D1 | greenyellow |
| GLTSCR2 | greenyellow |
| GM2A | greenyellow |
| GNAI3 | greenyellow |
| GNAL | greenyellow |
| GNB4 | greenyellow |
| GNL3L | greenyellow |
| GPHN | greenyellow |
| GPN2 | greenyellow |
| GPR150 | greenyellow |
| GPR179 | greenyellow |
| GPR3 | greenyellow |
| GPR45 | greenyellow |
| GPR63 | greenyellow |
| GPRC5C | greenyellow |
| GSTA2 | greenyellow |
| GSTM1L | greenyellow |
| GTF2F2 | greenyellow |
| GTF3C5 | greenyellow |
| GUCY1A2 | greenyellow |
| GZMB | greenyellow |
| HARBI1 | greenyellow |
| HAVCR2 | greenyellow |
| HCFC1 | greenyellow |
| HCG3 | greenyellow |
| HCG4 | greenyellow |
| HEATR2 | greenyellow |
| HEATR4 | greenyellow |
| HELT | greenyellow |
| HELZ | greenyellow |
| HIP1R | greenyellow |
| HIST1H1A | greenyellow |
| HIST1H2AA | greenyellow |
| HIST1H2AE | greenyellow |
| HIST1H2AG | greenyellow |
| HIST1H2BI | greenyellow |
| HLA-F | greenyellow |
| HNF4A | greenyellow |
| HNRPUL1 | greenyellow |
| HNRPUL2 | greenyellow |
| HOXC8 | greenyellow |
| HOXD3 | greenyellow |
| HSD11B2 | greenyellow |
| HTR7 | greenyellow |
| HTR7P | greenyellow |
| HYAL2 | greenyellow |
| HYPB | greenyellow |
| ICOSLG | greenyellow |
| IDS | greenyellow |
| IFLTD1 | greenyellow |
| IFNA1 | greenyellow |
| IFNA4 | greenyellow |
| IFNAR1 | greenyellow |
| IFNAR2 | greenyellow |
| IFP38 | greenyellow |
| IFT52 | greenyellow |
| IGFBP6 | greenyellow |
| IK | greenyellow |
| IKZF5 | greenyellow |
| IL1R1 | greenyellow |
| IMMP2L | greenyellow |
| IMP3 | greenyellow |
| IMPAD1 | greenyellow |
| INS-IGF2 | greenyellow |
| IQGAP3 | greenyellow |
| ITGB7 | greenyellow |
| IWS1 | greenyellow |
| JAG1 | greenyellow |
| JAKMIP2 | greenyellow |
| KCNE1L | greenyellow |
| KCNK16 | greenyellow |
| KCNQ3 | greenyellow |
| KCNS1 | greenyellow |
| KCNT2 | greenyellow |
| KIAA0020 | greenyellow |
| KIAA0133 | greenyellow |
| KIAA0368 | greenyellow |
| KIAA0391 | greenyellow |
| KIAA0853 | greenyellow |
| KIAA0907 | greenyellow |
| KIAA1274 | greenyellow |
| KIAA1324 | greenyellow |
| KIAA1383 | greenyellow |
| KIAA1553 | greenyellow |
| KIAA1671 | greenyellow |
| KIAA1737 | greenyellow |
| KIAA1826 | greenyellow |
| KIAA1841 | greenyellow |
| KIF18B | greenyellow |
| KIF21B | greenyellow |
| KIF2C | greenyellow |
| KIF5C | greenyellow |
| KIRREL2 | greenyellow |
| KLF11 | greenyellow |
| KLF4 | greenyellow |
| KLHL36 | greenyellow |
| KLK3 | greenyellow |
| KNTC1 | greenyellow |
| KRT10 | greenyellow |
| KRTAP1-5 | greenyellow |
| KRTAP6-3 | greenyellow |
| KRTCAP2 | greenyellow |
| KU-MEL-3 | greenyellow |
| L3MBTL4 | greenyellow |
| LAPTM4A | greenyellow |
| LARGE | greenyellow |
| LARP6 | greenyellow |
| LASS2 | greenyellow |
| LATS1 | greenyellow |
| LCE2B | greenyellow |
| LCNL1 | greenyellow |
| LDB2 | greenyellow |
| LDLR | greenyellow |
| LEO1 | greenyellow |
| LFNG | greenyellow |
| LGALS9C | greenyellow |
| LGMN | greenyellow |
| LIG4 | greenyellow |
| LILRB2 | greenyellow |
| LIN28 | greenyellow |
| LINGO1 | greenyellow |
| LMBRD1 | greenyellow |
| LMO2 | greenyellow |
| LNPEP | greenyellow |
| LOC124512 | greenyellow |
| LOC130773 | greenyellow |
| LOC144383 | greenyellow |
| LOC150786 | greenyellow |
| LOC152667 | greenyellow |
| LOC153328 | greenyellow |
| LOC155060 | greenyellow |
| LOC158730 | greenyellow |
| LOC161527 | greenyellow |
| LOC196752 | greenyellow |
| LOC200493 | greenyellow |
| LOC201140 | greenyellow |
| LOC284998 | greenyellow |
| LOC285479 | greenyellow |
| LOC338756 | greenyellow |
| LOC340228 | greenyellow |
| LOC340549 | greenyellow |
| LOC344165 | greenyellow |
| LOC387723 | greenyellow |
| LOC388476 | greenyellow |
| LOC388578 | greenyellow |
| LOC388720 | greenyellow |
| LOC388813 | greenyellow |
| LOC389394 | greenyellow |
| LOC389842 | greenyellow |
| LOC390282 | greenyellow |
| LOC391475 | greenyellow |
| LOC399744 | greenyellow |
| LOC399939 | greenyellow |
| LOC400236 | greenyellow |
| LOC400298 | greenyellow |
| LOC400509 | greenyellow |
| LOC401068 | greenyellow |
| LOC401164 | greenyellow |
| LOC401238 | greenyellow |
| LOC401525 | greenyellow |
| LOC401923 | greenyellow |
| LOC402279 | greenyellow |
| LOC402571 | greenyellow |
| LOC402617 | greenyellow |
| LOC402635 | greenyellow |
| LOC440402 | greenyellow |
| LOC440421 | greenyellow |
| LOC440438 | greenyellow |
| LOC440570 | greenyellow |
| LOC440926 | greenyellow |
| LOC441019 | greenyellow |
| LOC441061 | greenyellow |
| LOC441155 | greenyellow |
| LOC441546 | greenyellow |
| LOC441792 | greenyellow |
| LOC441956 | greenyellow |
| LOC442517 | greenyellow |
| LOC497256 | greenyellow |
| LOC504188 | greenyellow |
| LOC51145 | greenyellow |
| LOC51152 | greenyellow |
| LOC641699 | greenyellow |
| LOC641751 | greenyellow |
| LOC641810 | greenyellow |
| LOC641964 | greenyellow |
| LOC641977 | greenyellow |
| LOC642035 | greenyellow |
| LOC642155 | greenyellow |
| LOC642181 | greenyellow |
| LOC642280 | greenyellow |
| LOC642365 | greenyellow |
| LOC642424 | greenyellow |
| LOC642505 | greenyellow |
| LOC642576 | greenyellow |
| LOC642639 | greenyellow |
| LOC642646 | greenyellow |
| LOC642650 | greenyellow |
| LOC642718 | greenyellow |
| LOC642731 | greenyellow |
| LOC642787 | greenyellow |
| LOC642816 | greenyellow |
| LOC642902 | greenyellow |
| LOC642966 | greenyellow |
| LOC642969 | greenyellow |
| LOC643085 | greenyellow |
| LOC643100 | greenyellow |
| LOC643147 | greenyellow |
| LOC643206 | greenyellow |
| LOC643231 | greenyellow |
| LOC643313 | greenyellow |
| LOC643339 | greenyellow |
| LOC643346 | greenyellow |
| LOC643381 | greenyellow |
| LOC643429 | greenyellow |
| LOC643509 | greenyellow |
| LOC643558 | greenyellow |
| LOC643563 | greenyellow |
| LOC643757 | greenyellow |
| LOC643771 | greenyellow |
| LOC643897 | greenyellow |
| LOC643901 | greenyellow |
| LOC643910 | greenyellow |
| LOC643968 | greenyellow |
| LOC644066 | greenyellow |
| LOC644075 | greenyellow |
| LOC644096 | greenyellow |
| LOC644116 | greenyellow |
| LOC644136 | greenyellow |
| LOC644262 | greenyellow |
| LOC644310 | greenyellow |
| LOC644320 | greenyellow |
| LOC644322 | greenyellow |
| LOC644327 | greenyellow |
| LOC644330 | greenyellow |
| LOC644336 | greenyellow |
| LOC644444 | greenyellow |
| LOC644473 | greenyellow |
| LOC644484 | greenyellow |
| LOC644489 | greenyellow |
| LOC644499 | greenyellow |
| LOC644599 | greenyellow |
| LOC644623 | greenyellow |
| LOC644670 | greenyellow |
| LOC644677 | greenyellow |
| LOC644683 | greenyellow |
| LOC644701 | greenyellow |
| LOC644705 | greenyellow |
| LOC644725 | greenyellow |
| LOC644748 | greenyellow |
| LOC644762 | greenyellow |
| LOC644802 | greenyellow |
| LOC644809 | greenyellow |
| LOC644994 | greenyellow |
| LOC645099 | greenyellow |
| LOC645362 | greenyellow |
| LOC645441 | greenyellow |
| LOC645553 | greenyellow |
| LOC645563 | greenyellow |
| LOC645783 | greenyellow |
| LOC645899 | greenyellow |
| LOC645929 | greenyellow |
| LOC646033 | greenyellow |
| LOC646135 | greenyellow |
| LOC646358 | greenyellow |
| LOC646363 | greenyellow |
| LOC646373 | greenyellow |
| LOC646407 | greenyellow |
| LOC646439 | greenyellow |
| LOC646449 | greenyellow |
| LOC646503 | greenyellow |
| LOC646665 | greenyellow |
| LOC646675 | greenyellow |
| LOC646783 | greenyellow |
| LOC646803 | greenyellow |
| LOC646863 | greenyellow |
| LOC646897 | greenyellow |
| LOC646951 | greenyellow |
| LOC646990 | greenyellow |
| LOC647034 | greenyellow |
| LOC647042 | greenyellow |
| LOC647079 | greenyellow |
| LOC647135 | greenyellow |
| LOC647174 | greenyellow |
| LOC647237 | greenyellow |
| LOC647279 | greenyellow |
| LOC647460 | greenyellow |
| LOC647500 | greenyellow |
| LOC647571 | greenyellow |
| LOC647579 | greenyellow |
| LOC647645 | greenyellow |
| LOC647726 | greenyellow |
| LOC647783 | greenyellow |
| LOC647832 | greenyellow |
| LOC647869 | greenyellow |
| LOC647935 | greenyellow |
| LOC647989 | greenyellow |
| LOC648050 | greenyellow |
| LOC648145 | greenyellow |
| LOC648174 | greenyellow |
| LOC648200 | greenyellow |
| LOC648405 | greenyellow |
| LOC648500 | greenyellow |
| LOC648976 | greenyellow |
| LOC649292 | greenyellow |
| LOC649337 | greenyellow |
| LOC649340 | greenyellow |
| LOC649341 | greenyellow |
| LOC649385 | greenyellow |
| LOC649436 | greenyellow |
| LOC649493 | greenyellow |
| LOC649524 | greenyellow |
| LOC649571 | greenyellow |
| LOC649620 | greenyellow |
| LOC649747 | greenyellow |
| LOC649754 | greenyellow |
| LOC649902 | greenyellow |
| LOC649978 | greenyellow |
| LOC650006 | greenyellow |
| LOC650140 | greenyellow |
| LOC650214 | greenyellow |
| LOC650268 | greenyellow |
| LOC650280 | greenyellow |
| LOC650323 | greenyellow |
| LOC650494 | greenyellow |
| LOC650496 | greenyellow |
| LOC650538 | greenyellow |
| LOC650628 | greenyellow |
| LOC650695 | greenyellow |
| LOC650716 | greenyellow |
| LOC650771 | greenyellow |
| LOC650836 | greenyellow |
| LOC650841 | greenyellow |
| LOC650843 | greenyellow |
| LOC650849 | greenyellow |
| LOC651012 | greenyellow |
| LOC651073 | greenyellow |
| LOC651116 | greenyellow |
| LOC651140 | greenyellow |
| LOC651175 | greenyellow |
| LOC651212 | greenyellow |
| LOC651400 | greenyellow |
| LOC651467 | greenyellow |
| LOC651556 | greenyellow |
| LOC651610 | greenyellow |
| LOC651695 | greenyellow |
| LOC651760 | greenyellow |
| LOC651892 | greenyellow |
| LOC652003 | greenyellow |
| LOC652009 | greenyellow |
| LOC652070 | greenyellow |
| LOC652119 | greenyellow |
| LOC652134 | greenyellow |
| LOC652197 | greenyellow |
| LOC652202 | greenyellow |
| LOC652216 | greenyellow |
| LOC652368 | greenyellow |
| LOC652377 | greenyellow |
| LOC652396 | greenyellow |
| LOC652404 | greenyellow |
| LOC652418 | greenyellow |
| LOC652424 | greenyellow |
| LOC652495 | greenyellow |
| LOC652523 | greenyellow |
| LOC652533 | greenyellow |
| LOC652565 | greenyellow |
| LOC652570 | greenyellow |
| LOC652595 | greenyellow |
| LOC652597 | greenyellow |
| LOC652635 | greenyellow |
| LOC652692 | greenyellow |
| LOC652826 | greenyellow |
| LOC652871 | greenyellow |
| LOC652903 | greenyellow |
| LOC653051 | greenyellow |
| LOC653081 | greenyellow |
| LOC653082 | greenyellow |
| LOC653119 | greenyellow |
| LOC653125 | greenyellow |
| LOC653165 | greenyellow |
| LOC653175 | greenyellow |
| LOC653199 | greenyellow |
| LOC653225 | greenyellow |
| LOC653241 | greenyellow |
| LOC653310 | greenyellow |
| LOC653394 | greenyellow |
| LOC653451 | greenyellow |
| LOC653480 | greenyellow |
| LOC653506 | greenyellow |
| LOC653589 | greenyellow |
| LOC653623 | greenyellow |
| LOC653808 | greenyellow |
| LOC653841 | greenyellow |
| LOC653883 | greenyellow |
| LOC654106 | greenyellow |
| LOC654121 | greenyellow |
| LOC654203 | greenyellow |
| LOC654258 | greenyellow |
| LOC727935 | greenyellow |
| LOC728003 | greenyellow |
| LOC728181 | greenyellow |
| LOC728219 | greenyellow |
| LOC728343 | greenyellow |
| LOC728441 | greenyellow |
| LOC728481 | greenyellow |
| LOC728621 | greenyellow |
| LOC728637 | greenyellow |
| LOC728654 | greenyellow |
| LOC728709 | greenyellow |
| LOC729175 | greenyellow |
| LOC729269 | greenyellow |
| LOC729602 | greenyellow |
| LOC729675 | greenyellow |
| LOC729744 | greenyellow |
| LOC729751 | greenyellow |
| LOC729759 | greenyellow |
| LOC730050 | greenyellow |
| LOC731000 | greenyellow |
| LOC731085 | greenyellow |
| LOC731777 | greenyellow |
| LOC731884 | greenyellow |
| LONP2 | greenyellow |
| LRP11 | greenyellow |
| LRPAP1 | greenyellow |
| LRRC2 | greenyellow |
| LRRC37A | greenyellow |
| LRRC39 | greenyellow |
| LRRC43 | greenyellow |
| LRSAM1 | greenyellow |
| LSM2 | greenyellow |
| LSM5 | greenyellow |
| LSMD1 | greenyellow |
| LYPLA2P1 | greenyellow |
| LYSMD3 | greenyellow |
| LZTR1 | greenyellow |
| M160 | greenyellow |
| MADD | greenyellow |
| MAP4K4 | greenyellow |
| MAP7D2 | greenyellow |
| MAST4 | greenyellow |
| MBL2 | greenyellow |
| MBP | greenyellow |
| MCART2 | greenyellow |
| MCHR1 | greenyellow |
| MCOLN2 | greenyellow |
| MED21 | greenyellow |
| MED4 | greenyellow |
| MELK | greenyellow |
| METTL6 | greenyellow |
| MEX3B | greenyellow |
| MFAP5 | greenyellow |
| MFSD11 | greenyellow |
| MFSD2 | greenyellow |
| MGC102966 | greenyellow |
| MGC27165 | greenyellow |
| MGC35361 | greenyellow |
| MGC40170 | greenyellow |
| MGC71993 | greenyellow |
| MGC88374 | greenyellow |
| MIER3 | greenyellow |
| MIF4GD | greenyellow |
| MIS12 | greenyellow |
| MITD1 | greenyellow |
| MKI67 | greenyellow |
| MMP9 | greenyellow |
| MPP3 | greenyellow |
| MPP5 | greenyellow |
| MPRIP | greenyellow |
| MRC1L1 | greenyellow |
| MREG | greenyellow |
| MRM1 | greenyellow |
| MRPS23 | greenyellow |
| MS4A8B | greenyellow |
| MST1 | greenyellow |
| MT1X | greenyellow |
| MTF1 | greenyellow |
| MTMR10 | greenyellow |
| MVK | greenyellow |
| MYO10 | greenyellow |
| MYST3 | greenyellow |
| N-PAC | greenyellow |
| N6AMT2 | greenyellow |
| NANOS3 | greenyellow |
| NARG2 | greenyellow |
| NBPF20 | greenyellow |
| NBPF4 | greenyellow |
| NCF1 | greenyellow |
| NDFIP2 | greenyellow |
| NEDD4L | greenyellow |
| NEFH | greenyellow |
| NEIL1 | greenyellow |
| NEU1 | greenyellow |
| NFE2L1 | greenyellow |
| NFIC | greenyellow |
| NFIL3 | greenyellow |
| NGB | greenyellow |
| NID2 | greenyellow |
| NIT1 | greenyellow |
| NKG7 | greenyellow |
| NKPD1 | greenyellow |
| NKX6-3 | greenyellow |
| NLGN3 | greenyellow |
| NLGN4X | greenyellow |
| NLRP1 | greenyellow |
| NLRP13 | greenyellow |
| NMUR1 | greenyellow |
| NOBOX | greenyellow |
| NOS2A | greenyellow |
| NOSIP | greenyellow |
| NPHP1 | greenyellow |
| NPR2 | greenyellow |
| NPY6R | greenyellow |
| NRARP | greenyellow |
| NRAS | greenyellow |
| NRXN2 | greenyellow |
| NSBP1 | greenyellow |
| NSMAF | greenyellow |
| NSUN7 | greenyellow |
| NTF5 | greenyellow |
| NTRK1 | greenyellow |
| NUCKS1 | greenyellow |
| NUDT11 | greenyellow |
| NUDT4P1 | greenyellow |
| NUP153 | greenyellow |
| NUP160 | greenyellow |
| NUPR1 | greenyellow |
| NUTF2 | greenyellow |
| NWD1 | greenyellow |
| NXF5 | greenyellow |
| NXT2 | greenyellow |
| NY-REN-7 | greenyellow |
| OAT | greenyellow |
| OLFM3 | greenyellow |
| OLR1 | greenyellow |
| OMD | greenyellow |
| OPN1LW | greenyellow |
| OR13C3 | greenyellow |
| OR13C9 | greenyellow |
| OR1D4 | greenyellow |
| OR1E2 | greenyellow |
| OR2K2 | greenyellow |
| OR2M3 | greenyellow |
| OR3A2 | greenyellow |
| OR4K17 | greenyellow |
| OR51F1 | greenyellow |
| OR52B4 | greenyellow |
| OR5B17 | greenyellow |
| OR5K3 | greenyellow |
| OR5T1 | greenyellow |
| OR6S1 | greenyellow |
| OR7D4 | greenyellow |
| OR8D1 | greenyellow |
| OR8H3 | greenyellow |
| OR8J3 | greenyellow |
| OS9 | greenyellow |
| OSBPL1A | greenyellow |
| OSBPL5 | greenyellow |
| OSCP1 | greenyellow |
| OSGIN2 | greenyellow |
| P2RY6 | greenyellow |
| PADI4 | greenyellow |
| PALM2-AKAP2 | greenyellow |
| PANK4 | greenyellow |
| PANX3 | greenyellow |
| PAPD5 | greenyellow |
| PARP9 | greenyellow |
| PATE1 | greenyellow |
| PBEF1 | greenyellow |
| PCBP1 | greenyellow |
| PCCA | greenyellow |
| PCDH15 | greenyellow |
| PCDHA8 | greenyellow |
| PCDHB9 | greenyellow |
| PCDHGA1 | greenyellow |
| PCDHGB3 | greenyellow |
| PCNXL2 | greenyellow |
| PCSK9 | greenyellow |
| PDCL3 | greenyellow |
| PDE11A | greenyellow |
| PDE7A | greenyellow |
| PDRG1 | greenyellow |
| PDSS1 | greenyellow |
| PDZK3 | greenyellow |
| PELI2 | greenyellow |
| PEPD | greenyellow |
| PFDN4 | greenyellow |
| PGA3 | greenyellow |
| PGLS | greenyellow |
| PGS1 | greenyellow |
| PHF21B | greenyellow |
| PHF3 | greenyellow |
| PI3 | greenyellow |
| PI4KAP1 | greenyellow |
| PIGY | greenyellow |
| PIK3C2G | greenyellow |
| PIK3R5 | greenyellow |
| PIWIL3 | greenyellow |
| PKN1 | greenyellow |
| PLAGL2 | greenyellow |
| PLD1 | greenyellow |
| PLEKHF1 | greenyellow |
| PLEKHJ1 | greenyellow |
| PLK1 | greenyellow |
| PLOD3 | greenyellow |
| PLSCR5 | greenyellow |
| PMP2 | greenyellow |
| PMP22 | greenyellow |
| PMS2L2 | greenyellow |
| PNCK | greenyellow |
| PNMA2 | greenyellow |
| PNRC1 | greenyellow |
| POLB | greenyellow |
| POLG2 | greenyellow |
| POLR3D | greenyellow |
| POLRMT | greenyellow |
| POM121 | greenyellow |
| POMP | greenyellow |
| POP7 | greenyellow |
| PPA1 | greenyellow |
| PPAPDC2 | greenyellow |
| PPCS | greenyellow |
| PPHLN1 | greenyellow |
| PPIL6 | greenyellow |
| PPP2R5D | greenyellow |
| PPP3CC | greenyellow |
| PPP4R2 | greenyellow |
| PQBP1 | greenyellow |
| PRAMEF10 | greenyellow |
| PRAMEF9 | greenyellow |
| PRDM7 | greenyellow |
| PRDM9 | greenyellow |
| PRDX1 | greenyellow |
| PRDX3 | greenyellow |
| PRIC285 | greenyellow |
| PRKAR1B | greenyellow |
| PRKCA | greenyellow |
| PRKCZ | greenyellow |
| PRKG1 | greenyellow |
| PRR7 | greenyellow |
| PRSS35 | greenyellow |
| PSMC4 | greenyellow |
| PSMD13 | greenyellow |
| PSMD8 | greenyellow |
| PTCHD2 | greenyellow |
| PTGDR | greenyellow |
| PTPRB | greenyellow |
| PUS3 | greenyellow |
| PYCARD | greenyellow |
| QPCTL | greenyellow |
| QTRT1 | greenyellow |
| R3HCC1 | greenyellow |
| RAB32 | greenyellow |
| RAB40C | greenyellow |
| RAD51 | greenyellow |
| RAN | greenyellow |
| RAP1B | greenyellow |
| RAXL1 | greenyellow |
| RBBP4 | greenyellow |
| RBCK1 | greenyellow |
| RBM15 | greenyellow |
| RBM7 | greenyellow |
| RBMXL2 | greenyellow |
| RBPJL | greenyellow |
| RD3 | greenyellow |
| RDH12 | greenyellow |
| RDH5 | greenyellow |
| REEP5 | greenyellow |
| RGS12 | greenyellow |
| RHD | greenyellow |
| RHOA | greenyellow |
| RIF1 | greenyellow |
| RIOK3 | greenyellow |
| RND2 | greenyellow |
| RNF148 | greenyellow |
| RNF175 | greenyellow |
| RNPEP | greenyellow |
| ROD1 | greenyellow |
| RP1L1 | greenyellow |
| RPAP2 | greenyellow |
| RPL13A | greenyellow |
| RPL21 | greenyellow |
| RPL22L1 | greenyellow |
| RPL38 | greenyellow |
| RPL7L1 | greenyellow |
| RPP30 | greenyellow |
| RPS10 | greenyellow |
| RPS6KL1 | greenyellow |
| RRH | greenyellow |
| RRM2 | greenyellow |
| RSBN1 | greenyellow |
| RWDD3 | greenyellow |
| RXFP3 | greenyellow |
| S100A7A | greenyellow |
| SACS | greenyellow |
| SAMD1 | greenyellow |
| SAMD8 | greenyellow |
| SCAND3 | greenyellow |
| SCCPDH | greenyellow |
| SCN7A | greenyellow |
| SCUBE1 | greenyellow |
| SDC4 | greenyellow |
| SDCCAG10 | greenyellow |
| SEC11C | greenyellow |
| SEC24B | greenyellow |
| SEMA6B | greenyellow |
| SERP2 | greenyellow |
| SERPINB6 | greenyellow |
| SERPINH1 | greenyellow |
| SFRS9 | greenyellow |
| SGPL1 | greenyellow |
| SIK3 | greenyellow |
| SLC16A12 | greenyellow |
| SLC1A7 | greenyellow |
| SLC22A13 | greenyellow |
| SLC22A5 | greenyellow |
| SLC25A20 | greenyellow |
| SLC26A11 | greenyellow |
| SLC26A9 | greenyellow |
| SLC2A4 | greenyellow |
| SLC2A5 | greenyellow |
| SLC2A6 | greenyellow |
| SLC34A1 | greenyellow |
| SLC35B4 | greenyellow |
| SLC35E3 | greenyellow |
| SLC36A4 | greenyellow |
| SLC6A5 | greenyellow |
| SLC9A6 | greenyellow |
| SLFN12 | greenyellow |
| SLFNL1 | greenyellow |
| SLITRK6 | greenyellow |
| SMG5 | greenyellow |
| SMU1 | greenyellow |
| SNAI3 | greenyellow |
| SNAPC2 | greenyellow |
| SNIP | greenyellow |
| SNIP1 | greenyellow |
| SNORD14A | greenyellow |
| SNORD15A | greenyellow |
| SNORD22 | greenyellow |
| SNORD31 | greenyellow |
| SNORD48 | greenyellow |
| SNRPD1 | greenyellow |
| SNTA1 | greenyellow |
| SNX11 | greenyellow |
| SNX32 | greenyellow |
| SOHLH1 | greenyellow |
| SOSTDC1 | greenyellow |
| SP5 | greenyellow |
| SPACA3 | greenyellow |
| SPAG9 | greenyellow |
| SPATA16 | greenyellow |
| SPATA8 | greenyellow |
| SPEF2 | greenyellow |
| SPESP1 | greenyellow |
| SPNS3 | greenyellow |
| SPRN | greenyellow |
| SRFBP1 | greenyellow |
| SSBP3 | greenyellow |
| SSX5 | greenyellow |
| STARD8 | greenyellow |
| STBD1 | greenyellow |
| STK3 | greenyellow |
| STOX2 | greenyellow |
| STRAP | greenyellow |
| STX19 | greenyellow |
| SULT1C2 | greenyellow |
| SULT1C3 | greenyellow |
| SUZ12 | greenyellow |
| SV2C | greenyellow |
| SYAP1 | greenyellow |
| SYF2 | greenyellow |
| SYNGR3 | greenyellow |
| TAAR1 | greenyellow |
| TAAR9 | greenyellow |
| TACR1 | greenyellow |
| TAS2R31 | greenyellow |
| TAX1BP1 | greenyellow |
| TBC1D10A | greenyellow |
| TBC1D13 | greenyellow |
| TBC1D15 | greenyellow |
| TBC1D20 | greenyellow |
| TBC1D8B | greenyellow |
| TBP | greenyellow |
| TCEB3B | greenyellow |
| TCF19 | greenyellow |
| TCF7L1 | greenyellow |
| TCOF1 | greenyellow |
| TEAD3 | greenyellow |
| TERT | greenyellow |
| TFDP1 | greenyellow |
| TGOLN2 | greenyellow |
| THAP1 | greenyellow |
| THOP1 | greenyellow |
| THRB | greenyellow |
| THY1 | greenyellow |
| TIMP1 | greenyellow |
| TKTL1 | greenyellow |
| TM4SF1 | greenyellow |
| TM4SF5 | greenyellow |
| TMCC1 | greenyellow |
| TMED10 | greenyellow |
| TMED6 | greenyellow |
| TMEM102 | greenyellow |
| TMEM120A | greenyellow |
| TMEM180 | greenyellow |
| TMEM200C | greenyellow |
| TMEM50B | greenyellow |
| TMEM67 | greenyellow |
| TMEM83 | greenyellow |
| TMIE | greenyellow |
| TMOD4 | greenyellow |
| TMPRSS4 | greenyellow |
| TNFAIP8L1 | greenyellow |
| TNFRSF13B | greenyellow |
| TNFRSF1A | greenyellow |
| TOE1 | greenyellow |
| TOR1AIP2 | greenyellow |
| TP53TG5 | greenyellow |
| TPM3 | greenyellow |
| TPPP3 | greenyellow |
| TPRG1 | greenyellow |
| TRAF2 | greenyellow |
| TRAP1 | greenyellow |
| TRIM14 | greenyellow |
| TRIM8 | greenyellow |
| TRIP12 | greenyellow |
| TRMT12 | greenyellow |
| TRPC6 | greenyellow |
| TSHZ2 | greenyellow |
| TSPAN1 | greenyellow |
| TSPAN9 | greenyellow |
| TTC28 | greenyellow |
| TTC30A | greenyellow |
| TTC7A | greenyellow |
| TTLL3 | greenyellow |
| TTTY16 | greenyellow |
| TTTY3 | greenyellow |
| TTTY6B | greenyellow |
| TTTY7 | greenyellow |
| TUBB2B | greenyellow |
| TWF1 | greenyellow |
| TXNDC14 | greenyellow |
| UBE2M | greenyellow |
| UBE2Q1 | greenyellow |
| UBE4B | greenyellow |
| UBR4 | greenyellow |
| UBXN2A | greenyellow |
| UBXN8 | greenyellow |
| UCN | greenyellow |
| UFSP1 | greenyellow |
| UGCGL1 | greenyellow |
| UGCGL2 | greenyellow |
| UGT1A7 | greenyellow |
| UGT2B17 | greenyellow |
| UGT2B4 | greenyellow |
| UIMC1 | greenyellow |
| UQCRH | greenyellow |
| UQCRHL | greenyellow |
| VASH1 | greenyellow |
| VASP | greenyellow |
| VAV1 | greenyellow |
| VGLL1 | greenyellow |
| VGLL2 | greenyellow |
| VIT | greenyellow |
| VN1R4 | greenyellow |
| VPS37B | greenyellow |
| VTN | greenyellow |
| VWA3B | greenyellow |
| VWCE | greenyellow |
| WBP1 | greenyellow |
| WDR46 | greenyellow |
| WDTC1 | greenyellow |
| XAGE3 | greenyellow |
| XKR6 | greenyellow |
| XKRY | greenyellow |
| ZBTB26 | greenyellow |
| ZBTB3 | greenyellow |
| ZBTB4 | greenyellow |
| ZC3H15 | greenyellow |
| ZCCHC5 | greenyellow |
| ZCCHC6 | greenyellow |
| ZFAND2A | greenyellow |
| ZFAND3 | greenyellow |
| ZFP91 | greenyellow |
| ZFYVE20 | greenyellow |
| ZGPAT | greenyellow |
| ZMPSTE24 | greenyellow |
| ZMYND12 | greenyellow |
| ZNF143 | greenyellow |
| ZNF177 | greenyellow |
| ZNF181 | greenyellow |
| ZNF192 | greenyellow |
| ZNF2 | greenyellow |
| ZNF207 | greenyellow |
| ZNF274 | greenyellow |
| ZNF295 | greenyellow |
| ZNF331 | greenyellow |
| ZNF391 | greenyellow |
| ZNF44 | greenyellow |
| ZNF442 | greenyellow |
| ZNF45 | greenyellow |
| ZNF485 | greenyellow |
| ZNF517 | greenyellow |
| ZNF541 | greenyellow |
| ZNF567 | greenyellow |
| ZNF575 | greenyellow |
| ZNF605 | greenyellow |
| ZNF641 | greenyellow |
| ZNF670 | greenyellow |
| ZNF675 | greenyellow |
| ZNF774 | greenyellow |
| A3GALT2 | grey |
| ABHD8 | grey |
| ACOT1 | grey |
| ADAMTS12 | grey |
| ADRA1A | grey |
| AGAP2 | grey |
| AGBL3 | grey |
| AHSP | grey |
| AKAP3 | grey |
| ALDH18A1 | grey |
| ALDH3B2 | grey |
| ALKBH6 | grey |
| ANKRD22 | grey |
| APH1A | grey |
| APOC1 | grey |
| APPL2 | grey |
| ARNTL2 | grey |
| ATF7 | grey |
| ATP13A4 | grey |
| ATP8A1 | grey |
| ATRX | grey |
| B4GALNT3 | grey |
| BCDIN3D | grey |
| BCL2L2 | grey |
| BMP2KL | grey |
| BRD1 | grey |
| BTG2 | grey |
| C10orf120 | grey |
| C11orf66 | grey |
| C12orf43 | grey |
| C12orf53 | grey |
| C13orf35 | grey |
| C14orf125 | grey |
| C16orf91 | grey |
| C16orf93 | grey |
| C17orf102 | grey |
| C18orf22 | grey |
| C18orf55 | grey |
| C19orf24 | grey |
| C1orf105 | grey |
| C1orf190 | grey |
| C1orf216 | grey |
| C21orf42 | grey |
| C4BPA | grey |
| C4orf23 | grey |
| C4orf51 | grey |
| C8ORFK36 | grey |
| C8orf55 | grey |
| C9orf37 | grey |
| CASQ2 | grey |
| CCDC144NL | grey |
| CCDC42 | grey |
| CCDC45 | grey |
| CCDC5 | grey |
| CCDC71 | grey |
| CCL28 | grey |
| CDC14A | grey |
| CETP | grey |
| CFDP1 | grey |
| CFI | grey |
| CH25H | grey |
| CHCHD10 | grey |
| CHRNA7 | grey |
| CHST4 | grey |
| CIDEA | grey |
| CLDN16 | grey |
| CLDN20 | grey |
| CLEC1A | grey |
| CLMN | grey |
| CMBL | grey |
| CNGA4 | grey |
| CRCP | grey |
| CRELD2 | grey |
| CREM | grey |
| CSMD2 | grey |
| CSNK1G3 | grey |
| CST1 | grey |
| CUGBP2 | grey |
| CUTC | grey |
| DCAF12L2 | grey |
| DDX52 | grey |
| DIRAS3 | grey |
| DKFZp434N035 | grey |
| DKFZp686E2433 | grey |
| DNAH8 | grey |
| DNAJC12 | grey |
| DNMT3L | grey |
| DPYSL5 | grey |
| DRD1 | grey |
| EED | grey |
| EFNB3 | grey |
| EGFL6 | grey |
| ERAP2 | grey |
| ERP29 | grey |
| ETF1 | grey |
| FAM108C1 | grey |
| FAM128B | grey |
| FBXL6 | grey |
| FCGR3B | grey |
| FEM1B | grey |
| FGF7 | grey |
| FIBIN | grey |
| FIS | grey |
| FITM2 | grey |
| FKBP1B | grey |
| FLJ14186 | grey |
| FLJ27255 | grey |
| FLJ35801 | grey |
| FLJ44186 | grey |
| FLJ44450 | grey |
| FLJ46020 | grey |
| FOXN4 | grey |
| FPR3 | grey |
| FRMPD1 | grey |
| FRS3 | grey |
| GADL1 | grey |
| GFRA4 | grey |
| GIT2 | grey |
| GLRA3 | grey |
| GNA13 | grey |
| GP2 | grey |
| GP6 | grey |
| GPR107 | grey |
| GPR126 | grey |
| GPR137C | grey |
| GPR158L1 | grey |
| GPR172A | grey |
| GRM2 | grey |
| GRXCR1 | grey |
| GUCA1A | grey |
| HCG22 | grey |
| HDAC7A | grey |
| HLA-B | grey |
| HMGN4 | grey |
| HMOX2 | grey |
| HNRNPD | grey |
| HNRNPF | grey |
| HPGDS | grey |
| HRH3 | grey |
| HS6ST1 | grey |
| HSPC111 | grey |
| IDUA | grey |
| IER5L | grey |
| IFNA21 | grey |
| IL12RB2 | grey |
| ITGA1 | grey |
| ITPKC | grey |
| ITPRIPL2 | grey |
| IVL | grey |
| JCLN | grey |
| KIAA0194 | grey |
| KIAA0258 | grey |
| KIAA2013 | grey |
| KPNB1 | grey |
| KRIT1 | grey |
| KRT26 | grey |
| KRTAP4-11 | grey |
| KRTAP5-11 | grey |
| LELP1 | grey |
| LGR4 | grey |
| LILRB5 | grey |
| LIN7C | grey |
| LOC120376 | grey |
| LOC132203 | grey |
| LOC148766 | grey |
| LOC149224 | grey |
| LOC283152 | grey |
| LOC339862 | grey |
| LOC340156 | grey |
| LOC340598 | grey |
| LOC387895 | grey |
| LOC390688 | grey |
| LOC391730 | grey |
| LOC400163 | grey |
| LOC401218 | grey |
| LOC401237 | grey |
| LOC402665 | grey |
| LOC440132 | grey |
| LOC441151 | grey |
| LOC441212 | grey |
| LOC441440 | grey |
| LOC619207 | grey |
| LOC642558 | grey |
| LOC642644 | grey |
| LOC642662 | grey |
| LOC642782 | grey |
| LOC642788 | grey |
| LOC642830 | grey |
| LOC643060 | grey |
| LOC643081 | grey |
| LOC643150 | grey |
| LOC643331 | grey |
| LOC643409 | grey |
| LOC643747 | grey |
| LOC643808 | grey |
| LOC643872 | grey |
| LOC644039 | grey |
| LOC644122 | grey |
| LOC644402 | grey |
| LOC644457 | grey |
| LOC644756 | grey |
| LOC644885 | grey |
| LOC644891 | grey |
| LOC644978 | grey |
| LOC645013 | grey |
| LOC645777 | grey |
| LOC645932 | grey |
| LOC646154 | grey |
| LOC646236 | grey |
| LOC646237 | grey |
| LOC646400 | grey |
| LOC646615 | grey |
| LOC646706 | grey |
| LOC646716 | grey |
| LOC647328 | grey |
| LOC647543 | grey |
| LOC648223 | grey |
| LOC648434 | grey |
| LOC648612 | grey |
| LOC648738 | grey |
| LOC648966 | grey |
| LOC649107 | grey |
| LOC649279 | grey |
| LOC649452 | grey |
| LOC649991 | grey |
| LOC650076 | grey |
| LOC650111 | grey |
| LOC650212 | grey |
| LOC650436 | grey |
| LOC650933 | grey |
| LOC651131 | grey |
| LOC651213 | grey |
| LOC651286 | grey |
| LOC651493 | grey |
| LOC651758 | grey |
| LOC651859 | grey |
| LOC651876 | grey |
| LOC651974 | grey |
| LOC652183 | grey |
| LOC652282 | grey |
| LOC652688 | grey |
| LOC652701 | grey |
| LOC652813 | grey |
| LOC652849 | grey |
| LOC653182 | grey |
| LOC653217 | grey |
| LOC653286 | grey |
| LOC653380 | grey |
| LOC653515 | grey |
| LOC653866 | grey |
| LOC653884 | grey |
| LOC653983 | grey |
| LOC727832 | grey |
| LOC729218 | grey |
| LOC730083 | grey |
| LOC730358 | grey |
| LOC732387 | grey |
| LOC96610 | grey |
| LOXL2 | grey |
| LY6D | grey |
| MAN1B1 | grey |
| MAN1C1 | grey |
| MAP7 | grey |
| MAPK15 | grey |
| MAPK7 | grey |
| MAPRE1 | grey |
| MDS1 | grey |
| MFSD1 | grey |
| MGC71805 | grey |
| MOG | grey |
| MPZL2 | grey |
| MRPS21 | grey |
| MRPS9 | grey |
| MSH3 | grey |
| MSL3L2 | grey |
| MUPCDH | grey |
| NECAB2 | grey |
| NEFM | grey |
| NKX2-5 | grey |
| NLRP6 | grey |
| NMT1 | grey |
| NPM1 | grey |
| NPM3 | grey |
| NR2C2 | grey |
| NR2F1 | grey |
| NR2F2 | grey |
| NRAP | grey |
| NUP133 | grey |
| NUSAP1 | grey |
| OBSCN | grey |
| OMG | grey |
| OR2A5 | grey |
| OR2W1 | grey |
| OR52A1 | grey |
| OR52E2 | grey |
| OR52N4 | grey |
| OR5B12 | grey |
| OR5R1 | grey |
| OR6C2 | grey |
| OR7G3 | grey |
| P2RY13 | grey |
| PAGE2B | grey |
| PAGE4 | grey |
| PANX1 | grey |
| PARN | grey |
| PCSK1 | grey |
| PECR | grey |
| PGCP | grey |
| PGLYRP3 | grey |
| PGM5 | grey |
| PIAS4 | grey |
| PITRM1 | grey |
| PITX1 | grey |
| PLCB3 | grey |
| PLSCR3 | grey |
| PMS1 | grey |
| POLL | grey |
| POLR2A | grey |
| POP4 | grey |
| PPL | grey |
| PRR11 | grey |
| PSMC5 | grey |
| PSME3 | grey |
| PTPN20B | grey |
| PTPRT | grey |
| RAB33B | grey |
| RGS19 | grey |
| RHCG | grey |
| RHOH | grey |
| RIMS1 | grey |
| RNF167 | grey |
| ROBO3 | grey |
| RPL14 | grey |
| RPL23 | grey |
| RSU1 | grey |
| RUFY4 | grey |
| S1PR5 | grey |
| SAMD12 | grey |
| SEMA3E | grey |
| SERPINE2 | grey |
| SETDB2 | grey |
| SF3B5 | grey |
| SFRS6 | grey |
| SFTPA2 | grey |
| SH3BP1 | grey |
| SIX6 | grey |
| SLC22A16 | grey |
| SLC2A12 | grey |
| SLC35D3 | grey |
| SLC38A7 | grey |
| SLC6A4 | grey |
| SMARCC1 | grey |
| SMO | grey |
| SMOC1 | grey |
| SNORD55 | grey |
| SOX30 | grey |
| SPACA5 | grey |
| SPATA3 | grey |
| SPSB4 | grey |
| STK35 | grey |
| STRA13 | grey |
| STS-1 | grey |
| TAC3 | grey |
| TAOK1 | grey |
| TAP2 | grey |
| TARSL2 | grey |
| TCP11 | grey |
| TDGF3 | grey |
| TFPT | grey |
| TFRC | grey |
| THBD | grey |
| THBS1 | grey |
| THRAP3 | grey |
| TICAM1 | grey |
| TIMM44 | grey |
| TM4SF4 | grey |
| TMEM8 | grey |
| TMPRSS7 | grey |
| TNFSF12 | grey |
| TNP1 | grey |
| TNP2 | grey |
| TNS1 | grey |
| TOMM5 | grey |
| TPSG1 | grey |
| TRAPPC1 | grey |
| TRHDE | grey |
| TRIB3 | grey |
| TRIM52 | grey |
| TRPM7 | grey |
| TRPV4 | grey |
| TTC13 | grey |
| TTL | grey |
| TTLL4 | grey |
| TTTY13 | grey |
| TUBB1 | grey |
| TUSC5 | grey |
| TYMS | grey |
| UAP1 | grey |
| UBAP2L | grey |
| UBE2C | grey |
| UBXN7 | grey |
| UGT2B28 | grey |
| UNCX | grey |
| UROC1 | grey |
| USO1 | grey |
| UTP11L | grey |
| VPS25 | grey |
| WDYHV1 | grey |
| WFDC12 | grey |
| WFDC2 | grey |
| YKT6 | grey |
| ZC3H18 | grey |
| ZEB1 | grey |
| ZFATAS | grey |
| ZNF214 | grey |
| ZNF273 | grey |
| ZNF335 | grey |
| ZNF346 | grey |
| ZNF385A | grey |
| ZNF414 | grey |
| ZNF501 | grey |
| ZNF528 | grey |
| ZNF582 | grey |
| ZNF714 | grey |
| ZNF750 | grey |
| ZNF763 | grey |
| ZNF780B | grey |
| ZNF781 | grey |
| ABL1 | midnightblue |
| AP3M1 | midnightblue |
| AQP6 | midnightblue |
| ATXN7L3 | midnightblue |
| BTBD9 | midnightblue |
| C14orf145 | midnightblue |
| C1orf166 | midnightblue |
| C20orf86 | midnightblue |
| C3orf31 | midnightblue |
| CHAF1B | midnightblue |
| CHMP1B | midnightblue |
| DBX2 | midnightblue |
| DEFB112 | midnightblue |
| DOCK8 | midnightblue |
| ELAC2 | midnightblue |
| ENTPD6 | midnightblue |
| FADS1 | midnightblue |
| FADS3 | midnightblue |
| FAM10A4 | midnightblue |
| FANK1 | midnightblue |
| FETUB | midnightblue |
| FHL1 | midnightblue |
| FLJ22222 | midnightblue |
| FLJ30375 | midnightblue |
| FLJ40244 | midnightblue |
| GPSM2 | midnightblue |
| GRID2 | midnightblue |
| HDHD2 | midnightblue |
| HMGCS2 | midnightblue |
| ICOS | midnightblue |
| ING4 | midnightblue |
| LOC389834 | midnightblue |
| LOC641522 | midnightblue |
| LOC641694 | midnightblue |
| LOC644500 | midnightblue |
| LOC644628 | midnightblue |
| LOC644763 | midnightblue |
| LOC644783 | midnightblue |
| LOC645045 | midnightblue |
| LOC645307 | midnightblue |
| LOC645520 | midnightblue |
| LOC645963 | midnightblue |
| LOC651293 | midnightblue |
| LOC651468 | midnightblue |
| LOC651746 | midnightblue |
| LOC652628 | midnightblue |
| LOC652707 | midnightblue |
| LOC652886 | midnightblue |
| LOC652887 | midnightblue |
| LOC653618 | midnightblue |
| LOC727992 | midnightblue |
| LOC731408 | midnightblue |
| LRRC18 | midnightblue |
| MERTK | midnightblue |
| MGC44328 | midnightblue |
| NOP10 | midnightblue |
| OR2D3 | midnightblue |
| OTOR | midnightblue |
| PAEP | midnightblue |
| PAK7 | midnightblue |
| PAX5 | midnightblue |
| PCDHGB1 | midnightblue |
| PCDHGB8P | midnightblue |
| PCYT1B | midnightblue |
| PEX19 | midnightblue |
| POTEF | midnightblue |
| PPP1R13B | midnightblue |
| PURA | midnightblue |
| RBP3 | midnightblue |
| RELN | midnightblue |
| RIC8B | midnightblue |
| RNF19B | midnightblue |
| RNF31 | midnightblue |
| RPS20 | midnightblue |
| RPS6KA4 | midnightblue |
| RUNDC3A | midnightblue |
| RXRB | midnightblue |
| SORCS1 | midnightblue |
| TFAP2D | midnightblue |
| TGM5 | midnightblue |
| TRAF4 | midnightblue |
| TULP1 | midnightblue |
| USP20 | midnightblue |
| VDAC1 | midnightblue |
| VMAC | midnightblue |
| ZFAT | midnightblue |
| ZNF778 | midnightblue |
| ABP1 | pink |
| ACSS1 | pink |
| ADAM21 | pink |
| ANP32A | pink |
| ARL14 | pink |
| ARL6 | pink |
| B3GNT9 | pink |
| BARD1 | pink |
| C11orf34 | pink |
| C11orf70 | pink |
| C11orf87 | pink |
| C14orf142 | pink |
| C14orf156 | pink |
| C16orf57 | pink |
| C16orf71 | pink |
| C17orf76 | pink |
| C18orf8 | pink |
| C1orf122 | pink |
| C1orf135 | pink |
| C21orf122 | pink |
| C3orf65 | pink |
| C4orf26 | pink |
| C6orf191 | pink |
| C7orf16 | pink |
| C7orf45 | pink |
| C8orf86 | pink |
| CABYR | pink |
| CACNA1S | pink |
| CCRN4L | pink |
| CENTA1 | pink |
| CHCHD9 | pink |
| CHP | pink |
| CIDEC | pink |
| COX10 | pink |
| CPEB3 | pink |
| CREB3L2 | pink |
| CRSP6 | pink |
| CSMD1 | pink |
| DNAJB9 | pink |
| DSCR9 | pink |
| EIF2AK3 | pink |
| ENKUR | pink |
| EPAS1 | pink |
| ETHE1 | pink |
| ETNK2 | pink |
| FAM134C | pink |
| FAM43B | pink |
| FAM55B | pink |
| FAM63A | pink |
| FIZ1 | pink |
| FLJ32682 | pink |
| FOXC2 | pink |
| FOXN1 | pink |
| FXYD7 | pink |
| GJA10 | pink |
| GLCCI1 | pink |
| GOSR2 | pink |
| H1FOO | pink |
| HCP5 | pink |
| HDDC2 | pink |
| HECA | pink |
| HGS | pink |
| HIST1H4J | pink |
| HLA-DPB2 | pink |
| HOXD1 | pink |
| HSPA1A | pink |
| ICAM3 | pink |
| IFI35 | pink |
| IFITM1 | pink |
| ILDR1 | pink |
| IMPA1 | pink |
| KCNJ13 | pink |
| KIAA1160 | pink |
| KLHDC3 | pink |
| KLHL30 | pink |
| KRT74 | pink |
| KRTAP10-1 | pink |
| LHX8 | pink |
| LMX1B | pink |
| LOC149069 | pink |
| LOC388743 | pink |
| LOC390594 | pink |
| LOC402217 | pink |
| LOC440956 | pink |
| LOC442406 | pink |
| LOC641741 | pink |
| LOC641742 | pink |
| LOC642031 | pink |
| LOC642072 | pink |
| LOC642756 | pink |
| LOC643438 | pink |
| LOC643692 | pink |
| LOC644135 | pink |
| LOC644574 | pink |
| LOC644929 | pink |
| LOC645139 | pink |
| LOC645144 | pink |
| LOC645451 | pink |
| LOC646132 | pink |
| LOC646484 | pink |
| LOC646531 | pink |
| LOC647624 | pink |
| LOC647890 | pink |
| LOC648354 | pink |
| LOC649470 | pink |
| LOC650706 | pink |
| LOC651169 | pink |
| LOC651848 | pink |
| LOC652286 | pink |
| LOC652492 | pink |
| LOC652589 | pink |
| LOC652624 | pink |
| LOC653253 | pink |
| LOC653492 | pink |
| LOC727937 | pink |
| LOC728493 | pink |
| LOC730092 | pink |
| LRRC37A4 | pink |
| MED16 | pink |
| METT10D | pink |
| MGC15705 | pink |
| MGC29506 | pink |
| MGC42105 | pink |
| MGC42630 | pink |
| MPP1 | pink |
| MS4A2 | pink |
| MTFR1 | pink |
| MTTP | pink |
| MUS81 | pink |
| MYO7B | pink |
| NCBP2 | pink |
| NEK1 | pink |
| NR1D1 | pink |
| NR4A1 | pink |
| NRM | pink |
| NUDT6 | pink |
| NUDT9P1 | pink |
| NUP205 | pink |
| NUP62 | pink |
| NUP85 | pink |
| NXF2 | pink |
| OR2T8 | pink |
| OR4D6 | pink |
| ORAI1 | pink |
| PDE9A | pink |
| PEF1 | pink |
| PENK | pink |
| PITPNB | pink |
| PNRC2 | pink |
| PPP2R2A | pink |
| PRAMEF17 | pink |
| PRB2 | pink |
| PRB4 | pink |
| PRKCE | pink |
| PRKY | pink |
| PRSS33 | pink |
| PTS | pink |
| PYGL | pink |
| ProSAPiP1 | pink |
| QRFP | pink |
| REEP2 | pink |
| RPL35 | pink |
| RPS26P11 | pink |
| RS1 | pink |
| RUNDC3B | pink |
| RWDD2B | pink |
| S100A6 | pink |
| SAR1A | pink |
| SCYL3 | pink |
| SEC16A | pink |
| SIVA | pink |
| SLC2A8 | pink |
| SLC4A1 | pink |
| SLC7A10 | pink |
| SMR3B | pink |
| SNORD43 | pink |
| SQLE | pink |
| SSTR5 | pink |
| ST6GALNAC6 | pink |
| STAR | pink |
| STON1-GTF2A1L | pink |
| TARBP1 | pink |
| TMC6 | pink |
| TMEM63B | pink |
| TOP3A | pink |
| TPCN1 | pink |
| TRIB2 | pink |
| TSPAN16 | pink |
| UGT1A3 | pink |
| WDR44 | pink |
| WNT2B | pink |
| XIST | pink |
| ZNF497 | pink |
| A26C3 | purple |
| AARSD1 | purple |
| ABCB5 | purple |
| ABI3 | purple |
| ABLIM3 | purple |
| ACCN3 | purple |
| ACOT2 | purple |
| ACOT7 | purple |
| ACTL7B | purple |
| ADAM18 | purple |
| ADAM8 | purple |
| ADAMTS15 | purple |
| ADAMTS19 | purple |
| ADAMTS6 | purple |
| ADAR | purple |
| ADCK1 | purple |
| ADPRHL1 | purple |
| ADRA2C | purple |
| ADSS | purple |
| AFG3L1 | purple |
| AGBL1 | purple |
| AGK | purple |
| AGXT | purple |
| AIRE | purple |
| AKR1C1 | purple |
| AKT1S1 | purple |
| ALDH3A1 | purple |
| ALG13 | purple |
| ALG9 | purple |
| ALOX15B | purple |
| ALPP | purple |
| AMBRA1 | purple |
| AMELX | purple |
| AMICA1 | purple |
| ANGPT2 | purple |
| ANKRD13C | purple |
| ANKRD23 | purple |
| ANKRD43 | purple |
| ANKRD53 | purple |
| ANUBL1 | purple |
| AP4E1 | purple |
| AP4M1 | purple |
| AP4S1 | purple |
| APBB2 | purple |
| APOA4 | purple |
| APOBEC3A | purple |
| APOBEC3H | purple |
| AQP4 | purple |
| ARG2 | purple |
| ARHGAP15 | purple |
| ARHGAP22 | purple |
| ARHGEF12 | purple |
| ARHGEF6 | purple |
| ARL4C | purple |
| ARMCX4 | purple |
| ARPC1B | purple |
| ARSF | purple |
| ARVP6125 | purple |
| ASB16 | purple |
| ASB4 | purple |
| ASNS | purple |
| ATF6 | purple |
| ATG2B | purple |
| ATHL1 | purple |
| ATP10D | purple |
| ATP6AP1L | purple |
| AVL9 | purple |
| AXL | purple |
| AZI2 | purple |
| BAG3 | purple |
| BAGE5 | purple |
| BANF1 | purple |
| BARX1 | purple |
| BBS10 | purple |
| BCAP31 | purple |
| BCAT2 | purple |
| BCL8 | purple |
| BEX1 | purple |
| BEX4 | purple |
| BMP10 | purple |
| BMP5 | purple |
| BMPR1B | purple |
| BMPR2 | purple |
| BNIP3 | purple |
| BPIL2 | purple |
| BRD8 | purple |
| BRDG1 | purple |
| BRF1 | purple |
| BTC | purple |
| BTF3L4 | purple |
| BTG1 | purple |
| BTN2A1 | purple |
| BTNL8 | purple |
| C10orf26 | purple |
| C10orf28 | purple |
| C10orf41 | purple |
| C10orf46 | purple |
| C10orf62 | purple |
| C11orf71 | purple |
| C11orf88 | purple |
| C12orf23 | purple |
| C12orf44 | purple |
| C12orf49 | purple |
| C13orf18 | purple |
| C14orf148 | purple |
| C14orf162 | purple |
| C14orf184 | purple |
| C14orf68 | purple |
| C14orf72 | purple |
| C14orf82 | purple |
| C15orf29 | purple |
| C15orf41 | purple |
| C16orf48 | purple |
| C16orf55 | purple |
| C16orf69 | purple |
| C17orf47 | purple |
| C17orf55 | purple |
| C17orf70 | purple |
| C19orf18 | purple |
| C19orf23 | purple |
| C19orf25 | purple |
| C19orf36 | purple |
| C1D | purple |
| C1QL3 | purple |
| C1orf101 | purple |
| C1orf115 | purple |
| C1orf128 | purple |
| C1orf14 | purple |
| C1orf182 | purple |
| C1orf19 | purple |
| C1orf210 | purple |
| C1orf218 | purple |
| C1orf227 | purple |
| C1orf24 | purple |
| C1orf63 | purple |
| C1orf64 | purple |
| C1orf83 | purple |
| C20orf103 | purple |
| C20orf166 | purple |
| C20orf191 | purple |
| C20orf196 | purple |
| C20orf29 | purple |
| C20orf38 | purple |
| C20orf71 | purple |
| C21orf100 | purple |
| C21orf121 | purple |
| C21orf66 | purple |
| C22orf13 | purple |
| C22orf31 | purple |
| C2orf40 | purple |
| C2orf83 | purple |
| C3orf32 | purple |
| C3orf35 | purple |
| C3orf38 | purple |
| C3orf59 | purple |
| C3orf75 | purple |
| C4orf29 | purple |
| C4orf35 | purple |
| C4orf37 | purple |
| C4orf49 | purple |
| C4orf6 | purple |
| C5orf5 | purple |
| C6orf167 | purple |
| C6orf184 | purple |
| C6orf199 | purple |
| C6orf208 | purple |
| C7orf23 | purple |
| C7orf43 | purple |
| C8G | purple |
| C8orf30A | purple |
| C9orf110 | purple |
| C9orf14 | purple |
| C9orf144 | purple |
| C9orf41 | purple |
| C9orf68 | purple |
| C9orf71 | purple |
| C9orf84 | purple |
| CA9 | purple |
| CACNA2D3 | purple |
| CADM2 | purple |
| CALCR | purple |
| CAMK2N2 | purple |
| CAMK4 | purple |
| CAMKK1 | purple |
| CAMSAP1 | purple |
| CAMTA1 | purple |
| CARM1 | purple |
| CARTPT | purple |
| CCDC11 | purple |
| CCDC138 | purple |
| CCDC140 | purple |
| CCDC46 | purple |
| CCDC63 | purple |
| CCDC66 | purple |
| CCDC80 | purple |
| CCDC97 | purple |
| CCL13 | purple |
| CCL16 | purple |
| CCL24 | purple |
| CCL7 | purple |
| CCR4 | purple |
| CCR7 | purple |
| CD160 | purple |
| CD22 | purple |
| CD300C | purple |
| CD33 | purple |
| CD96 | purple |
| CDC2L6 | purple |
| CDC42EP3 | purple |
| CDH9 | purple |
| CDK7 | purple |
| CDON | purple |
| CDX1 | purple |
| CEACAM6 | purple |
| CECR6 | purple |
| CENPC1 | purple |
| CENPK | purple |
| CEP135 | purple |
| CEP76 | purple |
| CES2 | purple |
| CFC1 | purple |
| CFHR1 | purple |
| CHCHD3 | purple |
| CHD6 | purple |
| CHDH | purple |
| CHKA | purple |
| CHM | purple |
| CHMP1A | purple |
| CHRNA6 | purple |
| CIAPIN1 | purple |
| CICE | purple |
| CICK0721Q.1 | purple |
| CITED2 | purple |
| CKMT1A | purple |
| CLEC2A | purple |
| CLEC4F | purple |
| CLN5 | purple |
| CNGB3 | purple |
| CNKSR1 | purple |
| CNKSR2 | purple |
| CNTNAP1 | purple |
| CNTNAP3B | purple |
| COL9A3 | purple |
| COMMD3 | purple |
| COMTD1 | purple |
| COPS7B | purple |
| COPS8 | purple |
| COQ9 | purple |
| CORO1C | purple |
| COX5B | purple |
| COX6A1 | purple |
| COX6B1 | purple |
| COX7C | purple |
| CPT1A | purple |
| CPT1C | purple |
| CREBZF | purple |
| CRISP3 | purple |
| CRYBA1 | purple |
| CRYZL1 | purple |
| CSDA | purple |
| CSNK2A2 | purple |
| CST8 | purple |
| CTU2 | purple |
| CUL2 | purple |
| CUL4B | purple |
| CXorf1 | purple |
| CXorf22 | purple |
| CXorf24 | purple |
| CXorf41 | purple |
| CXorf48 | purple |
| CYLC2 | purple |
| CYP51A1 | purple |
| CYP7A1 | purple |
| CYP7B1 | purple |
| CYP8B1 | purple |
| DAK | purple |
| DAPK1 | purple |
| DBI | purple |
| DBNDD2 | purple |
| DCBLD1 | purple |
| DCDC5 | purple |
| DCHS2 | purple |
| DCST2 | purple |
| DCUN1D3 | purple |
| DDX59 | purple |
| DEFB1 | purple |
| DEFB103B | purple |
| DEFB121 | purple |
| DEFB137 | purple |
| DEFB32 | purple |
| DFNA5 | purple |
| DGAT2L3 | purple |
| DGKB | purple |
| DHFRL1 | purple |
| DIMT1L | purple |
| DISC1 | purple |
| DKFZp434I1020 | purple |
| DKFZp686J0529 | purple |
| DKFZp781N1041 | purple |
| DMRTC2 | purple |
| DNAH3 | purple |
| DNAHL1 | purple |
| DNAI2 | purple |
| DNAJC2 | purple |
| DNAJC9 | purple |
| DNLZ | purple |
| DNM1 | purple |
| DNMBP | purple |
| DNMT1 | purple |
| DOHH | purple |
| DOK3 | purple |
| DOPEY2 | purple |
| DPT | purple |
| DRP2 | purple |
| DSN1 | purple |
| DSPP | purple |
| DULLARD | purple |
| DUX2 | purple |
| ECHDC1 | purple |
| EDC3 | purple |
| EFCAB3 | purple |
| EGFL8 | purple |
| EIF4A2 | purple |
| EIF4B | purple |
| EIF4EBP1 | purple |
| ENAM | purple |
| EPHA1 | purple |
| ERCC6 | purple |
| ERGIC2 | purple |
| ERH | purple |
| ESF1 | purple |
| EVI2A | purple |
| EVL | purple |
| F2RL2 | purple |
| F9 | purple |
| FABP3 | purple |
| FABP9 | purple |
| FADD | purple |
| FADS6 | purple |
| FAM118A | purple |
| FAM118B | purple |
| FAM153C | purple |
| FAM154B | purple |
| FAM22B | purple |
| FAM26C | purple |
| FAM49A | purple |
| FAM55A | purple |
| FAM76A | purple |
| FARS2 | purple |
| FBN3 | purple |
| FBXL19 | purple |
| FBXO48 | purple |
| FBXW9 | purple |
| FGD6 | purple |
| FGF19 | purple |
| FGF2 | purple |
| FHOD1 | purple |
| FHOD3 | purple |
| FIP1L1 | purple |
| FKBP6 | purple |
| FKSG2 | purple |
| FKSG83 | purple |
| FLAD1 | purple |
| FLI1 | purple |
| FLII | purple |
| FLJ00312 | purple |
| FLJ14213 | purple |
| FLJ16793 | purple |
| FLJ20209 | purple |
| FLJ20297 | purple |
| FLJ20444 | purple |
| FLJ22531 | purple |
| FLJ22795 | purple |
| FLJ25076 | purple |
| FLJ27354 | purple |
| FLJ31945 | purple |
| FLJ32065 | purple |
| FLJ32255 | purple |
| FLJ33387 | purple |
| FLJ33996 | purple |
| FLJ34503 | purple |
| FLJ34870 | purple |
| FLJ35880 | purple |
| FLJ36031 | purple |
| FLJ40722 | purple |
| FLJ41200 | purple |
| FLJ42986 | purple |
| FLJ44216 | purple |
| FLJ45337 | purple |
| FLJ45721 | purple |
| FLJ45994 | purple |
| FLJ46210 | purple |
| FLJ46284 | purple |
| FLJ90036 | purple |
| FLRT3 | purple |
| FMNL1 | purple |
| FNDC1 | purple |
| FNDC3A | purple |
| FOLR2 | purple |
| FOXD2 | purple |
| FOXD4L2 | purple |
| FOXG1 | purple |
| FOXR2 | purple |
| FREM1 | purple |
| FREM3 | purple |
| FSHR | purple |
| FXYD6 | purple |
| G3BP2 | purple |
| GABBR2 | purple |
| GAGE2C | purple |
| GALM | purple |
| GARNL4 | purple |
| GAS1 | purple |
| GATC | purple |
| GATM | purple |
| GFRA3 | purple |
| GGCT | purple |
| GGCX | purple |
| GGNBP2 | purple |
| GIT1 | purple |
| GLIPR2 | purple |
| GLIS2 | purple |
| GLOD5 | purple |
| GLRX | purple |
| GLUD1 | purple |
| GLYATL2 | purple |
| GMCL1 | purple |
| GNA12 | purple |
| GNB1 | purple |
| GNG10 | purple |
| GNG5 | purple |
| GOLGA5 | purple |
| GON4L | purple |
| GPC5 | purple |
| GPN3 | purple |
| GPNMB | purple |
| GPR125 | purple |
| GPR144 | purple |
| GPR148 | purple |
| GPR182 | purple |
| GPR25 | purple |
| GPR68 | purple |
| GPR77 | purple |
| GPR82 | purple |
| GPX3 | purple |
| GRASP | purple |
| GREB1 | purple |
| GRPEL2 | purple |
| GRXCR2 | purple |
| GSPT1 | purple |
| GSTA1 | purple |
| GSTT1 | purple |
| GTDC1 | purple |
| GTF2A2 | purple |
| GTF2B | purple |
| GTF2E2 | purple |
| GUCY2F | purple |
| GYLTL1B | purple |
| GYS2 | purple |
| H1F0 | purple |
| H2AFY2 | purple |
| HADHB | purple |
| HAT1 | purple |
| HAUS8 | purple |
| HBB | purple |
| HBEGF | purple |
| HCG4P6 | purple |
| HCN1 | purple |
| HCRT | purple |
| HDGFL1 | purple |
| HELQ | purple |
| HES6 | purple |
| HEXIM2 | purple |
| HHATL | purple |
| HIGD1A | purple |
| HIPK3 | purple |
| HIRIP3 | purple |
| HIST1H2AH | purple |
| HIST1H2BF | purple |
| HIST1H3I | purple |
| HIST1H4B | purple |
| HIST2H2BF | purple |
| HK1 | purple |
| HNRPC | purple |
| HNRPR | purple |
| HOXA13 | purple |
| HOXB5 | purple |
| HRASLS3 | purple |
| HS3ST1 | purple |
| HS3ST6 | purple |
| HSBP1 | purple |
| HSF2BP | purple |
| HYI | purple |
| IDE | purple |
| IFIT3 | purple |
| IFT140 | purple |
| IFT74 | purple |
| IGF2R | purple |
| IGFBP2 | purple |
| IL11 | purple |
| IL12A | purple |
| IL13 | purple |
| IL1F6 | purple |
| IL1F8 | purple |
| IL21R | purple |
| IL26 | purple |
| IL28B | purple |
| ILDR2 | purple |
| INA | purple |
| INPP5B | purple |
| INSIG2 | purple |
| INSM2 | purple |
| INSR | purple |
| INTS7 | purple |
| IP6K2 | purple |
| IQCC | purple |
| IQCG | purple |
| IRAK1BP1 | purple |
| IRAK3 | purple |
| IRF6 | purple |
| IRS4 | purple |
| IRX6 | purple |
| ISG20 | purple |
| ITFG3 | purple |
| ITGA10 | purple |
| ITGB1BP1 | purple |
| ITGB3BP | purple |
| ITGB4BP | purple |
| ITGB8 | purple |
| ITK | purple |
| ITPKA | purple |
| JAK1 | purple |
| JAZF1 | purple |
| KAT2A | purple |
| KCNA7 | purple |
| KCND2 | purple |
| KCNJ11 | purple |
| KCTD7 | purple |
| KHDC1 | purple |
| KIAA0195 | purple |
| KIAA0460 | purple |
| KIAA0495 | purple |
| KIAA0556 | purple |
| KIAA0889 | purple |
| KIAA0913 | purple |
| KIAA1161 | purple |
| KIAA1210 | purple |
| KIAA1529 | purple |
| KIAA1539 | purple |
| KIAA1683 | purple |
| KIAA1843 | purple |
| KIAA1881 | purple |
| KIAA1919 | purple |
| KIAA2022 | purple |
| KLF9 | purple |
| KLHL10 | purple |
| KLHL18 | purple |
| KLHL34 | purple |
| KLRB1 | purple |
| KPNA3 | purple |
| KRT1 | purple |
| KRT27 | purple |
| KRT71 | purple |
| KRT80 | purple |
| KRT82 | purple |
| KRTAP4-8 | purple |
| KRTAP5-6 | purple |
| KRTAP6-1 | purple |
| KRTHB6 | purple |
| LARP4 | purple |
| LARS | purple |
| LAT2 | purple |
| LBXCOR1 | purple |
| LCA5 | purple |
| LCE1E | purple |
| LCP1 | purple |
| LDB1 | purple |
| LDHD | purple |
| LEFTY1 | purple |
| LEKR1 | purple |
| LEPR | purple |
| LEPREL2 | purple |
| LGALS3 | purple |
| LHX3 | purple |
| LHX4 | purple |
| LHX9 | purple |
| LIAS | purple |
| LILRA2 | purple |
| LILRP2 | purple |
| LMF1 | purple |
| LMF2 | purple |
| LOC100008589 | purple |
| LOC113386 | purple |
| LOC126536 | purple |
| LOC127295 | purple |
| LOC130678 | purple |
| LOC134121 | purple |
| LOC136143 | purple |
| LOC139363 | purple |
| LOC148915 | purple |
| LOC197350 | purple |
| LOC201175 | purple |
| LOC220729 | purple |
| LOC254028 | purple |
| LOC283050 | purple |
| LOC283174 | purple |
| LOC283398 | purple |
| LOC284064 | purple |
| LOC284067 | purple |
| LOC284672 | purple |
| LOC285033 | purple |
| LOC285047 | purple |
| LOC285359 | purple |
| LOC285735 | purple |
| LOC286260 | purple |
| LOC286528 | purple |
| LOC339766 | purple |
| LOC343296 | purple |
| LOC344875 | purple |
| LOC348840 | purple |
| LOC375748 | purple |
| LOC387693 | purple |
| LOC387804 | purple |
| LOC387939 | purple |
| LOC388117 | purple |
| LOC388242 | purple |
| LOC388312 | purple |
| LOC388681 | purple |
| LOC388885 | purple |
| LOC388906 | purple |
| LOC388931 | purple |
| LOC388955 | purple |
| LOC388969 | purple |
| LOC389072 | purple |
| LOC389203 | purple |
| LOC389267 | purple |
| LOC389396 | purple |
| LOC389748 | purple |
| LOC389786 | purple |
| LOC389983 | purple |
| LOC390231 | purple |
| LOC390570 | purple |
| LOC390956 | purple |
| LOC391004 | purple |
| LOC391378 | purple |
| LOC391692 | purple |
| LOC391722 | purple |
| LOC391766 | purple |
| LOC391771 | purple |
| LOC392447 | purple |
| LOC392559 | purple |
| LOC392617 | purple |
| LOC399900 | purple |
| LOC400388 | purple |
| LOC400721 | purple |
| LOC400890 | purple |
| LOC400963 | purple |
| LOC400986 | purple |
| LOC401072 | purple |
| LOC401101 | purple |
| LOC401137 | purple |
| LOC401233 | purple |
| LOC401497 | purple |
| LOC401498 | purple |
| LOC401623 | purple |
| LOC401629 | purple |
| LOC402232 | purple |
| LOC402269 | purple |
| LOC440268 | purple |
| LOC440341 | purple |
| LOC440518 | purple |
| LOC440896 | purple |
| LOC441135 | purple |
| LOC441239 | purple |
| LOC441296 | purple |
| LOC441347 | purple |
| LOC441376 | purple |
| LOC441426 | purple |
| LOC442020 | purple |
| LOC442057 | purple |
| LOC442060 | purple |
| LOC442147 | purple |
| LOC442206 | purple |
| LOC554223 | purple |
| LOC613266 | purple |
| LOC641743 | purple |
| LOC641788 | purple |
| LOC641805 | purple |
| LOC641848 | purple |
| LOC641950 | purple |
| LOC642037 | purple |
| LOC642082 | purple |
| LOC642105 | purple |
| LOC642210 | purple |
| LOC642219 | purple |
| LOC642373 | purple |
| LOC642434 | purple |
| LOC642443 | purple |
| LOC642451 | purple |
| LOC642528 | purple |
| LOC642537 | purple |
| LOC642561 | purple |
| LOC642673 | purple |
| LOC642684 | purple |
| LOC642712 | purple |
| LOC642716 | purple |
| LOC642762 | purple |
| LOC642772 | purple |
| LOC642785 | purple |
| LOC642804 | purple |
| LOC642833 | purple |
| LOC642855 | purple |
| LOC642866 | purple |
| LOC642891 | purple |
| LOC642934 | purple |
| LOC642948 | purple |
| LOC642954 | purple |
| LOC642961 | purple |
| LOC643006 | purple |
| LOC643011 | purple |
| LOC643082 | purple |
| LOC643102 | purple |
| LOC643210 | purple |
| LOC643236 | purple |
| LOC643320 | purple |
| LOC643325 | purple |
| LOC643327 | purple |
| LOC643373 | purple |
| LOC643428 | purple |
| LOC643441 | purple |
| LOC643580 | purple |
| LOC643584 | purple |
| LOC643623 | purple |
| LOC643626 | purple |
| LOC643719 | purple |
| LOC643772 | purple |
| LOC643801 | purple |
| LOC643811 | purple |
| LOC643866 | purple |
| LOC643883 | purple |
| LOC643884 | purple |
| LOC643906 | purple |
| LOC643936 | purple |
| LOC644011 | purple |
| LOC644097 | purple |
| LOC644137 | purple |
| LOC644152 | purple |
| LOC644171 | purple |
| LOC644204 | purple |
| LOC644227 | purple |
| LOC644231 | purple |
| LOC644241 | purple |
| LOC644257 | purple |
| LOC644266 | purple |
| LOC644294 | purple |
| LOC644373 | purple |
| LOC644380 | purple |
| LOC644578 | purple |
| LOC644584 | purple |
| LOC644625 | purple |
| LOC644635 | purple |
| LOC644640 | purple |
| LOC644663 | purple |
| LOC644668 | purple |
| LOC644690 | purple |
| LOC644695 | purple |
| LOC644733 | purple |
| LOC644781 | purple |
| LOC644916 | purple |
| LOC644952 | purple |
| LOC644958 | purple |
| LOC644979 | purple |
| LOC644989 | purple |
| LOC645012 | purple |
| LOC645037 | purple |
| LOC645039 | purple |
| LOC645118 | purple |
| LOC645143 | purple |
| LOC645165 | purple |
| LOC645196 | purple |
| LOC645236 | purple |
| LOC645294 | purple |
| LOC645364 | purple |
| LOC645393 | purple |
| LOC645442 | purple |
| LOC645466 | purple |
| LOC645648 | purple |
| LOC645671 | purple |
| LOC645755 | purple |
| LOC645848 | purple |
| LOC645870 | purple |
| LOC645882 | purple |
| LOC645895 | purple |
| LOC645908 | purple |
| LOC645931 | purple |
| LOC645946 | purple |
| LOC645956 | purple |
| LOC646041 | purple |
| LOC646094 | purple |
| LOC646134 | purple |
| LOC646156 | purple |
| LOC646176 | purple |
| LOC646207 | purple |
| LOC646226 | purple |
| LOC646262 | purple |
| LOC646312 | purple |
| LOC646334 | purple |
| LOC646342 | purple |
| LOC646473 | purple |
| LOC646554 | purple |
| LOC646562 | purple |
| LOC646567 | purple |
| LOC646585 | purple |
| LOC646588 | purple |
| LOC646691 | purple |
| LOC646779 | purple |
| LOC646855 | purple |
| LOC646932 | purple |
| LOC647022 | purple |
| LOC647031 | purple |
| LOC647060 | purple |
| LOC647213 | purple |
| LOC647215 | purple |
| LOC647309 | purple |
| LOC647310 | purple |
| LOC647436 | purple |
| LOC647444 | purple |
| LOC647451 | purple |
| LOC647485 | purple |
| LOC647493 | purple |
| LOC647499 | purple |
| LOC647529 | purple |
| LOC647535 | purple |
| LOC647634 | purple |
| LOC647841 | purple |
| LOC647854 | purple |
| LOC647865 | purple |
| LOC647873 | purple |
| LOC647910 | purple |
| LOC647942 | purple |
| LOC648153 | purple |
| LOC648176 | purple |
| LOC648262 | purple |
| LOC648274 | purple |
| LOC648304 | purple |
| LOC648415 | purple |
| LOC648487 | purple |
| LOC648585 | purple |
| LOC648629 | purple |
| LOC648639 | purple |
| LOC648672 | purple |
| LOC648815 | purple |
| LOC648830 | purple |
| LOC648868 | purple |
| LOC648874 | purple |
| LOC648943 | purple |
| LOC649041 | purple |
| LOC649091 | purple |
| LOC649133 | purple |
| LOC649201 | purple |
| LOC649210 | purple |
| LOC649242 | purple |
| LOC649293 | purple |
| LOC649327 | purple |
| LOC649396 | purple |
| LOC649432 | purple |
| LOC649596 | purple |
| LOC649616 | purple |
| LOC649618 | purple |
| LOC649661 | purple |
| LOC649683 | purple |
| LOC649856 | purple |
| LOC649891 | purple |
| LOC649921 | purple |
| LOC649937 | purple |
| LOC649971 | purple |
| LOC650008 | purple |
| LOC650009 | purple |
| LOC650028 | purple |
| LOC650058 | purple |
| LOC650132 | purple |
| LOC650203 | purple |
| LOC650285 | purple |
| LOC650311 | purple |
| LOC650427 | purple |
| LOC650566 | purple |
| LOC650580 | purple |
| LOC650634 | purple |
| LOC650673 | purple |
| LOC650677 | purple |
| LOC650683 | purple |
| LOC650761 | purple |
| LOC650830 | purple |
| LOC650853 | purple |
| LOC650977 | purple |
| LOC651004 | purple |
| LOC651113 | purple |
| LOC651115 | purple |
| LOC651133 | purple |
| LOC651141 | purple |
| LOC651208 | purple |
| LOC651302 | purple |
| LOC651454 | purple |
| LOC651552 | purple |
| LOC651562 | purple |
| LOC651586 | purple |
| LOC651714 | purple |
| LOC651728 | purple |
| LOC651729 | purple |
| LOC651808 | purple |
| LOC651830 | purple |
| LOC651850 | purple |
| LOC651900 | purple |
| LOC651933 | purple |
| LOC652038 | purple |
| LOC652067 | purple |
| LOC652155 | purple |
| LOC652191 | purple |
| LOC652195 | purple |
| LOC652209 | purple |
| LOC652265 | purple |
| LOC652271 | purple |
| LOC652274 | purple |
| LOC652324 | purple |
| LOC652354 | purple |
| LOC652433 | purple |
| LOC652435 | purple |
| LOC652439 | purple |
| LOC652450 | purple |
| LOC652455 | purple |
| LOC652519 | purple |
| LOC652544 | purple |
| LOC652547 | purple |
| LOC652562 | purple |
| LOC652568 | purple |
| LOC652608 | purple |
| LOC652610 | purple |
| LOC652612 | purple |
| LOC652614 | purple |
| LOC652645 | purple |
| LOC652669 | purple |
| LOC652684 | purple |
| LOC652694 | purple |
| LOC652715 | purple |
| LOC652735 | purple |
| LOC652740 | purple |
| LOC652742 | purple |
| LOC652771 | purple |
| LOC652792 | purple |
| LOC652811 | purple |
| LOC652814 | purple |
| LOC652838 | purple |
| LOC652844 | purple |
| LOC652860 | purple |
| LOC652881 | purple |
| LOC652894 | purple |
| LOC653037 | purple |
| LOC653089 | purple |
| LOC653094 | purple |
| LOC653100 | purple |
| LOC653127 | purple |
| LOC653136 | purple |
| LOC653206 | purple |
| LOC653214 | purple |
| LOC653316 | purple |
| LOC653520 | purple |
| LOC653555 | purple |
| LOC653564 | purple |
| LOC653582 | purple |
| LOC653600 | purple |
| LOC653610 | purple |
| LOC653686 | purple |
| LOC653765 | purple |
| LOC653787 | purple |
| LOC653889 | purple |
| LOC653904 | purple |
| LOC653934 | purple |
| LOC653937 | purple |
| LOC653941 | purple |
| LOC653968 | purple |
| LOC654002 | purple |
| LOC654056 | purple |
| LOC654074 | purple |
| LOC654080 | purple |
| LOC654133 | purple |
| LOC654165 | purple |
| LOC654206 | purple |
| LOC723972 | purple |
| LOC728006 | purple |
| LOC728014 | purple |
| LOC728089 | purple |
| LOC728302 | purple |
| LOC728411 | purple |
| LOC728516 | purple |
| LOC728758 | purple |
| LOC729025 | purple |
| LOC729370 | purple |
| LOC729396 | purple |
| LOC729776 | purple |
| LOC729837 | purple |
| LOC729900 | purple |
| LOC730031 | purple |
| LOC730051 | purple |
| LOC730109 | purple |
| LOC730274 | purple |
| LOC730340 | purple |
| LOC730347 | purple |
| LOC730479 | purple |
| LOC730525 | purple |
| LOC730841 | purple |
| LOC730953 | purple |
| LOC731017 | purple |
| LOC731035 | purple |
| LOC731052 | purple |
| LOC731431 | purple |
| LOC731438 | purple |
| LOC731444 | purple |
| LOC731823 | purple |
| LOC732040 | purple |
| LOC732138 | purple |
| LOC732300 | purple |
| LOC732424 | purple |
| LOC81691 | purple |
| LOC93622 | purple |
| LOH12CR1 | purple |
| LOXHD1 | purple |
| LPAR5 | purple |
| LRGUK | purple |
| LRIG2 | purple |
| LRMP | purple |
| LRP2 | purple |
| LRRC24 | purple |
| LRRC37A2 | purple |
| LRRC37A3 | purple |
| LSM3 | purple |
| LSM7 | purple |
| LTA4H | purple |
| LTB4R | purple |
| MAGEF1 | purple |
| MAGOHB | purple |
| MAN2A2 | purple |
| MAP3K2 | purple |
| MAP9 | purple |
| MAPK4 | purple |
| MAPKBP1 | purple |
| MARS2 | purple |
| MAST1 | purple |
| MAST3 | purple |
| MAT2A | purple |
| MBTD1 | purple |
| MCL1 | purple |
| MCM2 | purple |
| MCM5 | purple |
| MCMDC1 | purple |
| MDN1 | purple |
| MECP2 | purple |
| MEGF10 | purple |
| MESP1 | purple |
| METTL14 | purple |
| MFI2 | purple |
| MFSD7 | purple |
| MFSD8 | purple |
| MFSD9 | purple |
| MGAM | purple |
| MGC13168 | purple |
| MGC18216 | purple |
| MGC33407 | purple |
| MICAL3 | purple |
| MID2 | purple |
| MIER1 | purple |
| MKRN1 | purple |
| MLKL | purple |
| MMP15 | purple |
| MMP23A | purple |
| MORC3 | purple |
| MOSPD1 | purple |
| MPP7 | purple |
| MPPE1 | purple |
| MPZL3 | purple |
| MR1 | purple |
| MRPL11 | purple |
| MRPL15 | purple |
| MRPL33 | purple |
| MSGN1 | purple |
| MSTN | purple |
| MSTO1 | purple |
| MT3 | purple |
| MTCH2 | purple |
| MTE | purple |
| MTERFD3 | purple |
| MTHFS | purple |
| MTM | purple |
| MTMR6 | purple |
| MTRF1 | purple |
| MTRR | purple |
| MTX3 | purple |
| MVP | purple |
| MXRA7 | purple |
| MYCBP | purple |
| MYH16 | purple |
| MYL4 | purple |
| MYO9A | purple |
| MYOZ1 | purple |
| MYOZ2 | purple |
| MYST4 | purple |
| NAB1 | purple |
| NACC2 | purple |
| NANOG | purple |
| NANOS2 | purple |
| NANP | purple |
| NAT15 | purple |
| NAV3 | purple |
| NCAPH2 | purple |
| NCF4 | purple |
| NCK2 | purple |
| NCOA5 | purple |
| NCOR1 | purple |
| NCR2 | purple |
| NDST1 | purple |
| NDUFA4L2 | purple |
| NDUFS3 | purple |
| NDUFS6 | purple |
| NEK9 | purple |
| NEURL | purple |
| NEUROG3 | purple |
| NFIX | purple |
| NFKBIA | purple |
| NFYB | purple |
| NHP2 | purple |
| NKD1 | purple |
| NKX2-6 | purple |
| NLE1 | purple |
| NLGN2 | purple |
| NLRP2 | purple |
| NLRP8 | purple |
| NOL4 | purple |
| NONO | purple |
| NOTCH2 | purple |
| NOVA2 | purple |
| NP | purple |
| NPAL3 | purple |
| NPAS2 | purple |
| NPB | purple |
| NPC2 | purple |
| NR1I3 | purple |
| NRG3 | purple |
| NT5C3L | purple |
| NUBP2 | purple |
| NUDT13 | purple |
| NUP35 | purple |
| NXPH1 | purple |
| OMP | purple |
| OPTC | purple |
| OR10A4 | purple |
| OR10G8 | purple |
| OR10J5 | purple |
| OR13F1 | purple |
| OR2A25 | purple |
| OR2A7 | purple |
| OR2A9P | purple |
| OR2G2 | purple |
| OR2G6 | purple |
| OR2Z1 | purple |
| OR4C46 | purple |
| OR4F16 | purple |
| OR4F4 | purple |
| OR4L1 | purple |
| OR51E2 | purple |
| OR51V1 | purple |
| OR52E5 | purple |
| OR52H1 | purple |
| OR56B4 | purple |
| OR5AK2 | purple |
| OR5K1 | purple |
| OR5K2 | purple |
| OR6C4 | purple |
| OR6K2 | purple |
| OR7E24 | purple |
| OR8B3 | purple |
| OR8K3 | purple |
| OR9A4 | purple |
| ORC2L | purple |
| OSTF1 | purple |
| OTOF | purple |
| OXTR | purple |
| P2RXL1 | purple |
| P2RY8 | purple |
| PA2G4 | purple |
| PAAF1 | purple |
| PACSIN3 | purple |
| PAG1 | purple |
| PARK7 | purple |
| PATL1 | purple |
| PCDHA4 | purple |
| PCYT2 | purple |
| PDCD1 | purple |
| PDE10A | purple |
| PDE7B | purple |
| PDIA2 | purple |
| PDS5B | purple |
| PDZK1IP1 | purple |
| PDZRN3 | purple |
| PER3 | purple |
| PEX6 | purple |
| PFAAP5 | purple |
| PFDN1 | purple |
| PFTK1 | purple |
| PGF | purple |
| PGRMC2 | purple |
| PHF10 | purple |
| PHF2 | purple |
| PHF21A | purple |
| PHF5A | purple |
| PHF8 | purple |
| PHKA2 | purple |
| PI4K2A | purple |
| PICK1 | purple |
| PIGK | purple |
| PIK3R3 | purple |
| PIM2 | purple |
| PIP | purple |
| PIP4K2C | purple |
| PKN3 | purple |
| PLA2G3 | purple |
| PLAT | purple |
| PLCG1 | purple |
| PLDN | purple |
| PLEKHA2 | purple |
| PLEKHA8 | purple |
| PLEKHG4 | purple |
| PLK4 | purple |
| PLS1 | purple |
| PLTP | purple |
| PLXDC1 | purple |
| PLXNA3 | purple |
| PLXNB2 | purple |
| PM20D1 | purple |
| PNLIPRP2 | purple |
| PNPLA3 | purple |
| PNPLA4 | purple |
| PODXL | purple |
| POLDIP2 | purple |
| POLR2F | purple |
| POMC | purple |
| POP1 | purple |
| POPDC3 | purple |
| POU5F2 | purple |
| PPAP2A | purple |
| PPFIBP2 | purple |
| PPP1R10 | purple |
| PPP1R12C | purple |
| PPP1R14C | purple |
| PQLC1 | purple |
| PRAMEF8 | purple |
| PRED57 | purple |
| PRG3 | purple |
| PRKAA1 | purple |
| PRKAB1 | purple |
| PRKAR2A | purple |
| PRKAR2B | purple |
| PRKCDBP | purple |
| PRKD3 | purple |
| PRL | purple |
| PRMT7 | purple |
| PRO0628 | purple |
| PRO1853 | purple |
| PROK2 | purple |
| PRPF3 | purple |
| PRSS2 | purple |
| PRSS38 | purple |
| PSMB7 | purple |
| PSMD14 | purple |
| PTAFR | purple |
| PTCHD3 | purple |
| PTGFRN | purple |
| PTGS2 | purple |
| PTH2 | purple |
| PTPN9 | purple |
| PTPRV | purple |
| PTTG2 | purple |
| PUS7 | purple |
| QARS | purple |
| RAB12 | purple |
| RAB18 | purple |
| RAB2A | purple |
| RAB2B | purple |
| RAB34 | purple |
| RAB39B | purple |
| RAB3IL1 | purple |
| RABL4 | purple |
| RANBP1 | purple |
| RANBP3L | purple |
| RAPGEFL1 | purple |
| RASSF4 | purple |
| RBBP8 | purple |
| RBED1 | purple |
| RBKS | purple |
| RBM23 | purple |
| RBMX2 | purple |
| RCN1 | purple |
| REEP4 | purple |
| REG1B | purple |
| RELA | purple |
| REST | purple |
| RETNLB | purple |
| REXO2 | purple |
| REXO4 | purple |
| RFX3 | purple |
| RGS16 | purple |
| RGS22 | purple |
| RGSL1 | purple |
| RIBC1 | purple |
| RIG | purple |
| RIN3 | purple |
| RIPK1 | purple |
| RMND5A | purple |
| RNASE10 | purple |
| RNF113B | purple |
| RNF141 | purple |
| RNF181 | purple |
| RNF190 | purple |
| RNMT | purple |
| RNPC3 | purple |
| ROM1 | purple |
| RP1 | purple |
| RP11-49G10.8 | purple |
| RP2 | purple |
| RPA2 | purple |
| RPE65 | purple |
| RPH3AL | purple |
| RPLP1 | purple |
| RPS6 | purple |
| RPS6KA6 | purple |
| RRP1B | purple |
| RSL1D1 | purple |
| RXRA | purple |
| SACM1L | purple |
| SAE1 | purple |
| SAMD4A | purple |
| SARM1 | purple |
| SASH3 | purple |
| SAT2 | purple |
| SBNO2 | purple |
| SCGB2A2 | purple |
| SCML4 | purple |
| SCN9A | purple |
| SCO2 | purple |
| SCUBE2 | purple |
| SDCCAG1 | purple |
| SEC24D | purple |
| SEC61B | purple |
| SENP2 | purple |
| SERPINA9 | purple |
| SERPINB4 | purple |
| SETD3 | purple |
| SF3B14 | purple |
| SFRS13B | purple |
| SFT2D2 | purple |
| SGK269 | purple |
| SH3BP4 | purple |
| SH3TC2 | purple |
| SIGLEC15 | purple |
| SIN3B | purple |
| SIRPB2 | purple |
| SIRT4 | purple |
| SIT1 | purple |
| SKA1 | purple |
| SLC10A5 | purple |
| SLC18A3 | purple |
| SLC24A2 | purple |
| SLC25A34 | purple |
| SLC25A44 | purple |
| SLC27A4 | purple |
| SLC29A3 | purple |
| SLC30A1 | purple |
| SLC30A2 | purple |
| SLC30A8 | purple |
| SLC36A3 | purple |
| SLC38A9 | purple |
| SLC46A3 | purple |
| SLC47A1 | purple |
| SLC4A4 | purple |
| SLC4A9 | purple |
| SLC5A6 | purple |
| SLC5A8 | purple |
| SLC7A6OS | purple |
| SLC8A1 | purple |
| SLC8A2 | purple |
| SLC9A5 | purple |
| SLCO4C1 | purple |
| SLCO6A1 | purple |
| SLFN12L | purple |
| SMARCB1 | purple |
| SMCR7 | purple |
| SMYD4 | purple |
| SNAPC1 | purple |
| SNCG | purple |
| SNORD42A | purple |
| SNRNP35 | purple |
| SNRPA1 | purple |
| SNUPN | purple |
| SNX2 | purple |
| SOBP | purple |
| SOCS4 | purple |
| SOS2 | purple |
| SPACA5B | purple |
| SPANXB1 | purple |
| SPATS1 | purple |
| SPATS2L | purple |
| SPEF1 | purple |
| SPEN | purple |
| SPG3A | purple |
| SPIN1 | purple |
| SPINK1 | purple |
| SPRR2C | purple |
| SRD5A2 | purple |
| SRF | purple |
| SRM | purple |
| SRP14P1 | purple |
| SSH1 | purple |
| SSH2 | purple |
| SSTR1 | purple |
| ST6GAL2 | purple |
| STAG3L4 | purple |
| STELLAR | purple |
| STK31 | purple |
| STRC | purple |
| SUCLG2 | purple |
| SV2A | purple |
| SVOPL | purple |
| SYNGR2 | purple |
| SYTL3 | purple |
| SYTL5 | purple |
| TAGLN3 | purple |
| TARDBP | purple |
| TAS2R7 | purple |
| TBC1D23 | purple |
| TBC1D3 | purple |
| TBC1D4 | purple |
| TBXA2R | purple |
| TCAP | purple |
| TCEAL7 | purple |
| TCF3 | purple |
| TDRD9 | purple |
| TECPR2 | purple |
| TEKT4 | purple |
| TESK1 | purple |
| TFAP4 | purple |
| TFCP2 | purple |
| TGIF2 | purple |
| TGM2 | purple |
| THAP3 | purple |
| THAP4 | purple |
| THPO | purple |
| TIGD3 | purple |
| TIMM22 | purple |
| TIMM50 | purple |
| TINF2 | purple |
| TLN1 | purple |
| TLN2 | purple |
| TMED2 | purple |
| TMEM108 | purple |
| TMEM125 | purple |
| TMEM135 | purple |
| TMEM149 | purple |
| TMEM14A | purple |
| TMEM154 | purple |
| TMEM16C | purple |
| TMEM184A | purple |
| TMEM195 | purple |
| TMEM2 | purple |
| TMEM5 | purple |
| TMEM76 | purple |
| TMPRSS11A | purple |
| TMPRSS11B | purple |
| TMPRSS2 | purple |
| TNFSF8 | purple |
| TNIP2 | purple |
| TNMD | purple |
| TNNT3 | purple |
| TOB2 | purple |
| TPD52L3 | purple |
| TPP1 | purple |
| TPPP2 | purple |
| TPRG1L | purple |
| TRIM31 | purple |
| TRIM58 | purple |
| TRO | purple |
| TROVE2 | purple |
| TRPC1 | purple |
| TRPC3 | purple |
| TRPC4AP | purple |
| TRPM2 | purple |
| TRY1 | purple |
| TSFM | purple |
| TSSC4 | purple |
| TSSK2 | purple |
| TTC19 | purple |
| TTLL8 | purple |
| TTTY12 | purple |
| TTYH2 | purple |
| TUBB4 | purple |
| TUBB8 | purple |
| TUBGCP3 | purple |
| TUG1 | purple |
| TXK | purple |
| TXNL4B | purple |
| TYRO3P | purple |
| TYROBP | purple |
| UBASH3A | purple |
| UBE2T | purple |
| UBE4A | purple |
| UBL5 | purple |
| UBTD1 | purple |
| UBXD7 | purple |
| UCK1 | purple |
| UFC1 | purple |
| UGT1A9 | purple |
| UGT3A2 | purple |
| UNC5D | purple |
| UNK | purple |
| UPB1 | purple |
| UPK3A | purple |
| UPP2 | purple |
| USP19 | purple |
| USP50 | purple |
| VDR | purple |
| VGF | purple |
| WASPIP | purple |
| WDR6 | purple |
| WDR75 | purple |
| WDR82 | purple |
| WFIKKN2 | purple |
| WHAMML1 | purple |
| WIPF1 | purple |
| WIPF2 | purple |
| WISP2 | purple |
| WNK1 | purple |
| WSB1 | purple |
| WWOX | purple |
| WWTR1 | purple |
| XAGE-4 | purple |
| XCL1 | purple |
| XPNPEP2 | purple |
| XPO5 | purple |
| XPO7 | purple |
| XRCC4 | purple |
| XRN1 | purple |
| XYLT2 | purple |
| YPEL3 | purple |
| YWHAH | purple |
| ZACN | purple |
| ZBED3 | purple |
| ZBP1 | purple |
| ZBTB10 | purple |
| ZBTB7A | purple |
| ZC3H6 | purple |
| ZC3H7A | purple |
| ZCCHC14 | purple |
| ZCCHC4 | purple |
| ZCCHC7 | purple |
| ZCCHC9 | purple |
| ZDHHC17 | purple |
| ZFP14 | purple |
| ZFP30 | purple |
| ZFYVE28 | purple |
| ZMYM5 | purple |
| ZMYND10 | purple |
| ZNF134 | purple |
| ZNF138 | purple |
| ZNF146 | purple |
| ZNF167 | purple |
| ZNF189 | purple |
| ZNF195 | purple |
| ZNF212 | purple |
| ZNF215 | purple |
| ZNF236 | purple |
| ZNF257 | purple |
| ZNF286A | purple |
| ZNF365 | purple |
| ZNF367 | purple |
| ZNF385D | purple |
| ZNF462 | purple |
| ZNF503 | purple |
| ZNF507 | purple |
| ZNF509 | purple |
| ZNF581 | purple |
| ZNF585B | purple |
| ZNF628 | purple |
| ZNF642 | purple |
| ZNF650 | purple |
| ZNF658 | purple |
| ZNF761 | purple |
| ZNF766 | purple |
| ZNF791 | purple |
| ZNF804B | purple |
| ZNF827 | purple |
| ZNF831 | purple |
| ZNF85 | purple |
| ZNHIT2 | purple |
| ZSCAN18 | purple |
| ZUFSP | purple |
| A2BP1 | salmon |
| ACN9 | salmon |
| ADH5 | salmon |
| AMDHD2 | salmon |
| AMOTL1 | salmon |
| ANAPC5 | salmon |
| ANKDD1A | salmon |
| ARID3B | salmon |
| C16orf33 | salmon |
| C1orf125 | salmon |
| C1orf144 | salmon |
| C1orf192 | salmon |
| C1orf56 | salmon |
| C8orf22 | salmon |
| CCL3 | salmon |
| CD151 | salmon |
| CD28 | salmon |
| CDH22 | salmon |
| CYCSL1 | salmon |
| DHCR7 | salmon |
| DPPA3 | salmon |
| DTNB | salmon |
| DUX3 | salmon |
| E2F2 | salmon |
| EPC2 | salmon |
| FAM44B | salmon |
| FBL | salmon |
| FICD | salmon |
| FLJ11795 | salmon |
| FRAS1 | salmon |
| GLT8D1 | salmon |
| GNL2 | salmon |
| GPR160 | salmon |
| GPR27 | salmon |
| HCK | salmon |
| HDHD1A | salmon |
| HSDL1 | salmon |
| IFT122 | salmon |
| IL22RA1 | salmon |
| KLHL14 | salmon |
| KRTAP9-8 | salmon |
| LOC197135 | salmon |
| LOC388849 | salmon |
| LOC402643 | salmon |
| LOC441870 | salmon |
| LOC442329 | salmon |
| LOC642354 | salmon |
| LOC642509 | salmon |
| LOC642736 | salmon |
| LOC643435 | salmon |
| LOC644048 | salmon |
| LOC645335 | salmon |
| LOC645737 | salmon |
| LOC646383 | salmon |
| LOC646627 | salmon |
| LOC646804 | salmon |
| LOC649431 | salmon |
| LOC651029 | salmon |
| LOC651787 | salmon |
| LOC651986 | salmon |
| LOC653043 | salmon |
| LOC653321 | salmon |
| LOC729485 | salmon |
| LOC90586 | salmon |
| LYPD2 | salmon |
| LYRM1 | salmon |
| MAGEB18 | salmon |
| MRPL51 | salmon |
| NEIL3 | salmon |
| NSMCE1 | salmon |
| NUDT2 | salmon |
| OR1L6 | salmon |
| OR4C45 | salmon |
| OR4D2 | salmon |
| OR56A1 | salmon |
| PBX1 | salmon |
| PGLYRP4 | salmon |
| PLCD3 | salmon |
| PLEKHH2 | salmon |
| PLXNC1 | salmon |
| QSOX1 | salmon |
| RBM25 | salmon |
| RCE1 | salmon |
| SDS | salmon |
| SGCB | salmon |
| SH3GLB1 | salmon |
| SH3PXD2B | salmon |
| SHANK1 | salmon |
| SIRT5 | salmon |
| SNAP91 | salmon |
| ST6GALNAC2 | salmon |
| ST7 | salmon |
| SYCP2L | salmon |
| TIMM17A | salmon |
| TOMM34 | salmon |
| TXNDC6 | salmon |
| UBE2Q2 | salmon |
| UBE3B | salmon |
| UPF3A | salmon |
