# Supplementary material for: Single-Cell Sequencing Analysis and Multiple Machine Learning Methods Identified G0S2 and HPSE as Novel Biomarkers for Abdominal Aortic Aneurysm
Source: Front Immunol. 2022 Jun 13;13:907309. doi: 10.3389/fimmu.2022.907309 (PMC9234288; doi:10.3389/fimmu.2022.907309)
Supplement: Supplementary Table 4 — DEGs between AAA and normal samples. [file Table_4.doc]

**Supplementary table 4**

| Genes | logFC | AveExpr | t | P.Value | adj.P.Val | B |
| --- | --- | --- | --- | --- | --- | --- |
| MT1M | -2.59822971 | 0.016031686 | -10.15719374 | 9.97E-15 | 4.26E-11 | 22.97487787 |
| CXXC5 | -1.963365098 | 0.011559468 | -9.565178659 | 9.65E-14 | 1.70E-10 | 20.82805106 |
| WDR55 | -0.885153754 | 0.005838403 | -9.509918483 | 1.19E-13 | 1.70E-10 | 20.62571914 |
| MFHAS1 | -1.319460619 | 0.022729241 | -9.154420446 | 4.75E-13 | 5.08E-10 | 19.31687976 |
| UGCGL2 | -1.072241876 | 0.068773097 | -8.625562455 | 3.77E-12 | 3.22E-09 | 17.34931332 |
| PCNT | -0.875730408 | 0.03158423 | -7.716897608 | 1.36E-10 | 9.72E-08 | 13.92874025 |
| ASAM | -1.133487622 | 0.062950475 | -7.471970393 | 3.60E-10 | 2.20E-07 | 13.0024183 |
| SYNC1 | -1.44557181 | 0.102930366 | -7.391111705 | 4.96E-10 | 2.65E-07 | 12.69655749 |
| PRPF19 | -0.721852031 | 0.013023372 | -7.329616455 | 6.33E-10 | 3.01E-07 | 12.46396645 |
| C16orf68 | -0.73952146 | 0.067402802 | -6.906833373 | 3.37E-09 | 1.44E-06 | 10.86698481 |
| TXNDC15 | -0.796550863 | 0.014027467 | -6.834455451 | 4.49E-09 | 1.75E-06 | 10.59423535 |
| FXR2 | -0.797225984 | 0.009834564 | -6.635272007 | 9.84E-09 | 3.27E-06 | 9.845161508 |
| DNM1L | -0.684323632 | 0.024355344 | -6.632935603 | 9.93E-09 | 3.27E-06 | 9.836390291 |
| NAT6 | -0.590271855 | 0.005429874 | -6.458897947 | 1.97E-08 | 6.01E-06 | 9.184215874 |
| C3orf39 | -0.75517402 | 0.033534919 | -6.407134178 | 2.41E-08 | 6.33E-06 | 8.990738604 |
| SLC20A2 | -0.932247196 | 0.027790754 | -6.389371714 | 2.58E-08 | 6.33E-06 | 8.924405713 |
| DOLK | -0.608551876 | 0.026865127 | -6.388164447 | 2.59E-08 | 6.33E-06 | 8.919898343 |
| AXIN2 | -1.122222825 | 0.013405533 | -6.37397736 | 2.74E-08 | 6.33E-06 | 8.866941045 |
| TRK1 | -1.265618312 | 0.024168272 | -6.358332753 | 2.91E-08 | 6.33E-06 | 8.808566105 |
| GALNTL2 | -1.300926009 | 0.049070622 | -6.354424598 | 2.96E-08 | 6.33E-06 | 8.793987356 |
| RBPMS2 | -1.628519449 | 0.008327488 | -6.283773536 | 3.90E-08 | 7.94E-06 | 8.530705697 |
| TIMP4 | -1.772961046 | 0.090186889 | -6.197733523 | 5.45E-08 | 1.06E-05 | 8.210809636 |
| ZBTB17 | -0.66896858 | 0.027890767 | -6.171657424 | 6.04E-08 | 1.12E-05 | 8.114027662 |
| NPTX2 | -1.513620164 | 0.072057004 | -6.119970217 | 7.38E-08 | 1.31E-05 | 7.922433912 |
| LOC93622 | -0.556403676 | 0.045088797 | -6.034626935 | 1.03E-07 | 1.76E-05 | 7.606831651 |
| AKAP8L | -0.770590386 | 0.073091631 | -5.995328308 | 1.20E-07 | 1.97E-05 | 7.461833953 |
| MRAS | 0.735805426 | 0.027007329 | 5.902392028 | 1.71E-07 | 2.71E-05 | 7.119813502 |
| C9orf123 | -0.843057159 | 0.014424604 | -5.868017452 | 1.95E-07 | 2.98E-05 | 6.993637896 |
| AKAP8 | -0.657721266 | 0.005557609 | -5.807229163 | 2.47E-07 | 3.64E-05 | 6.770964268 |
| C7orf55 | -0.609330257 | 0.002891695 | -5.76070391 | 2.95E-07 | 4.19E-05 | 6.600946223 |
| UPF3B | -0.577761797 | 0.00324477 | -5.753478439 | 3.03E-07 | 4.19E-05 | 6.574574784 |
| GPRC5C | -0.689891844 | 0.005755519 | -5.699087457 | 3.74E-07 | 4.84E-05 | 6.376349326 |
| FCN1 | 1.468365765 | 0.144205261 | 5.446286281 | 9.76E-07 | 0.000119283 | 5.462282782 |
| CALCOCO2 | -0.663859326 | 0.005864523 | -5.388123645 | 1.22E-06 | 0.000144399 | 5.253818812 |
| SLC24A3 | -1.201518122 | 0.081104109 | -5.369402503 | 1.30E-06 | 0.000149474 | 5.186876448 |
| C3orf70 | -1.162222773 | 0.071579087 | -5.364564056 | 1.33E-06 | 0.000149474 | 5.169587972 |
| PCNXL3 | -0.61716481 | 0.052898469 | -5.317573219 | 1.58E-06 | 0.000173737 | 5.001957461 |
| KIAA1539 | -0.765008002 | 0.027367019 | -5.298589915 | 1.70E-06 | 0.00018188 | 4.934381589 |
| ATP1A2 | -1.354444157 | 0.141489106 | -5.265901209 | 1.92E-06 | 0.000197491 | 4.818215136 |
| GSDMB | 0.749192353 | 0.231396095 | 5.153039767 | 2.93E-06 | 0.000282612 | 4.419121104 |
| TASP1 | -0.519352713 | 0.020048661 | -5.127890679 | 3.21E-06 | 0.000298716 | 4.330623995 |
| PEG3 | -0.860848401 | 0.005208717 | -5.109550513 | 3.44E-06 | 0.000312906 | 4.266189526 |
| ANAPC4 | -0.798488846 | 0.040315244 | -5.085918284 | 3.75E-06 | 0.000320993 | 4.183291878 |
| LMBR1 | -0.585330269 | 0.002714918 | -5.044169534 | 4.38E-06 | 0.000346717 | 4.03720658 |
| KLHL9 | -1.132558896 | 0.012792966 | -5.02687605 | 4.67E-06 | 0.000360627 | 3.976831518 |
| VPS26 | -0.707567332 | 0.006449027 | -5.023610044 | 4.72E-06 | 0.000360627 | 3.965438374 |
| PNKP | -0.506159497 | 0.025772915 | -5.00340329 | 5.09E-06 | 0.000381627 | 3.895014213 |
| PFKP | -0.957728751 | 0.066970454 | -4.963552856 | 5.89E-06 | 0.000427555 | 3.756460738 |
| UCKL1 | -0.55953352 | 0.037316399 | -4.962331665 | 5.91E-06 | 0.000427555 | 3.752221904 |
| LOC648984 | 0.571885997 | 0.033095925 | 4.958443083 | 6.00E-06 | 0.000427555 | 3.738727188 |
| MRPL20 | -1.242943791 | 0.057076321 | -4.930313909 | 6.65E-06 | 0.000458616 | 3.641237617 |
| ADAMTS8 | -1.197410688 | 0.027915838 | -4.91136413 | 7.13E-06 | 0.000483682 | 3.575690131 |
| MRPL41 | -0.574255429 | 0.00743262 | -4.90393316 | 7.32E-06 | 0.000489211 | 3.550014801 |
| VKORC1L1 | -0.502439471 | 0.004915453 | -4.865340634 | 8.43E-06 | 0.00054599 | 3.416931698 |
| HRCT1 | -1.238034728 | 0.134479038 | -4.841521124 | 9.19E-06 | 0.000577847 | 3.335013274 |
| LOC641849 | -1.085575578 | 0.020569065 | -4.805243299 | 1.05E-05 | 0.000649523 | 3.210579101 |
| LOC728014 | -0.71924888 | 0.031272626 | -4.774082381 | 1.17E-05 | 0.000706512 | 3.104019301 |
| LOC441763 | -2.261706987 | 0.032668047 | -4.75252241 | 1.27E-05 | 0.000742746 | 3.030469016 |
| LBA1 | 0.546828734 | 0.109749578 | 4.74120272 | 1.32E-05 | 0.000763215 | 2.991911482 |
| C9orf110 | 0.665855385 | 0.050986853 | 4.704189504 | 1.51E-05 | 0.000851833 | 2.866121585 |
| ARRDC5 | 0.502761291 | 0.045970064 | 4.691049852 | 1.58E-05 | 0.000878367 | 2.821572703 |
| GRSF1 | -0.788143534 | 0.025905673 | -4.68668531 | 1.61E-05 | 0.000880785 | 2.806787537 |
| LOC347544 | -1.704339226 | 0.013572516 | -4.6819796 | 1.63E-05 | 0.00088443 | 2.790853625 |
| LOC197135 | 0.612827059 | 0.070016346 | 4.668900117 | 1.71E-05 | 0.000907244 | 2.746603629 |
| BMI1 | -0.936346223 | 0.010212896 | -4.664617909 | 1.74E-05 | 0.000907244 | 2.732128478 |
| C3orf58 | -0.63781112 | 0.001376314 | -4.659599377 | 1.77E-05 | 0.000912042 | 2.715172049 |
| TRIB3 | -0.736223772 | 0.031507411 | -4.653874833 | 1.81E-05 | 0.000919824 | 2.695840368 |
| GTF2H4 | -0.61818591 | 0.037658351 | -4.644344124 | 1.87E-05 | 0.000940499 | 2.663679521 |
| WDR60 | -0.629890908 | 0.04855071 | -4.635024524 | 1.93E-05 | 0.000961021 | 2.632260386 |
| TMEM216 | -0.695317618 | 0.013648847 | -4.621726463 | 2.03E-05 | 0.00098853 | 2.587479175 |
| AIF1L | -0.803413917 | 0.019900895 | -4.620668772 | 2.03E-05 | 0.00098853 | 2.58391996 |
| CNR1 | -0.6246301 | 0.057111278 | -4.607973662 | 2.13E-05 | 0.001022652 | 2.541229458 |
| LOC149448 | -0.571446968 | 0.024133322 | -4.600278231 | 2.19E-05 | 0.001027955 | 2.515378293 |
| LOC729021 | 1.264832225 | 0.53918159 | 4.593429025 | 2.24E-05 | 0.001041862 | 2.492386839 |
| CSRNP2 | -0.513430056 | 0.017104895 | -4.570764772 | 2.43E-05 | 0.001105203 | 2.416422166 |
| ZC3H12A | 1.038463204 | 0.33866465 | 4.565275897 | 2.48E-05 | 0.001108767 | 2.398051566 |
| SEMA4A | 0.89181159 | 0.284435165 | 4.540607456 | 2.70E-05 | 0.001182864 | 2.315618797 |
| LOC641825 | 0.756227614 | 0.199895307 | 4.514500289 | 2.97E-05 | 0.001228451 | 2.228611229 |
| NIT2 | -0.562565739 | 0.019112308 | -4.514275939 | 2.97E-05 | 0.001228451 | 2.227864582 |
| BIN2 | 0.520228823 | 0.06361715 | 4.512551035 | 2.99E-05 | 0.001228451 | 2.222124624 |
| EPHB4 | -0.858845137 | 0.002173087 | -4.512374988 | 2.99E-05 | 0.001228451 | 2.221538851 |
| LOC643997 | -0.852361781 | 0.139013594 | -4.511280057 | 3.00E-05 | 0.001228451 | 2.21789587 |
| CDO1 | -0.779117422 | 0.029616867 | -4.504158921 | 3.08E-05 | 0.001228451 | 2.194213331 |
| PRO0628 | 0.581338445 | 0.066412978 | 4.482565856 | 3.32E-05 | 0.001301819 | 2.122513015 |
| C10orf32 | -0.686708091 | 0.027397653 | -4.462133672 | 3.57E-05 | 0.001361201 | 2.054822426 |
| C9orf95 | -0.713201815 | 0.000315494 | -4.458055442 | 3.62E-05 | 0.001361201 | 2.04132967 |
| DYNLL2 | -0.505031187 | 0.014902978 | -4.457125599 | 3.63E-05 | 0.001361201 | 2.038254149 |
| LOC85390 | 0.614462919 | 0.063806015 | 4.412976058 | 4.24E-05 | 0.001575159 | 1.892592554 |
| ADCY2 | 0.578771581 | 0.074567635 | 4.397267074 | 4.48E-05 | 0.001649694 | 1.840938833 |
| FIBIN | -0.799926472 | 0.08352949 | -4.366652737 | 4.98E-05 | 0.001791741 | 1.74054083 |
| EXOSC6 | -0.594785263 | 0.014961942 | -4.366249802 | 4.99E-05 | 0.001791741 | 1.7392218 |
| LEPREL1 | -0.747874517 | 0.019280972 | -4.358984346 | 5.11E-05 | 0.001798387 | 1.715448492 |
| SPAG5 | 0.51641712 | 0.03699485 | 4.354953667 | 5.19E-05 | 0.001798387 | 1.7022684 |
| KHNYN | 0.556978908 | 0.179912113 | 4.336290035 | 5.53E-05 | 0.001863111 | 1.641320662 |
| MIAT | 0.873050739 | 0.216404923 | 4.313847872 | 5.98E-05 | 0.001952763 | 1.568212019 |
| CBS | -0.794864296 | 0.023470026 | -4.313784431 | 5.98E-05 | 0.001952763 | 1.568005629 |
| ZNF791 | -0.613999169 | 0.020371912 | -4.298189207 | 6.31E-05 | 0.002014878 | 1.517317864 |
| ZW10 | -0.578787846 | 0.005027221 | -4.298187118 | 6.31E-05 | 0.002014878 | 1.517311082 |
| LOC654121 | -0.7361771 | 0.065589127 | -4.283235224 | 6.65E-05 | 0.00209435 | 1.468803981 |
| ZCCHC6 | -0.815121411 | 0.129704716 | -4.282696474 | 6.66E-05 | 0.00209435 | 1.467057807 |
| SLC36A4 | -0.603403952 | 0.011094367 | -4.231280111 | 7.95E-05 | 0.002411106 | 1.300941654 |
| LOC401357 | 0.686689046 | 0.06819606 | 4.154647058 | 0.000103309 | 0.002991581 | 1.05534746 |
| PPP3R1 | -1.065818558 | 0.038342383 | -4.153980017 | 0.000103544 | 0.002991581 | 1.053220386 |
| UBL3 | -0.74354302 | 0.013303716 | -4.145364573 | 0.000106623 | 0.003027776 | 1.025764015 |
| LOC644330 | 0.560479582 | 0.165761205 | 4.144543247 | 0.000106921 | 0.003027776 | 1.023148175 |
| PHF21A | -0.557231663 | 0.024152645 | -4.13426967 | 0.000110719 | 0.003088849 | 0.990451825 |
| CEP350 | -0.705979973 | 0.000147601 | -4.131434836 | 0.000111789 | 0.003088849 | 0.9814376 |
| LOC643423 | 0.623881652 | 0.067715706 | 4.130966059 | 0.000111967 | 0.003088849 | 0.979947307 |
| EBPL | -0.523715509 | 0.02186511 | -4.121005235 | 0.000115815 | 0.003174513 | 0.94830273 |
| LEPRE1 | -0.590785317 | 0.013474653 | -4.118459922 | 0.000116818 | 0.003181624 | 0.94022326 |
| PLCH2 | 0.567708079 | 0.116446411 | 4.106258577 | 0.000121747 | 0.003294863 | 0.901531334 |
| GPR4 | -0.568665392 | 0.082492992 | -4.09815675 | 0.000125129 | 0.00334408 | 0.875874548 |
| HNRPH1 | 0.603151257 | 0.094658462 | 4.082812811 | 0.000131786 | 0.003480628 | 0.827360615 |
| UCP2 | 1.173937372 | 0.329844273 | 4.082631905 | 0.000131867 | 0.003480628 | 0.826789235 |
| SIK1 | 1.782800069 | 0.671002185 | 4.074227422 | 0.000135659 | 0.003537053 | 0.800259854 |
| FREQ | -1.127885324 | 0.116936829 | -4.070104129 | 0.000137557 | 0.003564821 | 0.787255535 |
| LOC730994 | 0.556426113 | 0.147355733 | 4.044787732 | 0.000149789 | 0.003781063 | 0.707572892 |
| IL1B | 1.211923786 | 0.505319806 | 4.043729616 | 0.000150323 | 0.003781063 | 0.704248591 |
| CST7 | 0.714938617 | 0.135343503 | 4.034912126 | 0.00015484 | 0.003827144 | 0.676565617 |
| SFRS7 | -0.789004596 | 0.011283606 | -4.03052235 | 0.000157137 | 0.003840177 | 0.662796419 |
| RALGAPA1 | -0.524662147 | 0.049455748 | -4.03047291 | 0.000157163 | 0.003840177 | 0.662641389 |
| HSPA6 | 0.702727369 | 0.027394608 | 4.024280624 | 0.000160461 | 0.003876439 | 0.643232914 |
| CYP26B1 | -0.770279259 | 0.168448214 | -4.021500289 | 0.000161963 | 0.003890744 | 0.634524014 |
| WFDC1 | -1.137111298 | 0.095844017 | -4.01463575 | 0.000165729 | 0.003936995 | 0.613036725 |
| TNFAIP8L1 | -0.602309637 | 0.032558838 | -4.002662618 | 0.000172502 | 0.004075248 | 0.575608606 |
| ACMSD | 0.521221946 | 0.118861114 | 3.995178012 | 0.000176871 | 0.004132785 | 0.552244073 |
| RPPH1 | 0.853659158 | 0.130367122 | 3.98717007 | 0.000181663 | 0.004221684 | 0.527273569 |
| PDIA4 | -0.523406345 | 0.050108949 | -3.984829605 | 0.000183087 | 0.004231777 | 0.519980915 |
| LOC652479 | 0.561640741 | 0.060310041 | 3.96564531 | 0.000195171 | 0.004415613 | 0.46029741 |
| FAM38A | -0.53606029 | 0.043351705 | -3.960886055 | 0.000198286 | 0.00446247 | 0.44551679 |
| SHC4 | -0.750282613 | 0.118502526 | -3.954532201 | 0.000202519 | 0.004510256 | 0.425799888 |
| CX3CR1 | 0.664108628 | 0.254588645 | 3.923692181 | 0.00022433 | 0.00494451 | 0.330360295 |
| VGLL3 | -0.68726143 | 0.034779251 | -3.889835675 | 0.000250874 | 0.005390632 | 0.22608944 |
| PROK2 | 0.850284099 | 0.192011759 | 3.857776648 | 0.000278772 | 0.005814776 | 0.127846674 |
| C16orf56 | -0.571095549 | 0.030579598 | -3.843962068 | 0.000291691 | 0.005939387 | 0.085661973 |
| PACAP | 0.577904513 | 0.178973869 | 3.81909077 | 0.000316412 | 0.00632233 | 0.009942635 |
| MRPS31 | -0.672153642 | 0.007463308 | -3.815865153 | 0.000319762 | 0.006354942 | 0.00014406 |
| BAZ2B | -0.525844311 | 0.00876348 | -3.810927583 | 0.000324955 | 0.006372733 | -0.014845305 |
| SLC9A9 | -0.59023565 | 0.080361534 | -3.80958025 | 0.000326386 | 0.006372733 | -0.018933477 |
| TBC1D3G | 0.542465808 | 0.053060511 | 3.798428346 | 0.000338466 | 0.006507917 | -0.052737804 |
| STRN3 | -0.680160849 | 0.032712147 | -3.796209877 | 0.00034092 | 0.006507917 | -0.05945541 |
| DPY19L4 | -0.526782184 | 0.010632291 | -3.774466022 | 0.000365888 | 0.006898747 | -0.125170397 |
| SSFA2 | 0.511103542 | 0.029976218 | 3.774175328 | 0.000366234 | 0.006898747 | -0.126047387 |
| DYSF | 0.649518544 | 0.151700639 | 3.765803695 | 0.000376315 | 0.006958287 | -0.151285903 |
| UBLCP1 | -0.695714665 | 0.028631102 | -3.765619823 | 0.00037654 | 0.006958287 | -0.151839848 |
| NDFIP2 | -0.61035409 | 0.014740401 | -3.764808907 | 0.000377531 | 0.006958287 | -0.154282681 |
| PADI4 | 0.937419076 | 0.346007432 | 3.759582462 | 0.000383979 | 0.007046758 | -0.17001928 |
| SELL | 0.961193938 | 0.41839917 | 3.757231937 | 0.000386913 | 0.007070262 | -0.17709225 |
| FAM179B | -0.558565588 | 0.016328694 | -3.75111625 | 0.000394649 | 0.007156947 | -0.195482268 |
| CRY1 | -0.626046494 | 0.059078254 | -3.748244533 | 0.000398332 | 0.007156947 | -0.204111248 |
| WAS | 0.731305245 | 0.050637249 | 3.743745628 | 0.000404168 | 0.007178121 | -0.217621469 |
| LOC285053 | -1.000555703 | 0.001279214 | -3.740303551 | 0.000408688 | 0.007178121 | -0.227951298 |
| MRPL15 | -0.810290753 | 0.031999218 | -3.739611724 | 0.000409603 | 0.007178121 | -0.230026797 |
| CHMP2B | -0.718878696 | 0.073545461 | -3.734758679 | 0.000416072 | 0.007261735 | -0.244579409 |
| CXCL1 | 0.672398703 | 0.296917478 | 3.712738352 | 0.000446676 | 0.007609501 | -0.310464125 |
| GDPD5 | -0.545755565 | 0.070612502 | -3.706657524 | 0.000455499 | 0.007698469 | -0.328615408 |
| ZNF296 | 0.664892237 | 0.175731571 | 3.696069111 | 0.000471261 | 0.007871794 | -0.360177708 |
| C5orf41 | -0.806873607 | 0.023688938 | -3.685959912 | 0.000486796 | 0.008030024 | -0.390259053 |
| TMEM14A | -0.840216446 | 0.011568491 | -3.685458004 | 0.00048758 | 0.008030024 | -0.391751212 |
| PKD1 | -0.687893643 | 0.02930965 | -3.685022297 | 0.000488262 | 0.008030024 | -0.393046453 |
| BTF3L4 | -0.615028373 | 0.012649342 | -3.673746637 | 0.000506217 | 0.008230361 | -0.426532691 |
| CMBL | -0.661697778 | 0.056942452 | -3.649715858 | 0.000546612 | 0.008746371 | -0.497683856 |
| EFNB2 | -0.917495195 | 0.010821461 | -3.647760505 | 0.000550031 | 0.008746371 | -0.503460397 |
| TUBB6 | -0.616419629 | 0.054018463 | -3.647648115 | 0.000550228 | 0.008746371 | -0.503792362 |
| SRPX2 | -0.727841038 | 0.047008135 | -3.627294135 | 0.000587062 | 0.009230718 | -0.563805079 |
| NAP1L3 | -0.538704875 | 0.026056874 | -3.624908971 | 0.000591529 | 0.009230718 | -0.570823692 |
| SNAI2 | -0.662336809 | 0.025712646 | -3.622251636 | 0.000596544 | 0.009230718 | -0.578639748 |
| PPP3CC | -0.577117129 | 0.028848726 | -3.621851474 | 0.000597303 | 0.009230718 | -0.579816436 |
| LOC391045 | 0.650605711 | 0.094502231 | 3.62174794 | 0.000597499 | 0.009230718 | -0.580120865 |
| ARL17P1 | 0.838358763 | 0.174640378 | 3.615553759 | 0.000609365 | 0.009339229 | -0.598324142 |
| CD22 | 0.626763525 | 0.21575565 | 3.603951605 | 0.000632198 | 0.009557205 | -0.632366819 |
| IL6 | 1.771075475 | 0.834741714 | 3.586067783 | 0.000668998 | 0.009967364 | -0.68470401 |
| USP38 | -0.691252001 | 0.005975628 | -3.579639675 | 0.000682716 | 0.010136441 | -0.703475171 |
| ITGAL | 0.666739714 | 0.136749602 | 3.573929276 | 0.000695128 | 0.010249539 | -0.720132355 |
| LOC653158 | 0.775064015 | 0.269663565 | 3.55268582 | 0.000743213 | 0.010736408 | -0.781948842 |
| LOC646483 | -0.832694581 | 0.053855014 | -3.543640247 | 0.000764634 | 0.010935675 | -0.808198304 |
| OSM | 0.896126637 | 0.359387888 | 3.543621492 | 0.000764679 | 0.010935675 | -0.808252685 |
| OLFML3 | -0.732187364 | 0.079584348 | -3.534459068 | 0.000786976 | 0.011179767 | -0.834796904 |
| RAB7L1 | -0.689224613 | 0.084484216 | -3.532891275 | 0.000790853 | 0.011197638 | -0.839334443 |
| CNTN6 | 0.522056484 | 0.093207871 | 3.514474612 | 0.000837773 | 0.011742316 | -0.89253818 |
| CXCL5 | 0.637933476 | 0.093764832 | 3.513508543 | 0.000840306 | 0.011742316 | -0.895324044 |
| BTG2 | 0.902795252 | 0.328084149 | 3.506888871 | 0.000857859 | 0.011871209 | -0.914399781 |
| DVL3 | 0.549255831 | 0.141061574 | 3.495037471 | 0.000890157 | 0.01216074 | -0.948492811 |
| SESTD1 | -0.552069446 | 0.0334228 | -3.484076281 | 0.000921055 | 0.012502951 | -0.97995752 |
| IRF2BP2 | -0.530167423 | 0.061457942 | -3.473672548 | 0.00095132 | 0.01279197 | -1.009761827 |
| S100A12 | 0.739856557 | 0.190320823 | 3.47109591 | 0.000958961 | 0.012814114 | -1.017134222 |
| LOC100008589 | -2.325705901 | 0.458585493 | -3.465483483 | 0.000975805 | 0.012998573 | -1.033180251 |
| MGC87042 | -0.873177903 | 0.021442056 | -3.459192477 | 0.000995019 | 0.013127602 | -1.05114594 |
| SPIB | 0.680833794 | 0.21601317 | 3.455399826 | 0.001006776 | 0.013127602 | -1.061966447 |
| SLC2A3 | 1.091781742 | 0.430348288 | 3.455201757 | 0.001007393 | 0.013127602 | -1.062531327 |
| MGC18216 | -0.533937984 | 0.04064357 | -3.452392387 | 0.001016192 | 0.013127602 | -1.070541138 |
| SH3PXD2B | -0.527715339 | 0.033293901 | -3.445928988 | 0.001036712 | 0.013209216 | -1.088952556 |
| SATB1 | 0.587545024 | 0.077501178 | 3.443845798 | 0.001043408 | 0.013239213 | -1.094881776 |
| CXCR7 | -0.82217257 | 0.061147442 | -3.434355753 | 0.001074439 | 0.013462101 | -1.121862369 |
| HBG2 | 1.566228477 | 0.836172761 | 3.434156943 | 0.001075098 | 0.013462101 | -1.122427063 |
| PHRF1 | 0.654995273 | 0.081468682 | 3.433669525 | 0.001076716 | 0.013462101 | -1.123811423 |
| SOLH | 0.504311306 | 0.082673089 | 3.427444088 | 0.001097587 | 0.01365157 | -1.141481299 |
| ITM2C | -0.821952614 | 0.096172075 | -3.42090471 | 0.001119922 | 0.01380054 | -1.160019195 |
| MRPS17 | -0.542022028 | 0.025453098 | -3.416227206 | 0.001136162 | 0.013880651 | -1.173264514 |
| MNDA | 0.540534257 | 0.167846389 | 3.41079165 | 0.001155313 | 0.014034431 | -1.188641174 |
| LENG8 | 0.526152095 | 0.190493752 | 3.404417463 | 0.001178161 | 0.014111536 | -1.206652222 |
| KDM6B | 0.537471987 | 0.143048838 | 3.369367517 | 0.001311634 | 0.015253225 | -1.305284742 |
| BNC2 | -0.504612805 | 0.045284079 | -3.359157031 | 0.00135313 | 0.015564466 | -1.333887835 |
| IL2RB | 0.608026247 | 0.258578383 | 3.345634464 | 0.001409997 | 0.015866179 | -1.37167848 |
| RAB7B | 0.675666925 | 0.079972483 | 3.343168353 | 0.001420609 | 0.015943628 | -1.378559182 |
| NCF2 | 0.822560345 | 0.142993673 | 3.341136586 | 0.001429408 | 0.016000385 | -1.384225422 |
| DUSP2 | 0.884532129 | 0.393177193 | 3.334932976 | 0.001456594 | 0.016177648 | -1.401511667 |
| GPX7 | -0.576398196 | 0.028660217 | -3.325413424 | 0.001499262 | 0.016522797 | -1.42799507 |
| CD7 | 0.726871809 | 0.251063311 | 3.310499337 | 0.00156849 | 0.017022494 | -1.469381864 |
| AOX1 | -0.913793295 | 0.022828259 | -3.308659161 | 0.001577238 | 0.017074096 | -1.474479536 |
| ANGPTL6 | 0.561861185 | 0.057421569 | 3.302831283 | 0.001605246 | 0.017331788 | -1.490611123 |
| PDIA6 | 0.581955886 | 0.052156447 | 3.294719584 | 0.001645013 | 0.017585188 | -1.513031772 |
| LOC390466 | -0.716788047 | 0.185244966 | -3.287938727 | 0.001678966 | 0.017858852 | -1.531744838 |
| ARIH2 | -0.529055848 | 0.024591855 | -3.279904133 | 0.001720052 | 0.018219225 | -1.553883381 |
| G0S2 | 0.897585726 | 0.335968827 | 3.279649855 | 0.001721367 | 0.018219225 | -1.554583407 |
| SIGLEC10 | -0.65256563 | 0.067516172 | -3.273544387 | 0.001753243 | 0.01845776 | -1.57138049 |
| C13orf18 | 0.528651781 | 0.14449879 | 3.272859276 | 0.001756854 | 0.01845776 | -1.573263986 |
| C11orf46 | -0.669166395 | 0.061369941 | -3.271229615 | 0.001765472 | 0.018502844 | -1.577743122 |
| MTMR4 | -0.583334007 | 0.135567658 | -3.265072631 | 0.001798394 | 0.018755934 | -1.594651684 |
| PLVAP | -0.717427563 | 0.075162151 | -3.256155306 | 0.001847102 | 0.019001133 | -1.619101614 |
| LOC649366 | -1.214481866 | 0.006824849 | -3.255890516 | 0.001848567 | 0.019001133 | -1.619826918 |
| ARHGEF18 | -0.654230871 | 0.032289164 | -3.25378782 | 0.00186024 | 0.019075269 | -1.625585091 |
| UFM1 | -0.514766964 | 0.006424728 | -3.247089421 | 0.001897892 | 0.019368461 | -1.643911206 |
| MGC33556 | 0.509832993 | 0.003776058 | 3.222220811 | 0.002044044 | 0.020565484 | -1.711718478 |
| SCARNA9 | -0.530523811 | 0.037155969 | -3.219380031 | 0.002061396 | 0.02064293 | -1.719440998 |
| LOC440776 | 0.578427783 | 0.127728683 | 3.211515399 | 0.002110162 | 0.021032757 | -1.740795684 |
| C5orf46 | -1.135310924 | 0.054304903 | -3.191937616 | 0.002236305 | 0.021882016 | -1.793795324 |
| ADORA3 | -0.675174762 | 0.053615791 | -3.171818923 | 0.002373286 | 0.022652167 | -1.848020912 |
| ITK | 0.779138062 | 0.482091678 | 3.161771822 | 0.002444603 | 0.023024503 | -1.875009767 |
| FGFBP2 | 0.541948872 | 0.105149541 | 3.160376953 | 0.002454662 | 0.023043903 | -1.878751906 |
| PLAC8 | 0.730502126 | 0.32550158 | 3.159992467 | 0.002457441 | 0.023043903 | -1.879783195 |
| TWIST1 | -0.543329723 | 0.10400086 | -3.153872959 | 0.002502078 | 0.023325946 | -1.896185253 |
| RNGTT | -0.559727101 | 0.037026585 | -3.127705735 | 0.002701653 | 0.024480518 | -1.966065286 |
| DR1 | -0.534176744 | 0.060634187 | -3.122373136 | 0.002744112 | 0.024754899 | -1.98025504 |
| RDH10 | -0.558924332 | 0.126570393 | -3.112837098 | 0.002821599 | 0.025293834 | -2.00558665 |
| LOC402644 | -0.778031605 | 0.009561852 | -3.105698894 | 0.002880937 | 0.025771732 | -2.024512229 |
| P2RX7 | -0.713069206 | 0.104014785 | -3.09864456 | 0.002940723 | 0.026142478 | -2.043184743 |
| GBP5 | 0.525452027 | 0.14728012 | 3.09311904 | 0.00298836 | 0.026338859 | -2.0577892 |
| HBG1 | 1.412928516 | 0.831620089 | 3.092514907 | 0.002993612 | 0.026338859 | -2.059384838 |
| VENTX | -0.586876241 | 0.013099324 | -3.073087229 | 0.003167175 | 0.027099451 | -2.110577289 |
| BCL11A | 0.512314761 | 0.23185713 | 3.071687414 | 0.003180037 | 0.027099451 | -2.114256824 |
| SLC16A9 | -0.663797869 | 0.037492162 | -3.069474071 | 0.003200473 | 0.027099451 | -2.120072315 |
| CHMP5 | -0.759338871 | 0.172087084 | -3.061809132 | 0.003272197 | 0.027489025 | -2.140188212 |
| ATP13A1 | 0.711950375 | 0.246247868 | 3.047234667 | 0.003412732 | 0.028335614 | -2.1783367 |
| TMEM130 | -0.647637661 | 0.079737768 | -3.037778857 | 0.0035069 | 0.028893072 | -2.203016279 |
| LOC643357 | -1.110443311 | 0.013701402 | -3.032349029 | 0.003562064 | 0.029165977 | -2.217162789 |
| LRMP | 0.695968602 | 0.455225255 | 3.027246841 | 0.003614635 | 0.029193791 | -2.23043884 |
| FAM129C | 0.579791035 | 0.244225857 | 3.022846324 | 0.003660556 | 0.029367336 | -2.241875989 |
| IER3 | 0.986446057 | 0.234307555 | 3.01892579 | 0.003701926 | 0.029557685 | -2.252055384 |
| IL2RG | 0.582402479 | 0.087607352 | 3.018628592 | 0.003705079 | 0.029557685 | -2.252826643 |
| USP36 | 0.61980484 | 0.273416812 | 2.999020664 | 0.003918766 | 0.030478447 | -2.303588112 |
| CH25H | 0.525025286 | 0.201021501 | 2.996130721 | 0.003951214 | 0.030552242 | -2.311049109 |
| LOC651898 | 0.863485493 | 0.465220479 | 2.975898072 | 0.004185476 | 0.031732438 | -2.363135406 |
| TUBB1 | 0.695590235 | 0.192557442 | 2.967233957 | 0.004289688 | 0.03223674 | -2.385360286 |
| MYO5A | -0.515084007 | 0.092007316 | -2.955359236 | 0.004436436 | 0.03310681 | -2.415742904 |
| BHLHB2 | 0.705214727 | 0.367900673 | 2.945399184 | 0.004563104 | 0.033790134 | -2.441156897 |
| LOC136143 | -0.554556394 | 0.003034459 | -2.944444627 | 0.004575418 | 0.033790134 | -2.443589189 |
| PRKCQ | 0.629812659 | 0.339725557 | 2.917834393 | 0.004931368 | 0.035761179 | -2.511157632 |
| ACTA1 | -0.789496054 | 0.022606184 | -2.91316148 | 0.004996464 | 0.036028466 | -2.522975732 |
| LOC388654 | -1.049552524 | 0.013680048 | -2.903233711 | 0.005137413 | 0.036732512 | -2.548036603 |
| OS9 | 0.51721905 | 0.271376481 | 2.902661766 | 0.005145644 | 0.036732512 | -2.549478422 |
| NCF1 | 0.559458183 | 0.195136797 | 2.901758012 | 0.005158676 | 0.036764162 | -2.551756264 |
| CCR7 | 0.977478286 | 0.544290376 | 2.897764076 | 0.005216632 | 0.036930991 | -2.561816296 |
| NUP210 | 0.608932318 | 0.205423848 | 2.895984979 | 0.005242642 | 0.037053783 | -2.566294184 |
| IL18RAP | 0.577044469 | 0.214854081 | 2.894347565 | 0.005266687 | 0.037130035 | -2.570413636 |
| SHKBP1 | 0.552257209 | 0.097487762 | 2.893479131 | 0.005279481 | 0.037130035 | -2.572597756 |
| ANKRD13A | -0.554856055 | 0.063802026 | -2.890254251 | 0.005327243 | 0.037343102 | -2.58070405 |
| SOCS3 | 0.887669622 | 0.390688042 | 2.887667196 | 0.005365847 | 0.037469498 | -2.587202143 |
| C6orf173 | -0.556803876 | 0.104925956 | -2.887541827 | 0.005367725 | 0.037469498 | -2.58751693 |
| PBX3 | -0.544561605 | 0.015525371 | -2.883794093 | 0.005424126 | 0.037713112 | -2.596922313 |
| LTB | 0.891848297 | 0.389533646 | 2.854689659 | 0.005881148 | 0.039790807 | -2.669649385 |
| P2RY8 | 0.626119381 | 0.27519863 | 2.853455067 | 0.0059013 | 0.039864071 | -2.672722085 |
| SOCS2 | -0.508794241 | 0.03013031 | -2.845043115 | 0.006040307 | 0.040546866 | -2.693631241 |
| KIAA0125 | 0.577284695 | 0.312348706 | 2.828814389 | 0.006317063 | 0.041499014 | -2.73383762 |
| CRIPAK | 0.628079674 | 0.151280497 | 2.82292587 | 0.006420341 | 0.041973059 | -2.748383033 |
| RARRES1 | -0.505769914 | 0.067226361 | -2.818721944 | 0.006495023 | 0.042336464 | -2.758753145 |
| IL8 | 1.168714304 | 0.540731995 | 2.816178629 | 0.006540593 | 0.042568608 | -2.765021189 |
| GFPT2 | 0.739452542 | 0.501435503 | 2.8140644 | 0.006578698 | 0.042735881 | -2.770228458 |
| GPR137B | -0.636464362 | 0.028408987 | -2.812310086 | 0.006610472 | 0.042735881 | -2.774547 |
| TNFSF10 | -0.650786853 | 0.033350371 | -2.810992445 | 0.00663443 | 0.042788571 | -2.777789246 |
| HBD | 0.682730818 | 0.19114116 | 2.776312556 | 0.007294474 | 0.045713685 | -2.862705398 |
| FAM65B | 0.63046418 | 0.380508789 | 2.760031754 | 0.007624714 | 0.047046574 | -2.902290477 |
| DNAJB9 | -0.515754274 | 0.049727689 | -2.753643788 | 0.007757997 | 0.047668654 | -2.917773093 |
| CLEC11A | 0.504371376 | 0.239865882 | 2.747159728 | 0.00789547 | 0.048024224 | -2.933460262 |
| CCL3L3 | 0.974419861 | 0.187366671 | 2.742787065 | 0.007989436 | 0.048320834 | -2.944023084 |
| C17orf91 | -0.654537442 | 0.042768869 | -2.732179309 | 0.008221671 | 0.049306962 | -2.969593535 |
| S100P | 0.65278596 | 0.183409815 | 2.728944222 | 0.008293718 | 0.049409696 | -2.97737656 |
