# Supplementary material for: Single-Cell Sequencing Analysis and Multiple Machine Learning Methods Identified G0S2 and HPSE as Novel Biomarkers for Abdominal Aortic Aneurysm
Source: Front Immunol. 2022 Jun 13;13:907309. doi: 10.3389/fimmu.2022.907309 (PMC9234288; doi:10.3389/fimmu.2022.907309)
Supplement: Supplementary Table 5 — DEGs between large AAA and small AAA samples. [file Table_5.doc]

**Supplementary table 5**

| Genes | logFC | AveExpr | t | P.Value | adj.P.Val | B |
| --- | --- | --- | --- | --- | --- | --- |
| CLEC5A | 0.640789066 | 0.20087917 | 2.528790857 | 0.014568872 | 0.999947753 | -3.505292836 |
| HPSE | -0.23130159 | 0.024768716 | -2.458765283 | 0.017361916 | 0.999947753 | -3.576020971 |
| ZBP1 | -0.438145317 | 0.389035433 | -2.336966879 | 0.023389842 | 0.999947753 | -3.695820372 |
| OSM | -0.509599937 | 0.519647093 | -2.329222364 | 0.023829972 | 0.999947753 | -3.703294963 |
| DNM3 | -0.213858784 | 0.173971274 | -2.290568297 | 0.026139144 | 0.999947753 | -3.740339723 |
| TNFAIP3 | -0.582158397 | 0.515167843 | -2.280624787 | 0.026764483 | 0.999947753 | -3.749798029 |
| HNRPH1 | -0.263384795 | 0.184425701 | -2.280607364 | 0.02676559 | 0.999947753 | -3.749814575 |
| EGR3 | -0.295235019 | 0.268497445 | -2.270547542 | 0.027411765 | 0.999947753 | -3.759353544 |
| CORO7 | -0.288229313 | 0.209065302 | -2.247457848 | 0.028947538 | 0.999947753 | -3.78113321 |
| LBA1 | 0.242547376 | 0.234100418 | 2.175062826 | 0.034268125 | 0.999947753 | -3.84837013 |
| CYR61 | -0.657637345 | 0.026027523 | -2.125079532 | 0.038426788 | 0.999947753 | -3.893842799 |
| OVGP1 | -0.238477873 | 0.129809729 | -2.124069114 | 0.038515226 | 0.999947753 | -3.894753898 |
| HLA-DQB2 | -0.357381236 | 0.159043719 | -2.096118509 | 0.041033204 | 0.999947753 | -3.919827877 |
| STX7 | -0.121900418 | 0.007925062 | -2.070965001 | 0.043420751 | 0.999947753 | -3.94217783 |
| APLP2 | -0.28667936 | 0.023518301 | -2.058961136 | 0.044602126 | 0.999947753 | -3.952771415 |
| PTPRB | 0.202400773 | 0.061885591 | 2.027489951 | 0.047832772 | 0.999947753 | -3.980321213 |
| DAAM2 | -0.171644315 | 0.090864279 | -2.01167132 | 0.049531748 | 0.999947753 | -3.99404539 |
