# Supplementary material for: Single-Cell Sequencing Analysis and Multiple Machine Learning Methods Identified G0S2 and HPSE as Novel Biomarkers for Abdominal Aortic Aneurysm
Source: Front Immunol. 2022 Jun 13;13:907309. doi: 10.3389/fimmu.2022.907309 (PMC9234288; doi:10.3389/fimmu.2022.907309)
Supplement: Supplementary Table 6 — Results of immune cell infiltration between AAA and normal samples. [file Table_6.doc]

**Supplementary table 6**

| Samples | B cells naive | B cells memory | Plasma cells | T cells CD8 | T cells CD4 naive | T cells CD4 memory resting | T cells CD4 memory activated | T cells follicular helper | T cells regulatory (Tregs) | T cells gamma delta | NK cells resting |
| --- | --- | --- | --- | --- | --- | --- | --- | --- | --- | --- | --- |
| GSM1386844_con | 0 | 0.016493959 | 0 | 0 | 0.049111184 | 0 | 0.0008182 | 0.004271779 | 0.002357113 | 0.050665434 | 0.025262478 |
| GSM1386845_con | 0 | 0.069102372 | 0.151642305 | 0.147739729 | 0 | 0.178933226 | 0 | 0 | 0 | 0.013033924 | 0 |
| GSM1386850_con | 0.018122228 | 0.087797987 | 0.388430624 | 0.11700717 | 0.016966586 | 0.070944266 | 0.156562692 | 0 | 0 | 0 | 0 |
| GSM1386784_treat | 0 | 0.288694498 | 0.023518005 | 0 | 0.031759174 | 0.125553292 | 0 | 0 | 0.038267226 | 0.060506958 | 0 |
| GSM1386785_treat | 0.178609638 | 0.117366737 | 0.02244389 | 0 | 0.222551782 | 0 | 0.098266936 | 0.08209211 | 0 | 0.103654432 | 0.003673942 |
| GSM1386786_treat | 0.049156494 | 0.146314559 | 0.064666325 | 0 | 0.160909841 | 0.036836287 | 0.011715093 | 0.029922782 | 0 | 0.163029456 | 0 |
| GSM1386787_treat | 0 | 0.119462256 | 0.309388002 | 0 | 0.019186074 | 0.099479633 | 0.073718404 | 0.010863744 | 0 | 0.134915378 | 0 |
| GSM1386792_treat | 0 | 0.006753802 | 0.0022285 | 0 | 0 | 0 | 0 | 0.050108999 | 0.074057329 | 0.072221294 | 0 |
| GSM1386793_treat | 0.133467727 | 0 | 0.04543633 | 0.147381863 | 0.122864223 | 0 | 0.060005639 | 0.108650047 | 0.025277853 | 0.196706728 | 0 |
| GSM1386794_treat | 0.008807511 | 0.000328165 | 0.008068003 | 0 | 0.015157531 | 0.098125433 | 0 | 0.020944532 | 0.019740679 | 0.017173851 | 0 |
| GSM1386795_treat | 0 | 0.055415016 | 0 | 0.051916762 | 0.015684012 | 0 | 0 | 0.018337619 | 0.043881535 | 0 | 0.061158222 |
| GSM1386796_treat | 0.101914008 | 0.141907296 | 0 | 0.04257203 | 0.292651013 | 0 | 0 | 0.17126784 | 0.022246309 | 0 | 0.021085996 |
| GSM1386800_treat | 0.176923667 | 0 | 0.112724353 | 0.067203741 | 0.08157883 | 0 | 0.035843573 | 0.152996169 | 0.034891571 | 0.091139901 | 0 |
| GSM1386802_treat | 0 | 0.178546724 | 0.352857406 | 0 | 0.09239154 | 0 | 0.037913389 | 0.10751157 | 0.064313574 | 0 | 0 |
| GSM1386803_treat | 0 | 0.122964069 | 0.030336143 | 0 | 0 | 0.025876427 | 0 | 0.127843645 | 0 | 0.246465758 | 0 |
| GSM1386804_treat | 0.185579364 | 0.049865902 | 0.059100449 | 0.070451895 | 0.118567397 | 0.0482946 | 0 | 0.02874415 | 0 | 0 | 0.017382618 |
| GSM1386805_treat | 0 | 0.665469319 | 0 | 0 | 0.099942048 | 0 | 0 | 0 | 0 | 0.001202936 | 0.092809689 |
| GSM1386806_treat | 0.126663787 | 0 | 0.148475002 | 0 | 0.076307901 | 0.108513598 | 0.143781096 | 0 | 0 | 0 | 0 |
| GSM1386807_treat | 0.213801621 | 0 | 0.288407572 | 0.057998061 | 0.074499376 | 0 | 0.059305057 | 0.080500597 | 0 | 0.04265218 | 0 |
| GSM1386808_treat | 0.097205203 | 0 | 0.258694226 | 0.034115507 | 0 | 0.08562671 | 0.18274073 | 0 | 0 | 0.091532422 | 0 |
| GSM1386809_treat | 0 | 0.048592622 | 0.547275654 | 0.012680857 | 0.078123672 | 0.008784152 | 0.024874527 | 0 | 0 | 0 | 0 |
| GSM1386810_treat | 0 | 0.072039507 | 0 | 0 | 0 | 0.194807324 | 0 | 0.135047328 | 0 | 0.077448898 | 0 |
| GSM1386811_treat | 0 | 0.180395183 | 0.066840528 | 0.033992198 | 0 | 0.19674707 | 0.075115433 | 0.085069932 | 0 | 0.108059441 | 0 |
| GSM1386814_treat | 0 | 0 | 0.104859182 | 0 | 0 | 0.068620167 | 0 | 0.031182791 | 0 | 0.044286423 | 0 |
| GSM1386815_treat | 0 | 0.054487344 | 0.093827432 | 0 | 0 | 0.102015404 | 0 | 0.073505898 | 0 | 0.179347283 | 0 |
| GSM1386816_treat | 0 | 0.283175564 | 0 | 0.015434438 | 0.222736741 | 0 | 0 | 0.130992148 | 0.077786661 | 0 | 0 |
| GSM1386818_treat | 0.162178623 | 0.046148665 | 0.101360878 | 0.000974619 | 0.207252105 | 0 | 0 | 0.273993146 | 0.022323403 | 0.013302965 | 0 |
| GSM1386819_treat | 0.032496077 | 0 | 0 | 0 | 0.063104037 | 0 | 0.008535454 | 0 | 0.274780457 | 0 | 0.039479186 |
| GSM1386822_treat | 0 | 0.091208428 | 0.070819037 | 0.036794068 | 0 | 0 | 0 | 0.036037969 | 0.08975071 | 0 | 0 |
| GSM1386823_treat | 0 | 0 | 0 | 0 | 0 | 0.074400648 | 0 | 0 | 0 | 0 | 0.006527737 |
| GSM1386824_treat | 0.043714488 | 0 | 0.077308655 | 0.044075291 | 0 | 0 | 0.074931485 | 0.068075649 | 0 | 0 | 0.049804629 |
| GSM1386825_treat | 0.117525829 | 0 | 0.27473773 | 0.034459676 | 0.032276733 | 0 | 0 | 0.117442119 | 0.005849781 | 0 | 0 |
| GSM1386826_treat | 0.239325149 | 0.027037456 | 0.139373184 | 0.103197591 | 0.110171902 | 0 | 0 | 0.071405685 | 0.053901587 | 0 | 0 |
| GSM1386827_treat | 0.072187557 | 0.04675707 | 0.297396763 | 0.02161945 | 0.050548228 | 0 | 0.055544101 | 0.081946583 | 0.048926724 | 0 | 0.054574735 |
| GSM1386829_treat | 0.041914483 | 0.02150337 | 0.148728836 | 0.122680072 | 0.054957554 | 0 | 0.086714807 | 0.06454988 | 0 | 0 | 0.015135628 |

| Samples | NK cells activated | Monocytes | Macrophages M0 | Macrophages M1 | Macrophages M2 | Dendritic cells resting | Dendritic cells activated | Mast cells resting | Mast cells activated | Eosinophils | Neutrophils |
| --- | --- | --- | --- | --- | --- | --- | --- | --- | --- | --- | --- |
| GSM1386844_con | 0 | 0 | 0.35955558 | 0.154399132 | 0.194968965 | 0.025218016 | 0 | 0 | 0.110654762 | 0.006223396 | 0 |
| GSM1386845_con | 0.051305905 | 0.020784219 | 0 | 0.036692809 | 0.267432754 | 0 | 0 | 0.023824433 | 0.039508324 | 0 | 0 |
| GSM1386850_con | 0.014654129 | 0 | 0 | 0.0376536 | 0.001298246 | 0.052190141 | 0 | 0.038372332 | 0 | 0 | 0 |
| GSM1386784_treat | 0.043279968 | 0.004249435 | 0 | 0.089782518 | 0.042441148 | 0.196718506 | 0 | 0.024534161 | 0.030695112 | 0 | 0 |
| GSM1386785_treat | 0 | 0 | 0.037536369 | 0.006583636 | 0 | 0.004910037 | 0.056463338 | 0.065847154 | 0 | 0 | 0 |
| GSM1386786_treat | 0 | 0.057544784 | 0 | 0.106692113 | 0 | 0.084025713 | 0 | 0.048329357 | 0 | 0.039205875 | 0.00165132 |
| GSM1386787_treat | 0.000426649 | 0.000216199 | 0 | 0.032644014 | 0 | 0.00337176 | 0 | 0 | 0.169656281 | 0 | 0.026671607 |
| GSM1386792_treat | 0 | 0.098048599 | 0.270583424 | 0 | 0.036646979 | 0.045783433 | 0.01931142 | 0 | 0.271792863 | 0.04014571 | 0.01231765 |
| GSM1386793_treat | 0.04874405 | 0 | 0.008565741 | 0.050884784 | 0.016959688 | 0 | 0 | 0.035055327 | 0 | 0 | 0 |
| GSM1386794_treat | 0.015492977 | 0.074761236 | 0.104098032 | 0.012310037 | 0.067979183 | 4.52E-05 | 0 | 0 | 0.482322463 | 0 | 0.054645205 |
| GSM1386795_treat | 0 | 0.053014694 | 0.15586563 | 0 | 0 | 0 | 0.018118304 | 0 | 0.480442197 | 0 | 0.046166008 |
| GSM1386796_treat | 0.040432889 | 0.014547855 | 0 | 0.002821797 | 0 | 0.086923301 | 0 | 0 | 0.039871154 | 0.021758512 | 0 |
| GSM1386800_treat | 0 | 0 | 0.079588197 | 0 | 0 | 0 | 0.007607726 | 0 | 0.131714821 | 0 | 0.02778745 |
| GSM1386802_treat | 0.026962392 | 0 | 0.04938177 | 0 | 0 | 0 | 0 | 0 | 0.062153084 | 0 | 0.027968552 |
| GSM1386803_treat | 0 | 0 | 0 | 0.172649779 | 0 | 0.199268043 | 0 | 0.074596136 | 0 | 0 | 0 |
| GSM1386804_treat | 0 | 0.072057969 | 0 | 0.036306322 | 0 | 0.093525226 | 0 | 0.205499718 | 0 | 0.014624388 | 0 |
| GSM1386805_treat | 0 | 0 | 0 | 0 | 0 | 0.071043374 | 0.023595898 | 0 | 0.045936735 | 0 | 0 |
| GSM1386806_treat | 0.048853907 | 0.017034915 | 0 | 0.014680255 | 0.021429709 | 0.064166739 | 0 | 0.187726346 | 0 | 0.042366745 | 0 |
| GSM1386807_treat | 0.009661584 | 0.005822151 | 0 | 0.029669193 | 0.008211685 | 0.048527756 | 0 | 0.080943167 | 0 | 0 | 0 |
| GSM1386808_treat | 0.033933087 | 0 | 0 | 0.081927262 | 0.128748275 | 0.005476578 | 0 | 0 | 0 | 0 | 0 |
| GSM1386809_treat | 0.001341164 | 0.056742005 | 0 | 0 | 0.073543861 | 0 | 0 | 0.030715448 | 0.04642183 | 0 | 0.070904208 |
| GSM1386810_treat | 0.025555524 | 0.121565451 | 0 | 0.075460234 | 0 | 0.117136674 | 0 | 0.098734639 | 0 | 0.071409334 | 0.010795088 |
| GSM1386811_treat | 0.099983497 | 0 | 0 | 0.025685983 | 0.016433601 | 0 | 0.022548734 | 0.035071679 | 0 | 0.017061897 | 0.036994825 |
| GSM1386814_treat | 0.007803995 | 0.050466815 | 0 | 0.014637974 | 0.102296902 | 0.11068625 | 0.00078925 | 0 | 0.457802842 | 0 | 0.006567408 |
| GSM1386815_treat | 0.087539641 | 0 | 0 | 0.092566565 | 0.102993137 | 0.028251336 | 0 | 0.129579928 | 0 | 0.018322905 | 0.037563128 |
| GSM1386816_treat | 0.098957323 | 0 | 0.105969287 | 0 | 0.021146046 | 0.016818628 | 0.013184561 | 0 | 0.006921957 | 0.006876647 | 0 |
| GSM1386818_treat | 0.077180356 | 0 | 0 | 0 | 0 | 0 | 0.017112453 | 0 | 0.05832065 | 0.019852137 | 0 |
| GSM1386819_treat | 0.001863002 | 0.000259584 | 0.160616804 | 0.001970571 | 0 | 0 | 0.024053432 | 0 | 0.351675992 | 0 | 0.041165405 |
| GSM1386822_treat | 0 | 0.170623678 | 0.109875499 | 0 | 0 | 0.018737018 | 0 | 0 | 0.310989136 | 0 | 0.065164458 |
| GSM1386823_treat | 0.032630498 | 0.096879066 | 0.240363424 | 0 | 0.293799807 | 0 | 0 | 0 | 0.15775418 | 0 | 0.097644641 |
| GSM1386824_treat | 0 | 0 | 0.204453438 | 0.014084673 | 0.067476764 | 0 | 0 | 0 | 0.333506694 | 0 | 0.022568233 |
| GSM1386825_treat | 0.017331814 | 0.014183565 | 0.128434704 | 0.048314347 | 0 | 0.059144758 | 0.008193768 | 0 | 0.128907565 | 0.013197611 | 0 |
| GSM1386826_treat | 0.015437931 | 0 | 0 | 0 | 0 | 0 | 0.045609964 | 0 | 0.156644004 | 0.031651664 | 0.006243883 |
| GSM1386827_treat | 0 | 0 | 0 | 0 | 0 | 0 | 0.047058584 | 0 | 0.223440206 | 0 | 0 |
| GSM1386829_treat | 0.014770249 | 0.028644877 | 0.036888599 | 0.025462443 | 0.059689936 | 0.017878749 | 0 | 0 | 0.202761952 | 0 | 0.057718565 |
