# Supplementary material for: Single-Cell Sequencing Analysis and Multiple Machine Learning Methods Identified G0S2 and HPSE as Novel Biomarkers for Abdominal Aortic Aneurysm
Source: Front Immunol. 2022 Jun 13;13:907309. doi: 10.3389/fimmu.2022.907309 (PMC9234288; doi:10.3389/fimmu.2022.907309)
Supplement: Supplementary Table 7 — Results of immune cell infiltration between large and small AAA samples. [file Table_7.doc]

**Supplementary table 7**

| Samples | B cells naive | B cells memory | Plasma cells | T cells CD8 | T cells CD4 naive | T cells CD4 memory resting | T cells CD4 memory activated | T cells follicular helper | T cells regulatory (Tregs) | T cells gamma delta | NK cells resting |
| --- | --- | --- | --- | --- | --- | --- | --- | --- | --- | --- | --- |
| GSM1386844_con | 0 | 0.016493961 | 0 | 0 | 0.049111197 | 0 | 0.000818224 | 0.004271785 | 0.002357126 | 0.050665427 | 0.025262511 |
| GSM1386845_con | 0 | 0.068809399 | 0.151750842 | 0.147801075 | 0 | 0.179042484 | 0 | 0 | 0 | 0.012932387 | 0 |
| GSM1386848_con | 0 | 0.01848411 | 0.079567153 | 0.016108414 | 0 | 0.332516003 | 0.068964604 | 0 | 0 | 0.034170008 | 0.015396146 |
| GSM1386850_con | 0.018122243 | 0.087797993 | 0.38843061 | 0.117007177 | 0.016966597 | 0.070944272 | 0.156562659 | 0 | 0 | 0 | 0 |
| GSM1386784_treat | 0 | 0.28869451 | 0.023518007 | 0 | 0.031759176 | 0.125553299 | 0 | 0 | 0.038267226 | 0.060506955 | 0 |
| GSM1386785_treat | 0.17860964 | 0.117366733 | 0.022443891 | 0 | 0.222551781 | 0 | 0.098266937 | 0.08209211 | 0 | 0.103654429 | 0.003673942 |
| GSM1386786_treat | 0.049156492 | 0.146314561 | 0.064666326 | 0 | 0.160909835 | 0.036836295 | 0.011715092 | 0.029922785 | 0 | 0.163029452 | 0 |
| GSM1386787_treat | 0 | 0.119462255 | 0.309387997 | 0 | 0.019186074 | 0.099479635 | 0.073718408 | 0.010863745 | 0 | 0.134915378 | 0 |
| GSM1386792_treat | 0 | 0.006753802 | 0.0022285 | 0 | 0 | 0 | 0 | 0.050109 | 0.074057328 | 0.072221294 | 0 |
| GSM1386793_treat | 0.133465599 | 0 | 0.04545545 | 0.147360798 | 0.122876056 | 0 | 0.060033497 | 0.10862405 | 0.025232853 | 0.19679086 | 0 |
| GSM1386794_treat | 0.00880751 | 0.000328168 | 0.008068003 | 0 | 0.015157531 | 0.098125432 | 0 | 0.020944532 | 0.019740679 | 0.017173851 | 0 |
| GSM1386795_treat | 0 | 0.055415014 | 0 | 0.051916763 | 0.015684012 | 0 | 0 | 0.018337622 | 0.043881534 | 0 | 0.061158223 |
| GSM1386796_treat | 0.101985135 | 0.141909116 | 0 | 0.042593586 | 0.292646092 | 0 | 0 | 0.171289732 | 0.022170498 | 0 | 0.021083998 |
| GSM1386800_treat | 0.176991925 | 0 | 0.112735374 | 0.067131334 | 0.081565758 | 0 | 0.035876675 | 0.152946256 | 0.034899729 | 0.091137878 | 0 |
| GSM1386802_treat | 0 | 0.17854673 | 0.352857406 | 0 | 0.09239154 | 0 | 0.037913389 | 0.107511576 | 0.064313571 | 0 | 0 |
| GSM1386803_treat | 0 | 0.124093322 | 0.030162123 | 0 | 0 | 0.024184316 | 0 | 0.129284256 | 0 | 0.247646195 | 0 |
| GSM1386804_treat | 0.185579353 | 0.049865935 | 0.059100453 | 0.070451866 | 0.118567402 | 0.048294608 | 0 | 0.028744148 | 0 | 0 | 0.017382618 |
| GSM1386805_treat | 0 | 0.665410801 | 0 | 0 | 0.099920383 | 0 | 0 | 0 | 0 | 0.000715349 | 0.093348511 |
| GSM1386806_treat | 0.126663739 | 0 | 0.148474984 | 0 | 0.076307899 | 0.108513591 | 0.143781113 | 0 | 0 | 0 | 0 |
| GSM1386807_treat | 0.213801623 | 0 | 0.288407571 | 0.057998061 | 0.07449937 | 0 | 0.05930506 | 0.080500597 | 0 | 0.04265218 | 0 |
| GSM1386808_treat | 0.09720595 | 0 | 0.258678323 | 0.034114375 | 0 | 0.08563045 | 0.182737064 | 0 | 0 | 0.091551651 | 0 |
| GSM1386809_treat | 0 | 0.048602344 | 0.547225686 | 0.012700758 | 0.078138503 | 0.008802603 | 0.024856983 | 0 | 0 | 0 | 0 |
| GSM1386810_treat | 0 | 0.072068994 | 0 | 0 | 0 | 0.19484231 | 0 | 0.135019112 | 0 | 0.077434058 | 0 |
| GSM1386811_treat | 0 | 0.180395183 | 0.066840528 | 0.033992198 | 0 | 0.196747069 | 0.075115432 | 0.085069932 | 0 | 0.108059442 | 0 |
| GSM1386814_treat | 0 | 0 | 0.104859182 | 0 | 0 | 0.068620167 | 0 | 0.031182791 | 0 | 0.044286423 | 0 |
| GSM1386815_treat | 0 | 0.054487336 | 0.093827438 | 0 | 0 | 0.102015408 | 0 | 0.073505904 | 0 | 0.179347258 | 0 |
| GSM1386816_treat | 0 | 0.283175554 | 0 | 0.01543443 | 0.222736751 | 0 | 0 | 0.13099216 | 0.077786659 | 0 | 0 |
| GSM1386818_treat | 0.162178623 | 0.046148661 | 0.101360879 | 0.000974615 | 0.207252105 | 0 | 0 | 0.273993142 | 0.022323422 | 0.013302971 | 0 |
| GSM1386819_treat | 0.032482327 | 0 | 0 | 0 | 0.063071693 | 0 | 0.008648926 | 0 | 0.274803254 | 0 | 0.039443463 |
| GSM1386822_treat | 0 | 0.091208427 | 0.070819038 | 0.036794067 | 0 | 0 | 0 | 0.03603797 | 0.08975071 | 0 | 0 |
| GSM1386823_treat | 0 | 0 | 0 | 0 | 0 | 0.074400653 | 0 | 0 | 0 | 0 | 0.006527737 |
| GSM1386824_treat | 0.043714482 | 0 | 0.077308654 | 0.04407527 | 0 | 0 | 0.074931493 | 0.068075651 | 0 | 0 | 0.049804644 |
| GSM1386825_treat | 0.117414295 | 0 | 0.274758707 | 0.034439474 | 0.032272283 | 0 | 0 | 0.11748653 | 0.005858272 | 0 | 0 |
| GSM1386826_treat | 0.239325147 | 0.027037472 | 0.139373232 | 0.103197588 | 0.110171905 | 0 | 0 | 0.071405672 | 0.053901578 | 0 | 0 |
| GSM1386827_treat | 0.072187556 | 0.046757071 | 0.297396762 | 0.021619449 | 0.050548227 | 0 | 0.055544099 | 0.08194658 | 0.048926724 | 0 | 0.054574735 |
| GSM1386829_treat | 0.041906491 | 0.02147817 | 0.148731619 | 0.122658228 | 0.054916005 | 0 | 0.086701905 | 0.064584458 | 0 | 0 | 0.015197655 |

| Samples | NK cells activated | Monocytes | Macrophages M0 | Macrophages M1 | Macrophages M2 | Dendritic cells resting | Dendritic cells activated | Mast cells resting | Mast cells activated | Eosinophils | Neutrophils |
| --- | --- | --- | --- | --- | --- | --- | --- | --- | --- | --- | --- |
| GSM1386844_con | 0 | 0 | 0.359555576 | 0.154399117 | 0.194968883 | 0.025218052 | 0 | 0 | 0.110654751 | 0.006223391 | 0 |
| GSM1386845_con | 0.051425576 | 0.020795463 | 0 | 0.036718682 | 0.267408483 | 0 | 0 | 0.023790347 | 0.039525263 | 0 | 0 |
| GSM1386848_con | 0 | 0 | 0 | 0.063731432 | 0.128829964 | 0.017102172 | 0 | 0.153286035 | 0.071843959 | 0 | 0 |
| GSM1386850_con | 0.014654131 | 0 | 0 | 0.037653602 | 0.001298242 | 0.052190142 | 0 | 0.038372332 | 0 | 0 | 0 |
| GSM1386784_treat | 0.043279968 | 0.00424943 | 0 | 0.089782518 | 0.042441154 | 0.196718503 | 0 | 0.02453415 | 0.030695105 | 0 | 0 |
| GSM1386785_treat | 0 | 0 | 0.037536367 | 0.006583635 | 0 | 0.004910038 | 0.056463339 | 0.065847157 | 0 | 0 | 0 |
| GSM1386786_treat | 0 | 0.057544785 | 0 | 0.106692112 | 0 | 0.084025713 | 0 | 0.048329356 | 0 | 0.039205875 | 0.001651322 |
| GSM1386787_treat | 0.000426649 | 0.0002162 | 0 | 0.032644014 | 0 | 0.00337176 | 0 | 0 | 0.169656278 | 0 | 0.026671609 |
| GSM1386792_treat | 0 | 0.098048601 | 0.270583427 | 0 | 0.036646973 | 0.045783437 | 0.01931142 | 0 | 0.27179286 | 0.040145709 | 0.01231765 |
| GSM1386793_treat | 0.048756477 | 0 | 0.00853497 | 0.05091202 | 0.016942637 | 0 | 0 | 0.035014733 | 0 | 0 | 0 |
| GSM1386794_treat | 0.015492977 | 0.074761236 | 0.104098025 | 0.012310037 | 0.067979184 | 4.52E-05 | 0 | 0 | 0.482322467 | 0 | 0.054645205 |
| GSM1386795_treat | 0 | 0.053014694 | 0.155865628 | 0 | 0 | 0 | 0.018118304 | 0 | 0.4804422 | 0 | 0.046166007 |
| GSM1386796_treat | 0.040418252 | 0.014512778 | 0 | 0.002795642 | 0 | 0.086950589 | 0 | 0 | 0.039908648 | 0.021735933 | 0 |
| GSM1386800_treat | 0 | 0 | 0.079581614 | 0 | 0 | 0 | 0.007608399 | 0 | 0.131717678 | 0 | 0.027807379 |
| GSM1386802_treat | 0.026962395 | 0 | 0.049381754 | 0 | 0 | 0 | 0 | 0 | 0.062153086 | 0 | 0.027968554 |
| GSM1386803_treat | 0 | 0 | 0 | 0.172492406 | 0 | 0.198682673 | 0 | 0.073454709 | 0 | 0 | 0 |
| GSM1386804_treat | 0 | 0.072057968 | 0 | 0.03630632 | 0 | 0.093525213 | 0 | 0.205499729 | 0 | 0.014624387 | 0 |
| GSM1386805_treat | 0 | 0 | 0 | 0 | 0 | 0.071004422 | 0.023656499 | 0 | 0.045944035 | 0 | 0 |
| GSM1386806_treat | 0.048853944 | 0.017034916 | 0 | 0.014680233 | 0.021429715 | 0.064166756 | 0 | 0.187726363 | 0 | 0.042366747 | 0 |
| GSM1386807_treat | 0.009661583 | 0.005822151 | 0 | 0.029669193 | 0.008211684 | 0.04852776 | 0 | 0.080943167 | 0 | 0 | 0 |
| GSM1386808_treat | 0.033932235 | 0 | 0 | 0.081938366 | 0.128763967 | 0.005447618 | 0 | 0 | 0 | 0 | 0 |
| GSM1386809_treat | 0.001340338 | 0.056748864 | 0 | 0 | 0.073542833 | 0 | 0 | 0.030721651 | 0.046416798 | 0 | 0.070902638 |
| GSM1386810_treat | 0.025543389 | 0.121538319 | 0 | 0.075475075 | 0 | 0.117127032 | 0 | 0.09873811 | 0 | 0.071413248 | 0.010800353 |
| GSM1386811_treat | 0.099983497 | 0 | 0 | 0.025685984 | 0.016433601 | 0 | 0.022548733 | 0.035071679 | 0 | 0.017061897 | 0.036994825 |
| GSM1386814_treat | 0.007803995 | 0.050466815 | 0 | 0.014637974 | 0.102296902 | 0.110686251 | 0.00078925 | 0 | 0.457802842 | 0 | 0.006567408 |
| GSM1386815_treat | 0.087539639 | 0 | 0 | 0.092566566 | 0.102993121 | 0.028251376 | 0 | 0.129579917 | 0 | 0.018322906 | 0.037563132 |
| GSM1386816_treat | 0.098957328 | 0 | 0.105969287 | 0 | 0.021146044 | 0.016818626 | 0.013184562 | 0 | 0.006921954 | 0.006876647 | 0 |
| GSM1386818_treat | 0.077180342 | 0 | 0 | 0 | 0 | 0 | 0.017112454 | 0 | 0.058320647 | 0.01985214 | 0 |
| GSM1386819_treat | 0.001725864 | 0.000214205 | 0.160548479 | 0.001951711 | 0 | 0 | 0.024061955 | 0 | 0.351780981 | 0 | 0.041267141 |
| GSM1386822_treat | 0 | 0.170623677 | 0.109875506 | 0 | 0 | 0.018737018 | 0 | 0 | 0.310989131 | 0 | 0.065164457 |
| GSM1386823_treat | 0.032630498 | 0.096879065 | 0.240363429 | 0 | 0.2937998 | 0 | 0 | 0 | 0.157754178 | 0 | 0.09764464 |
| GSM1386824_treat | 0 | 0 | 0.204453444 | 0.014084672 | 0.06747676 | 0 | 0 | 0 | 0.333506696 | 0 | 0.022568234 |
| GSM1386825_treat | 0.017338794 | 0.014181671 | 0.128446675 | 0.048349889 | 0 | 0.059156228 | 0.008175396 | 0 | 0.12892714 | 0.013194645 | 0 |
| GSM1386826_treat | 0.015437913 | 0 | 0 | 0 | 0 | 0 | 0.045609958 | 0 | 0.156644011 | 0.031651644 | 0.00624388 |
| GSM1386827_treat | 0 | 0 | 0 | 0 | 0 | 0 | 0.047058584 | 0 | 0.223440213 | 0 | 0 |
| GSM1386829_treat | 0.014745552 | 0.02866351 | 0.036838687 | 0.025482593 | 0.059714067 | 0.017934157 | 0 | 0 | 0.202771723 | 0 | 0.057675181 |
